# Interaction of the polar localization protein BscR with phosphodiesterase PipA regulates virulence strategies in *Pseudomonas aeruginosa*

**DOI:** 10.1016/j.jare.2025.12.048

**Published:** 2025-12-28

**Authors:** Tian Zhou, Anmin Ren, Chenchen Wang, Yizhou Zhang, Jie Deng, Yang Liu, Tianyuan Jia, Haihua Liang, Liang Yang

**Affiliations:** a Ecological Breeding Department, Guangdong Laboratory for Lingnan Modern Agriculture, Heyuan 517000, PR China; b Department of Pharmacology, Joint Laboratory of Guangdong-Hong Kong Universities for Vascular Homeostasis and Diseases, SUSTech Homeostatic Medicine Institute, School of Medicine, Southern University of Science and Technology, Shenzhen 518055, PR China; c Southern University of Science and Technology Hospital, Shenzhen 518055, PR China; d National Clinical Research Center for Infectious Disease, Shenzhen Third People’s Hospital, Shenzhen 518055, PR China

**Keywords:** *Pseudomonas aeruginosa*, Phosphodiesterase, Transcriptional regulation, Protein-protein interaction

## Abstract

**Introduction:**

Bacteria synthesize cyclic diguanylate monophosphate (c-di-GMP) as a central second messenger to control multiple virulence-related phenotypes. In *P. aeruginosa*, while the major function of phosphodiesterases and diguanylate cyclases involves controlling cellular c-di-GMP concentrations, the exact pathways through which these enzymes mediate virulence expression are not fully understood.

**Objectives:**

We aimed to identify interacting partners of phosphodiesterase PipA and elucidate their coordination with PipA in regulating bacterial virulence strategies.

**Methods:**

We employed co-immunoprecipitation and bacterial two-hybrid assays to identify BscR as an interacting partner of PipA. Our systematic investigation combining targeted gene disruption, phenotypic screening, and subcellular protein tracking revealed the regulatory network through which BscR and PipA govern bacterial virulence programs. Finally, we evaluated how BscR influences bacterial pathogenicity using cell infection and *Galleria mellonella* infection models.

**Results:**

Our findings demonstrate that BscR deletion leads to significant reductions in biofilm formation and pyocyanin production, while enhancing bacterial motility. We further reveal that PipA modulates biofilm formation and motility through a BscR-dependent regulatory pathway. Notably, BscR and CheB exhibit co-localization at the cellular poles, with BscR’s polar localization requiring direct interaction with CheB. Importantly, we discovered that elevated PipA levels disrupt the BscR-CheB interaction, thereby attenuating BscR-mediated regulatory functions. Furthermore, BscR mutation significantly enhances bacterial virulence, as evidenced by increased cytotoxicity in A549 cell infection and *Galleria mellonella* infection assays.

**Conclusion:**

These findings elucidate that BscR in *P. aeruginosa* serves as a crucial regulatory factor in modulating bacterial virulence determinants. This study provides mechanistic insights into BscR-mediated virulence regulation, offering a framework to design innovative approaches targeting infection control.

## Highlights

• BscR is a polar-localized protein that simultaneously regulates biofilm formation, flagellar biosynthesis, pyocyanin production, and iron uptake in *P. aeruginosa*.
• BscR modulates biofilm formation and motility through PipA-dependent transcriptional control.
• PipA disrupts BscR function by interfering with its polar localization in bacterial cells.
• The polar localization of BscR depends on its interaction with CheB.
• The regulatory proteins BscR and PipA exert control over pathogenic potential of *P. aeruginosa*.

## Graphical abstract

**Figure:**
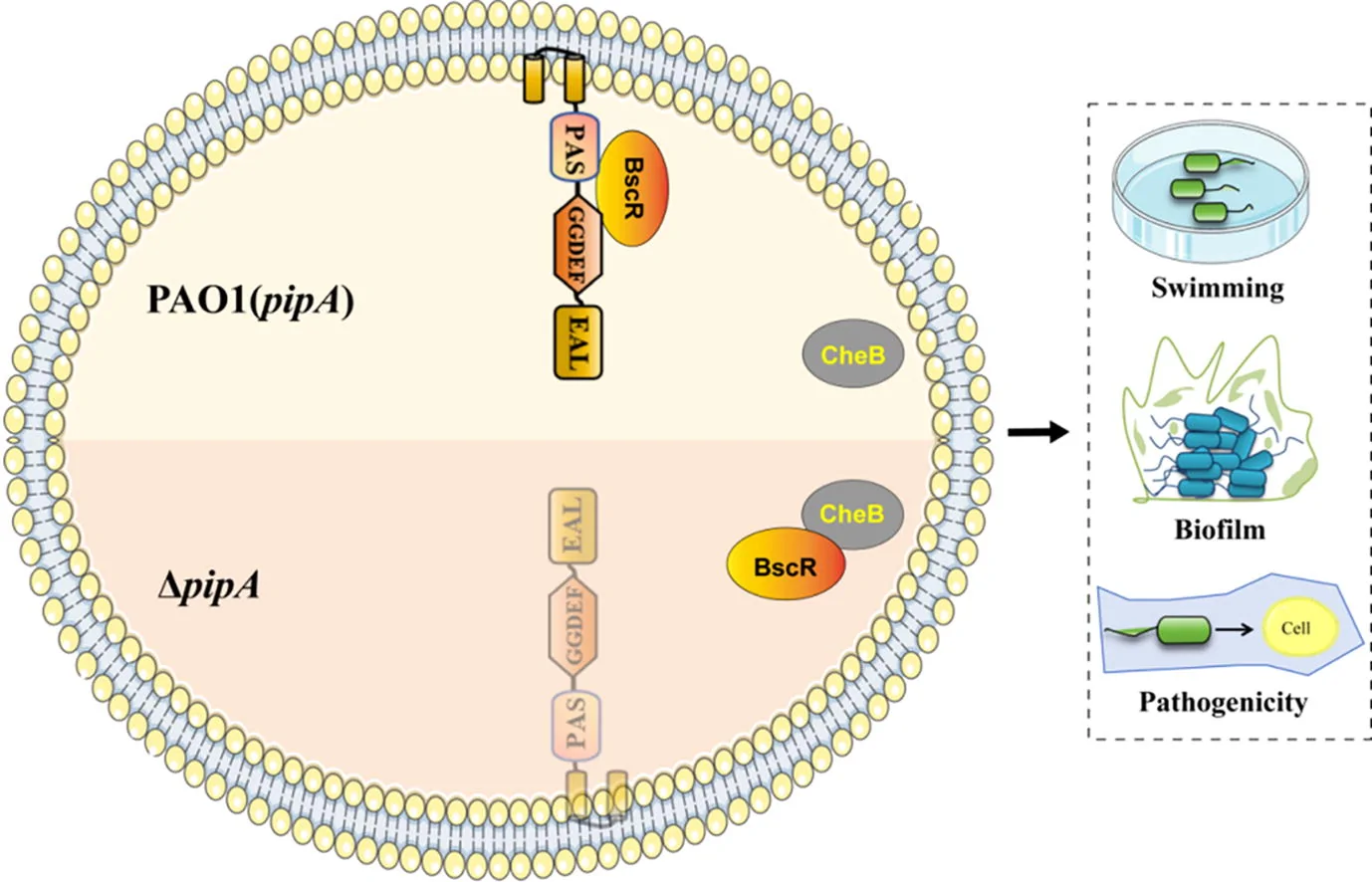

## Introduction

As a Gram-negative opportunistic pathogen, *Pseudomonas aeruginosa* (*P. aeruginosa*) is widely recognized for its role in causing acute and chronic infections, particularly prevalent among immunocompromised patients [1]. *P. aeruginosa* flagellum-mediated motility and expression of the extracellular factor pyocyanin are associated with acute infections [2]. In contrast, during chronic infections, this organism typically forms biofilms composed of substantial matrix components, which form a defensive niche protecting resident bacterial cells from diverse stress factors, including antibiotic challenges [3]. Outbreaks of drug-resistant *P. aeruginosa* infections are prevalent in hospital wards and intensive care units, where this bacterium frequently exhibits resistance to multiple antibiotics, resulting in severe and persistent infections [4]. The regulatory circuitry in *P. aeruginosa* orchestrates critical virulence phenotypes, encompassing cell movement, biofilm architecture, and the biosynthesis of extracellular determinants like pyocyanin, iron-chelating siderophores, and proteolytic enzymes [5]. These mechanisms encompass autoinducer-mediated quorum sensing, two-component signal transduction systems, and cyclic di-GMP signaling pathways [[5], [6], [7]], and they are also subject to epigenetic regulation [8].

Among these regulatory mechanisms, c-di-GMP, a key signaling mediator in Gram-negative bacterial species, has been identified as one of the most prevalent intracellular signaling molecules, regulating various physiological pathways such as biofilm assembly and disassembly, cellular motility, and the expression of virulence factors [9,10]. C-di-GMP levels are precisely regulated via a balance between its synthesis by diguanylate cyclases (DGCs) through GTP condensation, and hydrolysis by phosphodiesterases (PDEs) via phosphodiester bond cleavage [9]. Bioinformatics studies have revealed that DGCs and PDEs are widespread among different types of bacteria. In reality, dozens of DGCs and PDEs can be discovered within one single species, highlighting the complexity and high-precision characteristics of the c-di-GMP regulatory pathway [9,11]. Overexpression of the DGCs SadC and YedQ enhances biofilm formation and strongly inhibits cell motility, whereas the overexpression of PDEs YhjH, DipA, and RbdA produces the opposite effect in *P. aeruginosa* [[12], [13], [14], [15], [16]]. Interestingly, the expression of certain DGCs and PDEs has a minimal impact on the global c-di-GMP pools within the cell. Instead, these DGCs/PDEs are hypothesized to exert specific regulatory roles primarily through physical association with other proteins or modulation of localized c-di-GMP pools.

In canonical c-di-GMP signal transmission mechanisms, the regulatory effect of this secondary messenger is contingent upon its downstream receptors. The N-terminal region of DGCs and PDEs proteins usually possess a sensory domain, which is essential for detecting various environmental signals. This sensing capability subsequently activates the catalytic activities of diguanylate cyclase and phosphodiesterase, thereby modulating intracellular concentrations of c-di-GMP [17]. For instance, in *P. aeruginosa*, the PDE IsmP conformation undergoes a change upon iron sensing, facilitating the release of the DGC IsmA from the IsmP-IsmA complex promoting intracellular c-di-GMP levels, which governs surface attachment and biofilm maturation [18]. Besides canonical c-di-GMP signaling pathways, DGCs and PDEs also influence bacterial physiological behavior through interactions with other proteins. Notably, the two-component regulator CbrB affects the regulation of the PDE RmcA, which in turn modulates the type III secretion system and biofilm formation via direct interaction [19].

The whole genome sequencing analyses have revealed motile bacteria in natural environments frequently harbor multiple operons associated with chemoreceptor systems and signal transduction pathways [20]. These microorganisms exhibit behavioral plasticity by detecting chemical gradients and executing directional motility responses [21]. *P. aeruginosa* exhibits characteristic motility via a singular polar flagellum, a defining feature enabling environmental adaptation [22]. Mechanistically, the polar-localized c-di-GMP receptor MapZ engages with CheR1 methyltransferase within the chemotaxis signaling complex. This interaction suppresses CheR1-mediated methylation, establishing a functional crosstalk between cyclic-di-GMP signaling and chemotactic regulation [23]. Emerging research reveal that spatial organization of CheO in *C. jejuni* requires chemosensory array CheA and CheV/W. Intriguingly, CheO is associated with both chemotaxis proteins CheA and CheZ, as well as flagellar rotor components FliM and FliY, which suggests its dual role in coordinating chemosensory signaling and flagellar motor activity [24].

*P. aeruginosa* encodes over 40 enzymes with GGDEF/EAL domains that collectively orchestrate c-di-GMP homeostasis through synthesis and hydrolysis [10]. Although several of these proteins have been intensively investigated for their enzymatic activities and signaling mechanisms, the regulatory roles of these DGCs/PDEs remain largely unexplored. Our previous work identified a phosphodiesterase PipA, which regulates biofilm formation, cell aggregation, secretion mechanisms and swimming motility [25]. This study sought to investigate the molecular basis of PipA-mediated signaling specificity within *P. aeruginosa* pathways. Through co-immunoprecipitation and bacterial two-hybrid experiments, we identified a putative regulator PA3965, designated as BscR (Biofilm and Swimming and cell poles Regulator), as a binding partner of PipA. Our findings demonstrated that PipA and BscR collaboratively regulate biofilm and motility. Experimental evidence revealed that PipA and CheB are essential for BscR-dependent control of biofilm development and motility behavior. Utilizing molecular docking, we analysed the PipA-BscR and BscR-CheB complexes, identifying the amino acid residues responsible for maintaining their respective interactions. We observed that PipA disrupts the interaction between BscR and CheB, which abolishes BscR's ability to regulate biofilm maturation and swimming behavior.

## Materials and methods

### Bacterial growth, plasmids and cell culture

All *P. aeruginosa* genetic variants, associated plasmids, and oligonucleotide sequences were comprehensively documented in Tables S3 and S4. Standard culturing protocols involved incubation at 37 ℃ in Luria-Bertani (LB) medium, with agar supplementation as required. Bacterial population density was quantified spectrophotometrically using absorbance at 600 nm. A549 cells were propagated in DMEM (Dulbecco's Modified Eagle Medium; Gibco, USA) containing 10 % FBS (fetal bovine serum) and 1 % Pen-Strep (penicillin–streptomycin), under standard culture conditions of 37 ℃ with 5 % CO_2_ in a humidified chamber.

### Generation of mutants and complementation

The in-frame deletion mutants of *P. aeruginosa* were generated via the vector pK18mobsacB [7]. The construction of the *bscR* gene deletion mutant exemplifies our genetic manipulation strategy. This process began with the amplification of upstream and downstream flanking regions of *bscR* through high-fidelity DNA polymerase (Vazyme, China). These fragments were ligated into the pK18mobsacB, generating the recombinant plasmid pK18-*bscR*. The pK18-*bscR* was transferred into wild type using electroporation. After recovery, candidate colonies were streaked on LB medium with 10 % sucrose. Gentamicin susceptibility and sucrose tolerance were used to identify mutant clones, which were subsequently validated via PCR and sequencing analysis.

To complement the mutant strains, the target gene (including its native promoter) was retrieved subsequently ligated into vector pUCP20. The recombinant plasmid was confirmed and transferred into mutant strains via electroporation.

### Bacterial motility assay

The swimming medium consisted of 5 g•L^-1^ peptone (Bacto, USA), 2.5 g•L^-1^ agar (Bacto, USA) and 3 g•L^-1^ yeast extract (Bacto, USA). Bacteria were spot-inoculated onto swimming agar plates. The 2 μl aliquots were directly taken from overnight LB cultures. The swarming medium was prepared using 8 g•L^-1^ nutrient broth, 5 g•L^-1^ Bacto-agar and 5 g•L^-1^ D-glucose [7]. Three replicates per strain were used to measure the maximum motility zone diameter.

### Quantification of bacterial biofilm biomass

Bacterial cultures incubated overnight were resuspended in fresh LB medium and adjusted to a final OD_600_ of 0.002. A 120 μl aliquot of diluted culture was dispensed into a 96-well microwell plate, followed by incubation at 37 ℃ for the designated duration. After carefully aspirating the bacterial suspensions, deionized water was used to rinse the wells thrice to remove non-adherent cells. The biofilm-attached cells were stained with crystal violet solution (0.1 % w/v) for a 15-minute incubation. Subsequently, sterile water was employed to wash away unbound dye thrice. After air-drying, the biofilm mass was quantified by adding 95 % ethanol to solubilize the biofilms. The optical density at 570 nm was subsequently measured [7].

### Pyocyanin measurement test

Following Welsh et al's protocol, pyocyanin was quantified through a colorimetric assay [26]. Briefly, in a 3 mL culture of LB medium, bacterial growth was maintained for 16 h before centrifugation at 13,800 × g. The supernatant was passed through a 0.22 μm filter to eliminate any remaining cellular debris. Absorbance measurements were conducted using a TECAN reader (Switzerland) at a wavelength of 695 nm to quantify the pyocyanin concentration.

### Pyoverdine production assay

As described in a previous study [27], the production of pyoverdine by *P. aeruginosa* in an iron-limited medium was assayed. First, *P. aeruginosa* strains were cultured overnight in LB broth. Subsequently, these cultures were diluted with fresh LB until the OD_600_ reached 0.002. Finally, the diluted cultures were transferred into a 96-well plate with a white base and black walls. The plate underwent incubation at 37°C in a TECAN device. Then, the fluorescence was measured every hour, with an excitation wavelength set at 400 nm and the emission fluorescence detected at 460 nm.

### Real-time quantitative PCR (RT-qPCR)

*P. aeruginosa* strains were incubated in LB broth at 37°C with shaking until the optical density at 600 nm reached 0.8–1.2. Total RNA extraction was performed using Eastep Super Total RNA Extraction Kit (Promega, USA). RT-qPCR was performed with Hifair® C203P1 Multiplex One Step RT-qPCR Probe Kit (Yeasen, China) on an ABI QuantStudioTM1 Flex system (Thermo Fisher Scientific, USA). The primers employed are detailed in Table S4, and the *rplU* gene, a stable reference gene encoding a 50S ribosomal subunit, was utilized to normalize experimental data [28]. Gene expression levels were quantified via the 2^-ΔΔct^ method, with triplicate biological replicates analyzed to calculate mean expression values.

### Transcriptome analysis

The mRNA was size-selected to a range of 200–700 nucleotides via fragmentation and subsequently converted into cDNA. The second-strand cDNA was generated through the action of DNA polymerase I-mediated synthesis, RNase H-mediated RNA degradation, and dNTP. The cDNA fragments were then purified using PCR Purification Kit, subjected to blunt-end repair, and followed by adenylation. Adapter ligation was performed using T4 DNA ligase to prepare libraries for sequencing. We employed agarose gel electrophoresis to conduct size-selection on the ligation products. Subsequently, the samples were sequenced on the DNBSEQ (DNBSEQ Technology) platform. Sequenced reads were processed using the CLC Genomics Workbench, exported as FASTQ files, and aligned against the assembled genome of *P. aeruginosa* PAO1 (BioProject: PRJNA1073193). Gene expression abundance was quantified via Reads per Kilobase of Transcript per Million Mapped Reads (RPKM) to standardize transcriptome profiling. Genes with |log2fold change|≥1.5 were identified as differentially expressed (DEGs) after applying the Benjamini-Hochberg procedure to control FDR ≤ 0.05. Gene Ontology (GO) enrichment analysis was conducted to interpret functional implications of DEGs.

### Microscopic visualization of protein localization in cells

The plasmids pBBR1-MCS5-GFP and pBBR1-MCS5-BscR-GFP were constructed to express GFP and BscR-GFP fusion protein, respectively. To observe the localization of BscR-GFP under PipA expression conditions, the high-copy plasmid pUCP20 was used to express PipA and its mutated forms (PipA^E280A, N328A, E419^, PipA^E280A^ PipA^N328A^, PipA^E419^, named as PipA^m^). The constructed pUCP20-*pipA* and pUCP20-*pipA^m^* were transferred into the PAO1 strain containing pBBR1-MCS5-GFP or pBBR1-MCS5-BscR-GFP, resulting in the expression strain PAO1(*pipA*, BscR-GFP) and PAO1(*pipA^m^*, BscR-GFP). To observe the co-localization of BscR and CheB, a Mini-CTX-Flag plasmid was used to express *pipA* by integrating it into the genome with pUCP20 to drive mCherry (pUCP20-mCherry) and CheB-mCherry (pUCP20-CheB-mCherry) expression. Subsequently, the pBBR1-MCS5-BscR-GFP and pUCP20-CheB-mCherry plasmids were co-introduced into PAO1, Δ*pipA* mutant and PAO1(*pipA*). The engineered bacterial strains were cultured in LB medium supplemented with the necessary antibiotics, allowing growth to reach an OD_600_ of 1.0. Following centrifugation, the cell pellets were immediately transferred onto agarose pads (1 % concentration) for microscopic observation. Fluorescence imaging was performed through Leica LSM900 laser-scanning confocal microscope (Leica Microsystems, Germany).

### TEM (Transmission electron microscopy) ananlysis

The wild-type PAO1 and its genetically modified variant expressing BscR were aerobically cultured in lysogeny broth (LB) at 37 ℃ until achieving an OD_600_ of 1.0. The collected bacteria were first washed with 0.1 M phosphate buffered saline at a pH of 7.2. Subsequently, they were diluted and subjected to staining using a 2 % aqueous phosphotungstic acid solution with a pH of 7.4 for a duration of 20 min. After that, the bacteria were loaded for 5 min on a carbon-coated Parlodion grid (300 mesh). The samples were then observed under a Talos F200S transmission electron microscope (Thermo Fisher, USA) that was operated at 200 kV. To measure the length of the flagella in the obtained image, Image J software was employed.

### Bacterial two-hybrid assay (BTH)

BTH analysis was performed with a λ repressor-based two-hybrid platform (BacterioMatch II System, Agilent Technologies, USA). Target genes (*pipA/bscR*) were directionally subcloned into the bait vector pBT, while *bscR*, *cheB* and candidate genes identified by mass spectrometry sequences were ligated into the pTRG prey vector using Gibson assembly. We performed co-transformation of the pBT-derived and pTRG-derived plasmid constructs into the engineered *E. coli* reporter strain for dual selection. On an M9 plate without histidine, which supplemented with 12.5 μg•ml^−1^ streptomycin and 5 mM 3-AT (3-amino-1,2,4-triazole), the positive colonies were chosen. After that, they were diluted in a gradient manner and then used for plate inoculation. The reporter strain engineered to co-express pBT-LGF2 and pTRG-Gal11P vectors served as the experimental positive control.

### Western blot (WB) analysis

We carried out WB analysis as the procedure detailed in a prior publication [7]. To be specific, we utilized SDS-PAGE to separate the protein samples and then transferred them onto a Polyvinylidene Fluoride (PVDF) membrane (Merck Millipore, USA). The membrane was incubated in 5 % (w/v) skim milk powder dissolved in TBST (Sangon, China) prior to immunoblotting using anti-His or anti-Flag antibodies (Abbkine, USA) and HRP-labeled goat anti-mouse IgG (Abbkine, USA). Membrane-bound proteins were visualized via an ECL Plus HRP Substrate Kit (Abbkine, USA), and subsequently scanned with a cooled CCD scanner (Tanon, China).

#### Pull down assay

We cloned the entire *pipA* sequence into pET-28a protein expression vector and transformed the recombinant vector into *E. coli* BL21 (DE3). After overnight growth, the culture was treated with 0.5 mM IPTG to initiate expression of PipA. The PipA protein was enriched using Nickel (Ni) beads (Smart, China). The pUCP20 expression plasmid was used to express BscR with a Flag tag. The resulting pUCP20-BscR-Flag plasmid was transferred into wild-type PAO1, and the bacterial pellet was lysed by sonication to release BscR-Flag. After treating 100 μg of purified PipA with Ni-NTA agarose resin at 4 °C for 4 h, unbound proteins were washed away via centrifugation. Subsequently, the cell extract harboring BscR-Flag protein was introduced to trigger protein interaction. Following overnight incubation, the resin-bound complexes were eluted and subjected to immunoblot analysis. To confirm the interaction between BscR and CheB, a pUCP20-CheB-His plasmid was generated using a similar method.

#### c-di-GMP determination

PipA protein was mixed with c-di-GMP (50 µM, Sigma-Aldrich, Germany) in a buffer solution containing Tris-HCl (60 mM, pH 7.5), DDM (0.025 %), MgCl_2_ (10 mM) and EDTA (1 mM). The reaction mixture was incubated at 37 °C for 60 min and subsequently heat-inactivated by boiling for 10 min. Chromatographic analysis was conducted on an Agilent Infinity II HPLC system, employing a C_18_ column under the following conditions: flow rate of 0.3 mL•min^−1^, injection volume of 2.5 µL. An isocratic mobile phase consisting of methanol and 1 % (w/v) ammonium acetate in water was used at a ratio of 30:70 (v/v).

Using the Cyclic-di-GMP Assay Kit (Catalog Number: 200–100) from Lucerna Technologies (USA), we performed quantification of c-di-GMP concentrations in bacterial cells according to the instruction manual.

### Modelling for BscR_2_-PipA_2_-BscR_2_ and BscR_2_-CheB_2_

Predicted models for full-length PipA and BscR, as well as BscR and CheB, were retrieved from AlphaFold (https://alphafoldserver.com/welcome) Protein Structure Database. To investigate potential intermolecular associations, the PDBsum web interface (accessible via EBI resources) was employed for protein interaction profiling, with structural analyses performed. The structural integrity of the PipA-BscR hetero-hexamer candidate was confirmed through computational analysis using the EBI Protein, Interfaces, Surfaces and Assemblies (PISA) server.

### Construction of bacterial strains for dual-gene expression

For the cytotoxicity assays involving simultaneous expression of PipA (or its mutants) and BscR, the following sequential transformation procedure was used. First, pUCP20-derived plasmids expressing PipA, PipA^E280A^, PipA^N328A^, or PipA^E419A^ (each with a C-terminal His tag) were introduced into *P. aeruginosa* PAO1 via electroporation, and transformants were selected on LB agar containing carbenicillin (150 µg/mL). Subsequently, pBBR1-MCS5-BscR-Flag (expressing BscR with a C-terminal Flag tag) was introduced into these strains by triparental mating using the helper plasmid RK600, followed by selection on LB agar containing both carbenicillin (150 µg/mL) and gentamicin (60 µg/mL). The co-expression of PipA (or its mutants) and BscR in the resulting strains was confirmed by Western blot analysis using anti-His and anti-Flag antibodies (as described in the Western blot analysis section).

### Cell death assay

Prior to treatment, A549 cells were cultured in 96-well flat-bottom plates containing DMEM/high glucose medium enriched with 10 % heat-inactivated FBS (Gibco, USA). Cells were maintained in a humidified atmosphere (5 % CO_2_, 37°C) for overnight incubation (16–18 h), achieving 80–90 % surface coverage as quantified by phase-contrast microscopy. Following removal of the culture supernatant, the cell monolayer was rinsed once with phosphate-buffered saline. Fresh bacterial cultures were diluted in DMEM medium, and a 100 μL aliquot of the suspension was applied to the A549 cell monolayer, with a multiplicity of infection (MOI) set at 50. Then, a 4-hour incubation, the supernatants from cell cultures were harvested for LDH activity quantification, which was performed using a commercially available cytotoxicity detection kit (Catalog Number: 40209ES76, Yeasen Bio, China) following the manufacturer's protocol. Apoptosis or necrosis can release LDH from the cytoplasm of cells. By measuring the activity of LDH released into the culture medium, quantitative analysis of cytotoxicity can be achieved.

### *Galleria mellonella* killing

As previously reported in reference [29], the protocol for performing killing assays using *G. mellonella* (wax moth) larvae was followed. Specifically, *P. aeruginosa* strains were cultivated until their OD_600_ measured 0.8. Then, the strains were collected and washed by sterile PBS. A bacterial dilution of 10 μL (approximately 10^5^ bacteria) was injected into *G*. *mellonella* by syringe, followed by incubation for 48 h at 30°C. A 10 μL volume of sterile PBS was administered to the negative control group via injection. Larvae exhibiting no reaction to physical prodding of the head region were recorded as nonviable.

### Cellular ROS production measurement

The Reactive Oxygen Species Assay Kit (Beyotime, China) was used to measure the levels of reactive oxygen species (ROS) produced in A549 cells. *P. aeruginosa* strains were used to infect A549 cells at an MOI of 50. After a 4-hour post-infection period, cells were rinsed thrice with PBS and incubated in DMEM supplemented with 10 μM DCFH-DA (2′-7′ dichlorofluorescin diacetate) for 20 min at 37°C. Following a third wash with PBS, the cells were collected. Fluorescently labeled cells were examined by a CytoFLEX S flow cytometer (Beckman Coulter, Inc., USA), employing 488 nm laser excitation and 525 nm emission.

### Statistical analysis

All experiments were performed with at least three independent biological replicates unless otherwise specified. Data are presented as mean ± standard deviation (SD). Statistical analyses were performed using GraphPad Prism (version 8.0.0). For comparisons between two groups, a two-tailed, unpaired Student’s *t*-test was used. For comparisons among more than two groups, a one-way analysis of variance (ANOVA) was performed. Specific statistical tests used for each experiment are indicated in the corresponding figure legends. A *P* value of < 0.05 was considered statistically significant.

## Results

### BscR regulates multiple virulence traits in *P. aeruginosa*

**Fig. 1:**
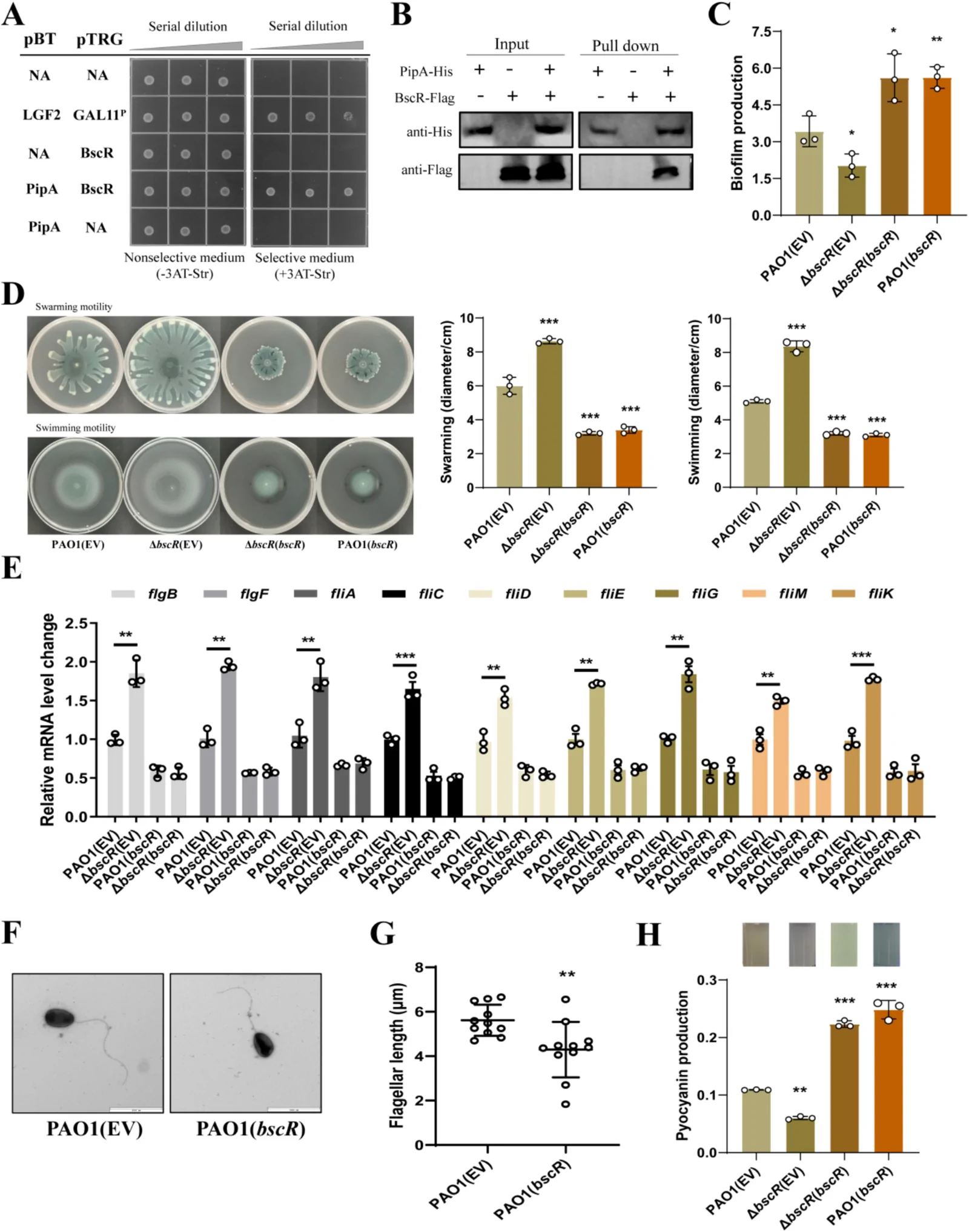
**BscR regulates multiple phenotypes of *P. aeruginosa*.** (A) BTH assay revealed an interaction between PipA and BscR. NA represents the empty vectors pBT or pTRG. Growth in dual-selective media confirms the protein–protein interaction. Positive control includes strains expressing LGF2 and Gal11^P^. (B) Pull-down assay verified the interaction between PipA and BscR, where BscR with 3 × Flag-tag was released from PAO1 cell lysates and incubated with PipA-His on Ni beads, followed by elution and detection via Western blotting. (C) Deletion of *bscR* attenuates biofilm development. Biofilm mass was quantified in wild-type PAO1, *bscR* deletion mutant and its complementation Δ*bscR*(*bscR*), as well as the strain PAO1(*bscR*) where *bscR* was expressed in PAO1 by OD_570_ measurement. (D) The BscR negatively regulates swarming and swimming motility. The swarming and swimming behaviors of the tested strains were measured (left panel) in a semisolid medium. Representative images are shown. Diameters of swarming motility and swimming motility for each strain were quantified and were presented in the middle and right panels, respectively. (E) Measurement of flagella-related gene mRNA levels in strain PAO1, *bscR* deletion mutant and its complementation Δ*bscR*(*bscR*), as well as strain PAO1(*bscR*), suggesting that BscR regulates the expression of those genes. (F) TEM images showed flagellum in wild-type PAO1 and PAO1(*bscR*) strains. A typical image for each strain was presented. (G) The average flagellum length was determined for the strains in panel (F), with eleven cells measured per strain. (H) Pyocyanin production of the indicated strains was measured. The culture image and pyocyanin production of each strain was displayed (up panel) and quantified (down panel), respectively. Data are presented as mean ± SD (11 cells per strain from three independent experiments in panel G and three independent experiments in panels C, D, E, and H). *, *P* < 0.05; **, *P* < 0.01; ***, *P* < 0.001 *versus* PAO1(EV) based on unpaired Student’s *t* test (E) or *versus* PAO1(EV) based on one-way ANOVA (C, D, G, H). EV, empty vector for the control.

Our previous study demonstrated that the phosphodiesterase PipA in *P. aeruginosa* regulates swimming behavior and biofilm dispersion at the transcriptional level [25]. To explore the underlying mechanism of its activity, we hypothesized that PipA regulates biofilm and swimming via interactions with specific partners as part of the c-di-GMP regulatory network. We performed co-immunoprecipitation experiment coupled with protein sequencing (CoIP-seq) to identify PipA partner proteins in *P. aeruginosa*. For this purpose, we expressed PipA-GFP in bacteria, and then enriched for PipA-partner protein complexes using GFP-affinity beads. Among the candidates identified by mass spectrometry analysis, we prioritized PA3965 (designated BscR) for in-depth validation. Bioinformatic analysis predicted BscR to be a transcriptional regulator of the asparagine synthase C (AsnC) family, harboring a conserved DNA-binding domain (Table S1). Given that PipA modulates phenotypes at the transcriptional level [25], we reasoned that its interaction with a transcription factor could represent a direct mechanism for modulating downstream gene expression. To validate this interaction, we performed a BTH with the entire PipA as the bait to screen potential interacting protein. Robust growth on selective medium further corroborated that BscR is indeed a binding partner of PipA (Fig. 1A). Finally, we confirmed the PipA-BscR interaction through *in vitro* pull-down assay, where purified PipA-His effectively interacted with BscR-Flag (Fig. 1B). These results substantiate that BscR acts as a binding partner of PipA. To assess the specificity of our screen, we also tested seven additional candidate interactors via BTH. Five (PA3309, PA4723, PA0937, PA2562, PA5308) showed positive interactions, while two (PA3196, PA1899) did not yield significant signals under these conditions, possibly due to weak or transient interactions that were not stably captured under the experimental conditions (Fig. S1). However, the strong phenotypic correlation observed upon *bscR* deletion (detailed below) and its predicted central regulatory role focused our subsequent mechanistic investigation on the PipA-BscR regulatory axis.

Since PipA modulates both biofilm development and bacterial motility [25] and interacts with BscR, we hypothesized that BscR also modulates PipA-associated phenotypes. To this end, a *bscR* deletion mutant was constructed in PAO1 parental strain background. We subsequently generated complementation, designated as Δ*bscR*(*bscR*), and an expression strain, designated as PAO1(*bscR*), by expressing *bscR* in the *bscR* deletion mutant and wild-type PAO1, respectively. As anticipated, the biofilm formed by the Δ*bscR* mutant was notably less than that of the wild-type PAO1 after an 8-hours incubation, while expression of *bscR* enhanced biofilm formation in both Δ*bscR* mutant and wild-type PAO1 contexts (Fig. 1C). Next, we investigated whether BscR influences motility. Our results indicated that deletion of *bscR* resulted in larger zones of swarming and swimming, suggesting that BscR negatively regulates both swarming and swimming motility (Fig. 1D). Pel polysaccharide, acting as a principal structural element, is integral to the matrix formation of *P. aeruginosa* biofilm [30]. Therefore, the mRNA levels of *pelA*-*C* were measured in our engineered strains. The Δ*bscR* mutant exhibited downregulated pelA-C mRNA expression relative to PAO1, which suggested that BscR regulates biofilm formation through its influence on Pel exopolysaccharide production (Fig. S2). Growth curves for the Δ*bscR* mutant confirmed that these observed biofilm and motility phenotypes were not attributable to changes in growth rates (Fig. S3).

Given that swarming and swimming motility are mediated by the flagellum [31], we investigated flagellum biosynthesis in the Δ*bscR* mutant. We initially assessed the transcriptional levels of genes associated with flagellar biosynthesis in wild type strain and its derivatives. RT-qPCR data indicated that BscR has an obvious effect on the expression of these genes (Fig. 1E). Subsequently, we examined the flagella of both wild-type PAO1 and the PAO1(*bscR*) strain using TEM, since expression of *bscR* in PAO1 significantly repressed swarming and swimming motility (Fig. 1D). We observed a significant reduction in flagellum length in the PAO1(*bscR*) mutant compared to the wild-type strain (Fig. 1F and G).

During the cultivation process, we noticed that the culture of the PAO1(*bscR*) strain exhibited a greener appearance compared to both PAO1 and Δ*bscR* mutant strains (Fig. 1H). This green pigment prompted us to investigate whether BscR regulates the pyocyanin production, which is a metabolite known to suppress microbial competitors' proliferation, giving *P. aeruginosa* a competitive advantage [32]. Indeed, we observed that production of pyocyanin in the Δ*bscR* was decreased relative to PAO1, and expression of *bscR* in the Δ*bscR* mutant and PAO1 strains significantly increased pyocyanin production (Fig. 1H). Finally, we measured the mRNA levels of genes related to pyocyanin biosynthesis in the indicated strains and found that BscR positively regulates the expression of these genes (Fig. S4). Collectively, these discoveries show that BscR acts as a regulator for different virulence properties in *P. aeruginosa*.

### BscR modulates both bacterial iron uptake and cell polar distribution

**Fig. 2:**
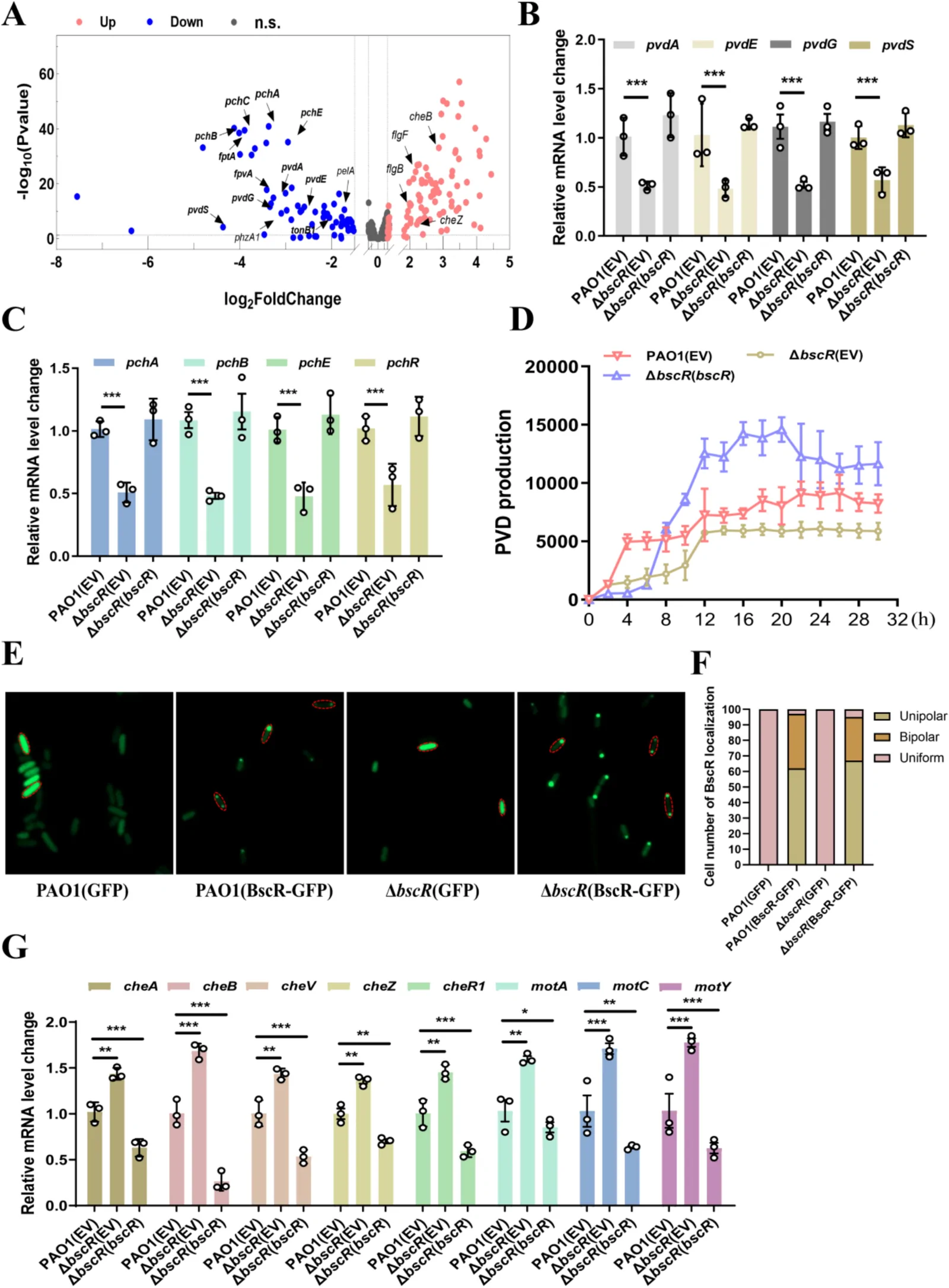
**BscR controls iron uptake and distributed at the cell poles.** (A) The volcano plot visualizes DEGs between the Δ*bscR* mutant and PAO1, with siderophore-related genes highlighted. Up: upregulation; Down: downregulation; n.s.: not significant. (B and C) Expression of pyoverdine biosynthesis genes (B) and pyochelin biosynthesis genes (C) was detected in strain PAO1, the Δ*bscR* mutant, and its complementation by RT-qPCR. (D) Pyoverdine production was quantified in strain PAO1, the Δ*bscR* mutant as well as its complementation. (E) Localization of GFP and BscR-GFP in PAO1 and Δ*bscR* mutant were observed by fluorescence microscopy. (F) The polar localization distribution of GFP and BscR-GFP was quantified in PAO1 and Δ*bscR* mutant cells (100 cells per strain from three independent experiments). (G) We quantified chemotaxis-related gene expression via RT-qPCR in PAO1 strain, Δ*bscR* mutant and its complementation. Data are presented as mean ± SD (three independent experiments). *, *P* < 0.05; **, *P* < 0.01; ***, *P* < 0.001 *versus* PAO1(EV) based on unpaired Student’s *t* test. EV, empty vector for the control.

To comprehensively explore the regulatory role of BscR in *P. aeruginosa*, the RNA-Seq was conducted to compare transcriptomes of wild-type PAO1 and Δ*bscR* mutant. Our analysis of DEGs (differentially expressed genes) indicated a total of 174 genes with an absolute log2fold change of ≥ 1.5 between the two strains. Specifically, when comparing the Δ*bscR* mutant with the wild-type PAO1, 95 genes showed up-regulation, while 79 genes exhibited down-regulation (Fig. 2A, Table S2). We conducted the DEGs enrichment analysis based on Gene Ontology (GO) terms, which indicated that these genes were related to a series of biological functions, for example, iron response, iron uptake, iron regulation, and etc (Fig. S5). Notably, the process by which *P. aeruginosa* acquires iron is intricate, primarily involving two siderophores: pyoverdine and pyochelin, along with the transport protein TonB [33]. Indeed, we noticed that the most abundant genes regulated by BscR were involved in the biosynthesis of pyoverdine and pyochelin, as well as the TonB transporter (Fig. 2A). Gene expression of the pyoverdine (*pvdA*, *pvdB*, *pvdE*, *pvdS*) and pyochelin (*pchA*, *pchB*, *pchE*, *pchR*) biosynthetic pathways were quantified via RT-qPCR [33]. The results indicated that, when compared with those in the wild-type PAO1, the transcriptional levels of these chosen genes were notably decreased in the Δ*bscR* mutant (Fig. 2B and C). Introduction of *bscR* into the Δ*bscR* mutant rescued the transcriptional activity of the those genes to levels comparable to the wild-type strain (Fig. 2B and C). Additionally, we assessed pyoverdine production in PAO1, Δ*bscR* mutant, and its complementation strain. We observed a decrease in pyoverdine biosynthesis in the Δ*bscR* mutant, however, *trans*-complementation with *bscR* successfully reestablished production levels equivalent to the parental strain (Fig. 2D).

Since BscR regulates flagellar biosynthesis (Fig. 1E), and given that key flagellar structural/assembly components (e.g., FlhF) are known to localize at the cell poles to coordinate motility [34], we hypothesized that BscR might also exhibit polar localization to spatially organize its regulatory function. We constructed a fusion of BscR with a green fluorescent protein (GFP) at its C-terminal in PAO1 and Δ*bscR* mutant cells to investigate the cellular localization of BscR. Fluorescence microscopy analysis revealed uniform GFP signal distribution across both PAO1 and Δ*bscR* mutant cells (Fig. 2E). Conversely, the GFP-tagged fusion protein BscR localized at the cell poles (Fig. 2E). We observed that the BscR distribution within the cells is uneven, with a greater prevalence of localization in unipolar stages (Fig. 2F). To confirm whether the fusion with GFP affected the functionality of BscR, we assessed swimming behavior. The data displayed that the PAO1 strain expressing a BscR-GFP fusion gene exhibited swimming motility comparable to that of the *bscR*-expressing PAO1 strain (Fig. S6), indicating that the fusion did not impair BscR function. Previous studies have demonstrated that some chemotactic proteins, such as CheA and CheY, influence the movement of bacteria on swimming plates, with these proteins predominantly clustering at polar regions of *P. aeruginosa* cells [35]. We speculate that BscR may be a chemotaxis-related regulatory factor in bacteria. For this purpose, we investigated the transcriptional levels of selected chemotaxis-associated genes in indicated strains. The results revealed that targeted chemotaxis genes exhibited significant upregulation of in the Δ*bscR* mutant relative to PAO1 control, while these levels were restored in the complemented Δ*bscR* strain (Fig. 2G). Collectively, the results elucidate that BscR affects the synthesis of bacterial siderophore and is distributed at the cellular poles.

### BscR is involved in regulating swimming motility and biofilm formation by PipA

**Fig. 3:**
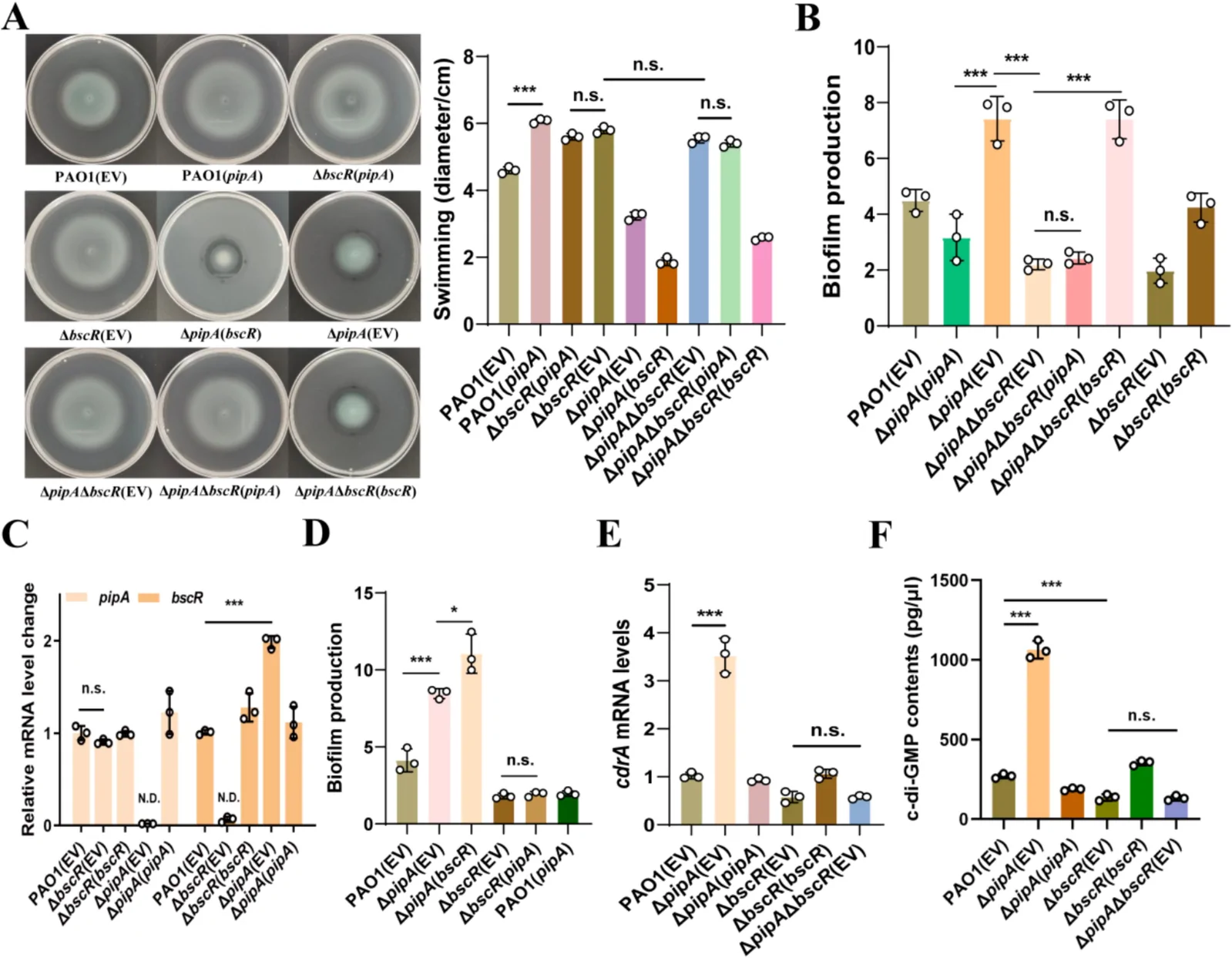
**PipA regulates biofilm formation and swimming behavior through BscR.** (A) Left panel: Representative images of swimming motility. Right panel: Quantification of swimming zone diameters. Strains analyzed include: wild-type PAO1, isogenic mutants (Δ*bscR*, Δ*pipA*, and Δ*pipA*Δ*bscR*), and complemented strains. For *pipA* complementation: PAO1(*pipA*), ΔbscR(*pipA*), and Δ*pipA*Δ*bscR*(*pipA*). For *bscR* complementation: Δ*pipA*(*bscR*) and Δ*pipA*Δ*bscR*(*bscR*). (B) Biofilm production of the indicated strains was assessed using crystal violet staining, with subsequent measurement of absorbance at OD_570_. (C) Expression levels of *pipA* and *bscR* were measured in the indicated strains using RT-qPCR. (D) Biofilm production was assessed in wild-type PAO1, Δ*pipA*, Δ*bscR*, Δ*pipA*(*bscR*) and Δ*bscR*(*pipA*) strains. (E) Expression of *cdrA* were assessed in PAO1, Δ*pipA*, Δ*bscR* and their complementations as well as Δ*pipA*Δ*bscR* to infer cellular c-di-GMP concentrations. (F) Cellular c-di-GMP levels of indicated strains was measured using a commercial C-di-GMP Assay Kit. Error bars represent the means ± SD from triplicate assays. *, *P* < 0.05; ***, *P* < 0.001 *versus* the indicated group based on unpaired Student’s *t* test. n.s., not significant; N.D., not detected; EV, empty vector for the control.

Integrative analysis of virulence determinants demonstrated BscR's central regulatory function in modulating biofilm production and swimming motility, while PipA also influenced these two virulence-associated traits. Moreover, our findings indicated a direct interaction between BscR and PipA. Based on this evidence, we hypothesized that PipA might have regulated biofilm formation and swimming motility through its interaction with BscR. To this end, we expressed *pipA* in both strain PAO1 and the Δ*bscR* mutant, as well as expressed *bscR* in the Δ*pipA* mutant. The swimming behavior of the Δ*pipA* mutant was diminished upon the expression of *bscR*, whereas expression of *pipA* in the Δ*bscR* mutant largely counteracted the promoting effect of PipA on swimming behavior (Fig. 3A). Furthermore, we generated a double mutant, Δ*pipA*Δ*bscR*, by introducing a *bscR* mutation into the *pipA* deletion mutant background. As expected, the Δ*pipA* mutant formed biofilm to a significantly greater extent than the wild-type PAO1 strain. The phenotypic comparison between Δ*pipA*Δ*bscR* and Δ*bscR* mutants revealed no significant differences in biofilm mass and swimming motility (Fig. 3A and B). The enhanced biofilm formation and reduced swimming motility observed in the Δ*pipA* mutant were completely abolished upon deletion of *bscR* (Fig. 3A and B). Complementation of the Δ*pipA*Δ*bscR* double mutant with *bscR* restored both biofilm production and swimming behavior. In contrast, complementation with *pipA* did not alter the phenotype of the double mutant (Fig. 3A and B). These findings demonstrate that an antagonistic relationship between PipA and BscR in modulating biofilm development and bacterial motility, suggesting the existence of a potential regulatory cascade between these two components.

To investigate the purpose further, we quantified the mRNA levels of *bscR* in the Δ*pipA* and the *pipA* in the Δ*bscR* mutant, using RT-qPCR. Our results indicated that *bscR* does not influence the expression of *pipA*, however, *pipA* negatively regulates the expression of *bscR* (Fig. 3C). We then constructed a strain Δ*pipA*(*bscR*), in which the *bscR* gene was expressed in the Δ*pipA* mutant and assessed the biofilm production of this strain. The results demonstrated that the Δ*pipA*(*bscR*) strain exhibited enhanced biofilm formation compared to the Δ*pipA* mutant, while *pipA* failed to reduce biofilm production in the *bscR* deletion mutant (Fig. 3D). These findings provide additional evidence confirming the regulatory role of *pipA* in controlling *bscR* expression. To evaluate the c-di-GMP production in the Δ*pipA* and Δ*bscR* mutant cells, we analyzed the mRNA levels of *cdrA*, a gene serving as a c-di-GMP-responsive marker, using RT-qPCR [36]. The data displayed that the deletion of *pipA* led to increased expression of *cdrA*, a finding consistent with a previous study [25]. The mutation of *bscR* results in reduced *cdrA* expression, suggesting that BscR positively controls the intracellular c-di-GMP biosynthesis (Fig. 3E). Additionally, no significant difference in *cdrA* mRNA levels was observed between Δ*bscR* mutant and Δ*pipA*Δ*bscR* double mutant (Fig. 3E). Intracellular c-di-GMP levels were further measured by a commercial assay kit, and the results were consistent with those indicated by *crdA* (Fig. 3F). Overall, these results supported that BscR participated in the PipA signaling pathway, which regulated swimming motility and biofilm formation.

### PipA affects BscR function through a physical interaction

**Fig. 4:**
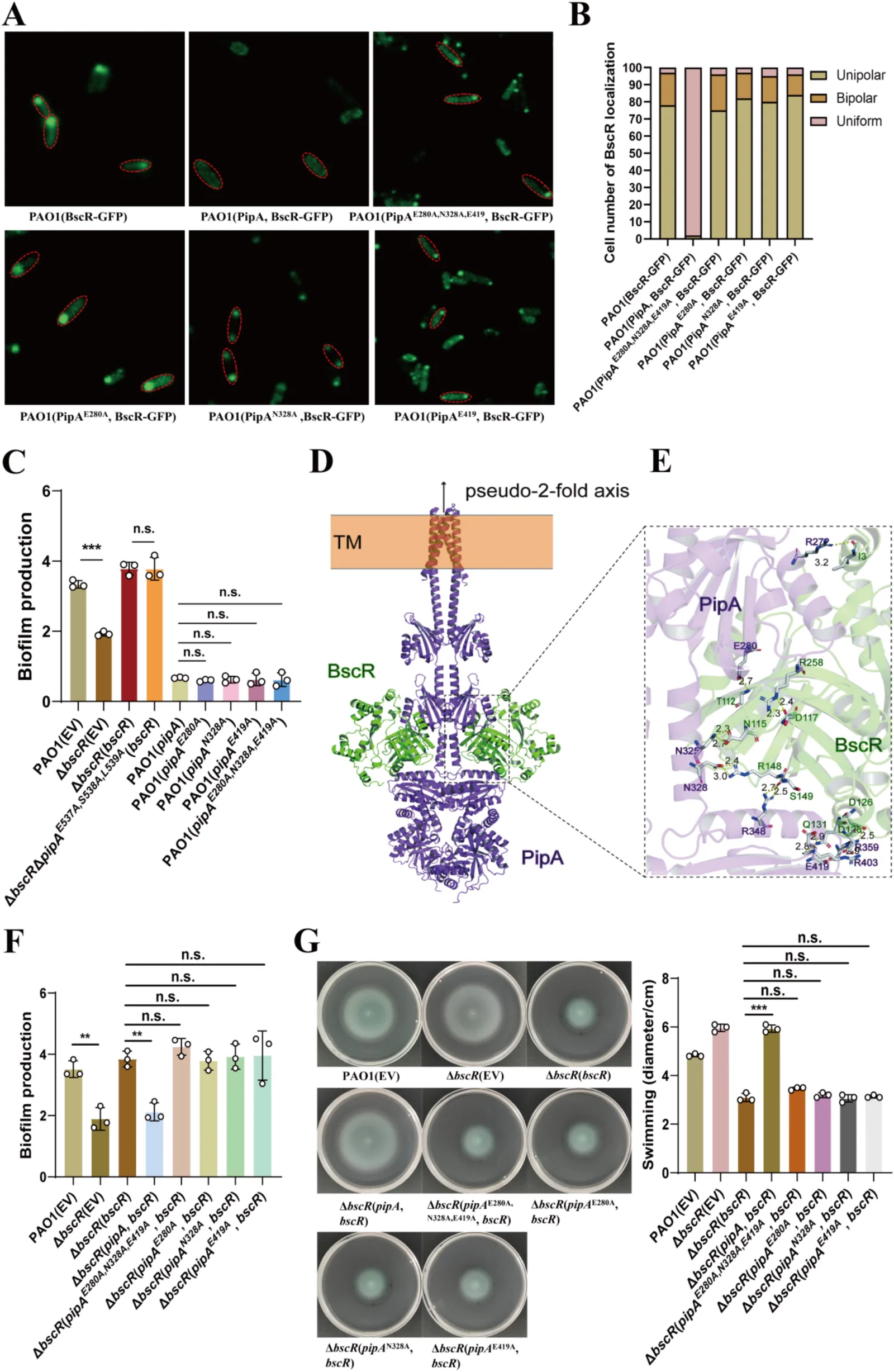
**PipA inhibits BscR activity via a physical interaction.** (A) Cellular localization of BscR-GFP was examined in PAO1, PAO1(*pipA*) and in PAO1 expressing amino acid mutations of PipA. (B) The polar localization distribution of BscR-GFP was quantified in strain PAO1, PAO1(*pipA*), and PAO1-derived strains expressing amino acid mutation of PipA (100 cells per strain from three independent experiments). (C) Biofilm production of indicated strains were measured to indicate PDE activity of PipA. (D) A potential model for the PipA-BscR interaction was developed through molecular docking. (E) Details of the PipA-BscR heterodimer interface are presented. (F) The PipA-BscR interaction model was validated through quantitative analysis of biofilm production in the tested bacterial strains via OD_570_ measurement. (G) Swimming behavior of the tested strains were monitored in a petri dish (left panel) and quantified via measurement of swimming zone diameters (right panel). Representative images are shown. Data are presented as mean ± SD (three independent experiments). **, *P* < 0.01; ***, *P* < 0.001 *versus* the indicated group based on unpaired Student’s *t* test. n.s., not significant; EV, empty vector for the control.

Recent studies have shown that disruption of WspR cellular polarization in *P. aeruginosa* abolished its regulatory effects on biofilm formation and motility [37]. Interestingly, we observed that BscR-GFP exhibited a non-polar distribution across the cells when PipA was expressed in the PAO1 strain (Fig. 4A and B). The *pipA* gene was expressed in the PAO1(*bscR*) strain, lifting the suppression of swimming motility and the promotion of biofilm production regulated by BscR (Fig. S7). Given the identified interaction between PipA and BscR, we hypothesized that an excess of PipA influences the localization of BscR through a physical interaction. This effect may further disrupt BscR-regulated swimming motility and biofilm production. To determine whether BscR affects the PDE activity of PipA through binding to PipA, we introduced a mutation from ESL to AAA (alanine) at the PDE activity site of PipA in the *b*sc*R*-mutant complemented genetic background. Those findings suggested that the ESL mutation did not alter the ability of BscR to regulate biofilm formation, suggesting that BscR does not regulate the PDE activity of PipA (Fig. 4C).

We utilized molecular docking combined with simple molecular dynamics simulations based on the outputs from the AlphaFold program to identify potential structural interaction models between PipA and BscR [38]. The results showed that the PipA-BscR complex could form a hetero-hexamer (BscR_2_-PipA_2_-BscR_2_), with the predicted hetero-dimer interface highlighting a strong interaction between PipA and BscR (Fig. 4D, Fig. S8). PISA analysis revealed a buried surface area of 6785.5 Å^2^ at the PipA-BscR binding interface, with binding free energy calculations (Δ^i^G = -69.3 kcal•mol^−1^) revealing predominant hydrophobic stabilization. In addition, a pseudo-dyad axis was observed at the interface. Specifically, the R359 residue within PipA's chain A established polar interactions with BscR's chain B residues D126 and D128, mediated by hydrogen bond formation. This interaction is equivalent to how R359 in chain B of PipA interacts with D126 and D128 in chain A of BscR. Notably, residues E280, N328, and E419 in PipA are capable of forming hydrogen bonds and/or salt bridges with T112, R148, and Q131 in BscR, respectively (Fig. 4E). Moreover, residues R272, N325, R348, R359, and R403 in PipA contribute to the interface by forming polar bonds with I3, N115, S149, D126/D128, and Q131 in BscR, respectively (Fig. 4E).

To validate this interaction model, we mutated the residues E280, N328 and E419 in PipA to alanine (A), either individually or in combination. We then conducted the BTH assay to evaluate how these mutations affected the interaction between PipA and BscR. The results indicated that the interaction between BscR and PipA was lost when residues E280, N328, and E419 were mutated, whether individually or together, demonstrating the reliability of our interaction model (Fig. S9A). Subsequent experiments assessed whether mutations at residues 280, 328, and 419 in PipA disrupted its ability to regulate BscR's intracellular localization. The data exhibited that the expression of the four amino acid variants of PipA did not alter the cellular localization of BscR compared to wild-type PipA (Fig. 4A). These results confirmed the critical role of key residues and the existence of a protein–protein interaction interface. Furthermore, biofilm mass and swimming motility assays showed that expression of PipA led to a loss of BscR's regulatory effects on swimming behavior and biofilm development in the complementation of *bscR* deletion mutant. However, the expression of the four amino acid-mutated PipA did not affect BscR-regulated biofilm formation and swimming behavior (Fig. 4F and G). We found that the mutations at the amino acids positions 280, 328, and 419 of PipA did not affect its capacity to reduce biofilm formation (Fig. 4C). Moreover, the protein expression levels of the four mutated PipA variants remained consistent with those of the wild-type PipA (Fig. S10A). These results suggest that these mutations do not disrupt PipA function. Taken together, these findings indicated that PipA interacts with BscR, leading to the mislocalization of BscR within the cell and subsequently disrupting its function, and proposing a possible hetero-hexamer model for the PipA-BscR complex, which elucidates the structural basis by which PipA affects the function and localization of BscR.

### Polar localization of BscR depends on chemosensory component CheB

**Fig. 5:**
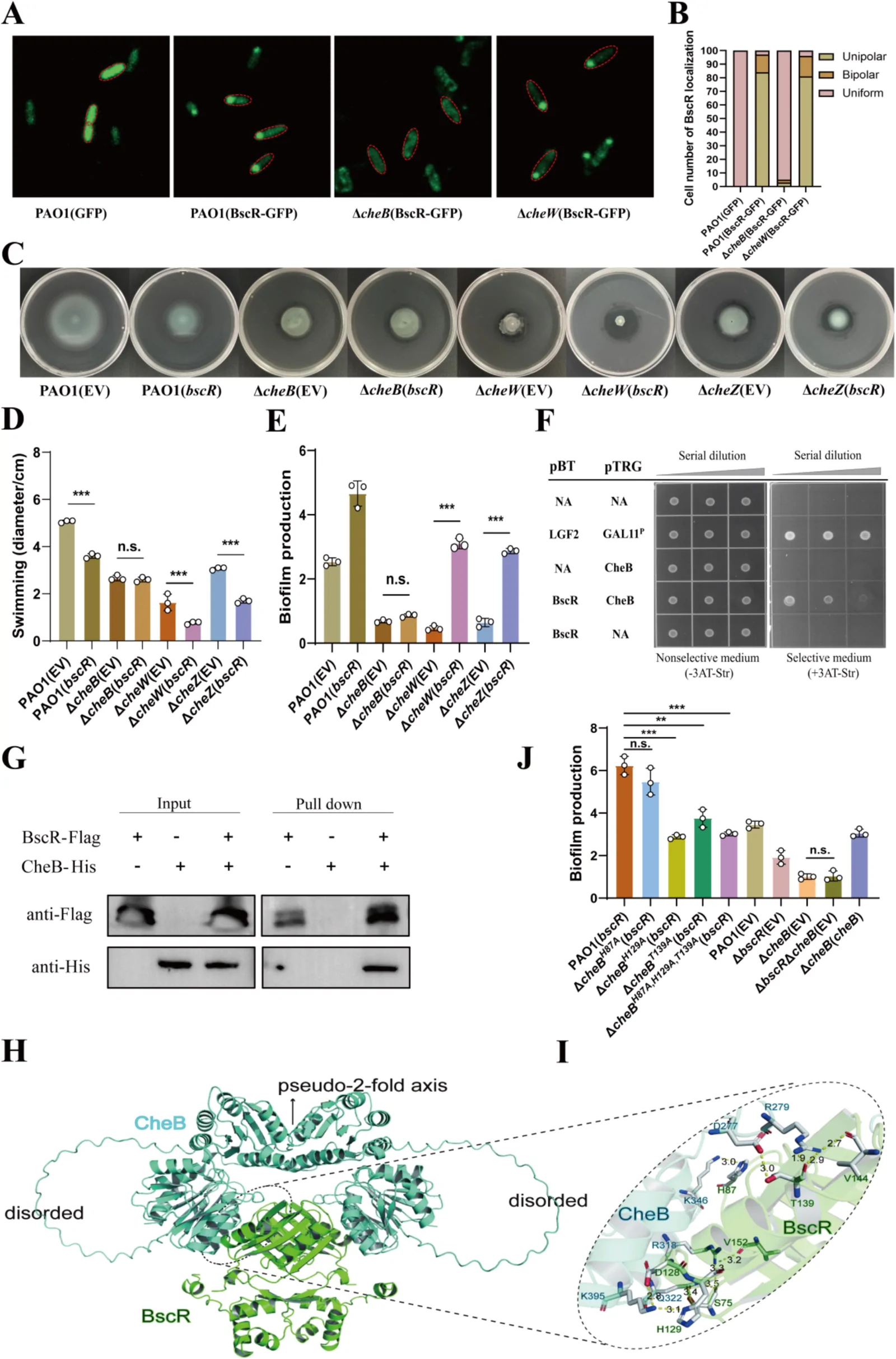
**Polar localization of BscR depends on the presence of CheB.** (A) Subcellular distribution analysis of BscR-GFP in PAO1 strain, Δ*cheB*, and Δ*cheW* mutant strains, compared with GFP localization in PAO1 strain. (B) The polar localization distribution of GFP and BscR-GFP was quantified in strain PAO1, Δ*cheB* and Δ*cheW* mutants cells (100 cells per strain from three independent experiments). (C) Representative images of swimming motility are shown for strain PAO1, Δ*cheB* mutant, Δ*cheW* mutant and Δ*cheZ* mutant, as well as for strains Δ*cheB*(*bscR*), Δ*cheW*(*bscR*) and Δ*cheZ*(*bscR*), in which *bscR* was expressed in Δ*cheB*, Δ*cheW* and Δ*cheZ* mutants, respectively. (D) Swimming diameters of the panel C were determined. (E) Biofilm productions of the indicated strains were measured. (F) An interaction between BscR and CheB was detected using a BTH. NA represents the empty vectors pBT or pTRG. Growth in dual-selective media indicates a protein–protein interaction. The LGF2 and Gal11^P^ −expressing straing served as positive control. (G) The interaction between BscR and CheB was validated through a pull-down assay. BscR tagged with 3 × Flag and CheB-His were extracted from cell lysates of PAO1, followed by co-incubation with anti-Flag magnetic beads. The captured protein complexes were subsequently eluted and subjected to Western blot analysis for detection. (H) Overview of the BscR-CheB_2_-BscR hetero-tetramer complex analyzed by molecular docking. (I) Residue sidechains located within the heterodimer interface are depicted using a ball-and-stick representation, where each atom is color-coded according to its elemental identity. Polar interactions are illustrated as light yellow dashed lines, with corresponding bond lengths explicitly annotated in black. (J) Biofilm formation was quantified in PAO1 and isogenic mutants, including PAO1, Δ*bscR*, Δ*cheB*, Δ*bscR*Δ*cheB*, Δ*cheB*(*cheB*) and *cheB* point mutations with *bscR*-expressing, to elucidate the BscR-CheB interaction model. Data are presented as mean ± SD (three independent experiments). **, *P* < 0.01; ***, *P* < 0.001 *versus* the indicated group based on unpaired Student’s *t* test. n.s., not significant; EV, empty vector for the control.

Several previous studies have shown that the localization of motility regulatory components requires the assistance of various chemotaxis proteins. For instance, the c-di-GMP effector MapZ polar localization depends on CheR1 and PctA in *P. aeruginosa*, while the polar localization of CheO relies on CheVA and CheW in *C*. *jejuni* [23,24]. Our results showed that BscR mediates bacterial chemotaxis-relatived genes and is located at the poles of cells (Fig. 2E, F and G). BscR is likely a cytoplasmic protein, as it lacks any apparent signal peptide or transmembrane regions. These observations prompted us to explore whether chemotaxis proteins may influence the polar localization of BscR. To address this question, we introduced a *bscR*-*gfp* fusion gene into derivatives of *P. aeruginosa* in which chemotaxis genes *cheA*/*B*/*W*/*Z*/*R1*/*V*/*Y* or *motY* were deleted individually. We discovered that BscR-GFP lost its polar localization in the absence of CheB, suggesting that its cellular localization requires methyltransferase CheB (Fig. 5A and B). In contrast, BscR-GFP maintained its position at the cell poles despite lacking other selected chemotaxis-related genes (Fig. S11). To investigate the specific contribution of CheB in BscR-mediated regulation of swimming motility, we performed genetic complementation experiments by introducing the *bscR* gene into Δ*cheB*, Δ*cheW*, and Δ*cheZ* mutant strains. The results revealed that when *bscR* was expressed in the Δ*cheB* mutant, the restoration of BscR-mediated inhibition of swimming motility was specifically impaired. In contrast, this regulatory effect remained intact when *bscR* was expressed in both Δ*cheW* and Δ*cheZ* mutant backgrounds (Fig. 5C and D). Similarly, we observed that expression of *bscR* did not enhance biofilm production in the absence of *cheB*, nor did it occur with mutations in *cheW* and *cheZ* (Fig. 5E).

To further elucidate the localization of BscR at the cell poles and its regulatory role in chemotaxis-associated gene expression, BTH assays were conducted to investigate potential interactions between BscR and the chemotaxis-related proteins CheA, CheB, CheW, CheZ, CheR1, CheV, CheY, and MotY. The results showed that BscR likely interacts with CheB (Fig. 5F). We verified this potential interaction through an *in vitro* pull-down assay (Fig. 5G). Subsequently, we employed AlphaFold procedure to establish a probable interaction model between BscR and CheB. This interaction model showed that two molecules of BscR bind with two molecules of CheB (Fig. 5H). To further explore the interaction interface, we mutated three residues in BscR (H87, H129 and T139), that are thought to be involved in maintaining this interface (Fig. 5I). The BTH assay results demonstrated that mutating all three residues together, as well as individually mutating H129 or T139, abolished the interaction between BscR and CheB; however, mutation of H87 did not affect the interaction (Fig. S9B). Site-directed mutagenesis of specific amino acid residues (H129 and T139) in CheB, which are essential for CheB-BscR interaction, abolished BscR-mediated promotion of biofilm formation (Fig. 5J). Furthermore, we found that the biofilm formation capacity of the Δ*cheB*Δ*bscR* mutant was comparable to the Δ*cheB* mutant, suggesting the absence of additive effects in their regulatory roles on biofilm formation (Fig. 5J). This finding suggests that H87 may cooperate with other residues to ensure the stability of the interaction interface. The expression of BscR in the mutants indicated that mutations at amino acid positions 129 and 139 of CheB did not enhance biofilm formation compared to those in PAO1 strain. The expression levels of CheB with mutations at residues 87, 129, and 139 were consistent with those of the wild-type CheB, indicating that these three mutated amino acids did not affect the expression of CheB (Fig. S10B).

**Fig. 6:**
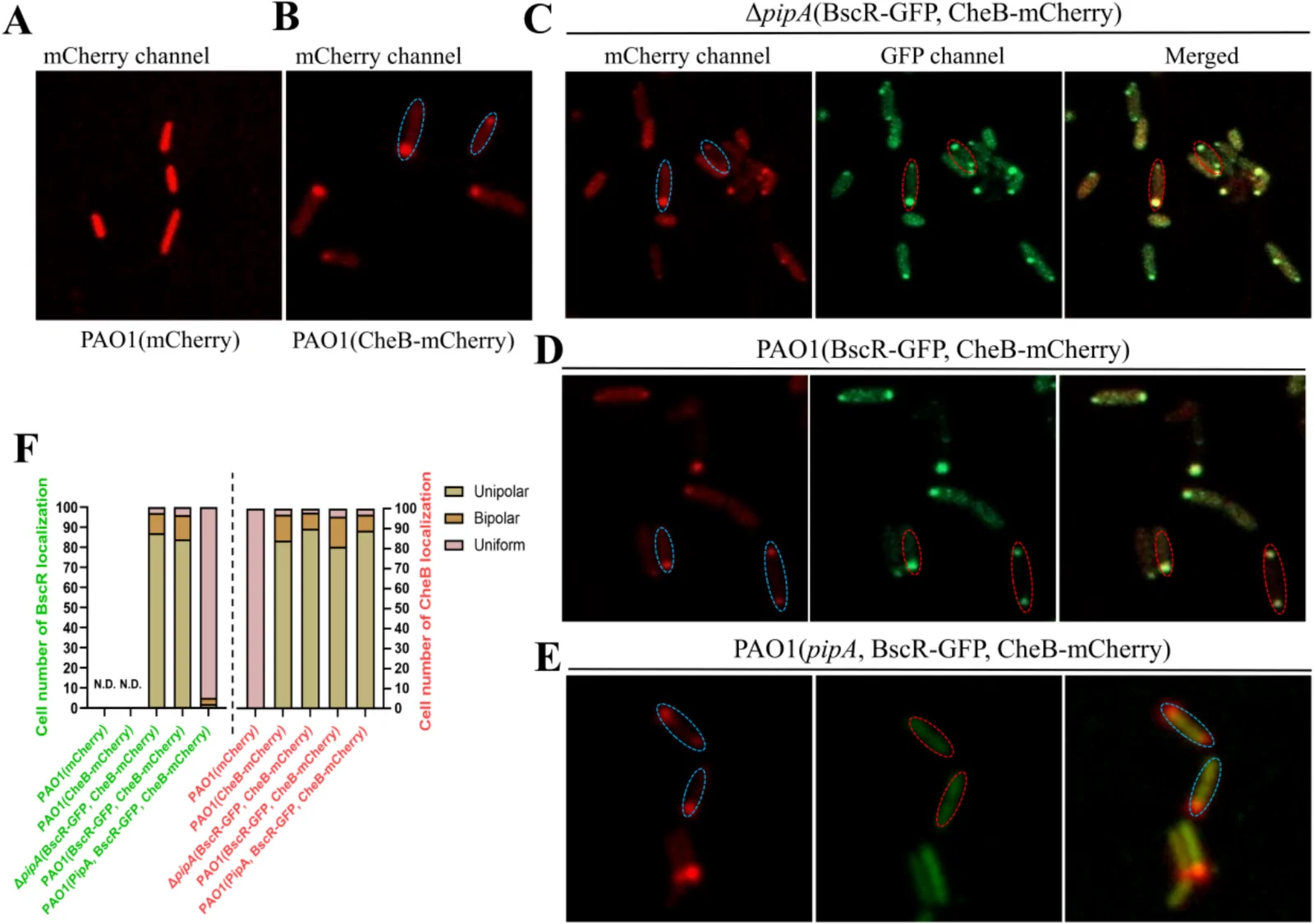
**Cellular localization of BscR-GFP and CheB-mCherry in *P. aeruginosa*.** (A and B) Fluorescence microscopy reveals the intracellular localization of mCherry (A) and CheB-mCherry (B) in wild-type PAO1. (C, D and E) Localization of BscR-GFP and CheB-mCherry in the Δ*pipA* mutant (C), PAO1 (D) and strain PAO1(*pipA*) in which *pipA* was expressed in PAO1 (E). (F) Quantification of the polar localization of BscR-GFP and CheB-mCherry in the strains shown in panels A-E (100 cells per strain from three independent experiments). N.D., not detected. Each experiment was performed in triplicate.

To investigate whether CheB and BscR co-localize within the cells. The CheB was labelled with mCherry to visualize its cellular distribution. We introduced the BscR-GFP fusion protein and the CheB-mCherry fusion protein into the Δ*pipA* mutant. Our observations indicated that mCherry is uniformly distributed throughout *P. aeruginosa* cells, whereas CheB-mCherry is concentrated at the cell poles (Fig. 6, Fig. 6, Fig. 6A, B and F). Notably, BscR-GFP and CheB-mCherry colocalized at the cell poles in the Δ*pipA* mutant (Fig. 6, Fig. 6, Fig. 6C and F). Similarly, in PAO1 strain, BscR-GFP and CheB-mCherry also demonstrated colocalization at the cell poles (Fig. 6, Fig. 6, Fig. 6D and F). Our results showed that PipA can disrupt cellular localization of BscR (Fig. 4A). Thus, we hypothesize that PipA may influence the interaction between BscR and CheB by disrupting the localization of BscR within the cell. In the PAO1(*pipA*) strain, where *pipA* was expressed in PAO1, we did not observe the distribution of BscR-GFP at the cell poles, while CheB-mCherry remained observable at the cell poles (Fig. 6E and F). These results indicate that BscR relies on CheB for its polar localization, and PipA exerts a disruptive effect on the colocalization between BscR and CheB localized at the cell poles.

### The loss of BscR enhances the pathogenicity of *P. aeruginosa*

**Fig. 7:**
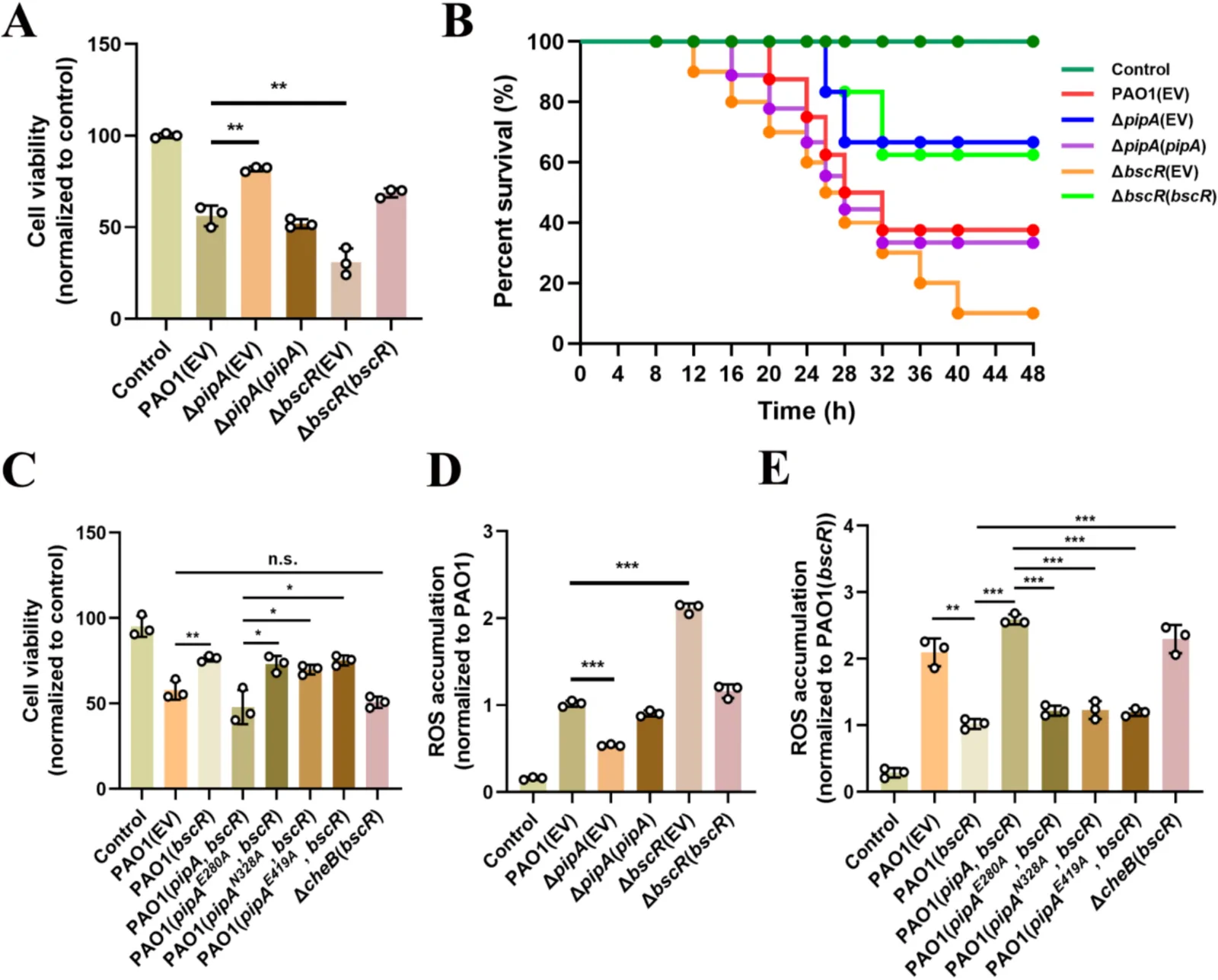
**BscR is a key contributor to the virulence regulation during *P. aeruginosa* infection.** (A) Quantitative analysis of A549 cell viability through an LDH assay following infection with PAO1, *bscR* and *pipA* deletion mutants, and their complementations at MOI 50, measured 4 h after inoculation. (B) Virulence assessment of the tested strains was conducted *in vivo* via the *G. mellonella* infection model, with 30 *Galleria mellonella* larvae counted per group. (C) The viability of A549 cells was assessed against PAO1, PAO1(*bscR*), and other strains, in which amino acid mutations of *pipA* were expressed in PAO1(*bscR*). (D and E) ROS production in A549 cells infected with PAO1, *bscR* and *pipA* deletion mutants, and their complementations (D) as well as other indicated strains (E) was detected by the DCFH-DA assay, measured 4 h post-inoculation. Data are presented as mean ± SD (three independent experiments). *, *P* < 0.05; **, *P* < 0.01; ***, *P* < 0.001 *versus* the indicated group based on unpaired Student’s *t* test. n.s., not significant; EV, empty vector for the control.

Considering the multiple phenotypes that are regulated by BscR, we decided to examine its significance in pathogenesis through a well-established cell infection model [39]. To this end, the wild-type PAO1, the Δ*bscR* knockout mutant, and the Δ*bscR*(*bscR*) complementation strain were inoculated into human lung cell line A549. It was demonstrated that compared to the wild-type PAO1, the absence of *bscR* notably reduces cell survival, with cell death levels of A549 cells induced by the Δ*bscR* strain reaching 70 % following a 4-hour infection (Fig. 7A). In contrast, the strain Δ*bscR*(*bscR*) restored cell survival to levels comparable to those of PAO1 (Fig. 7A). Moreover, we observed that cell survival is promoted following Δ*pipA* mutant infection (Fig. 7A). In the *G. mellonella* infection assay, the data exhibited that the Δ*bscR* strain led to a two-fold lower mortality compared to the PAO1 strain, whereas the Δ*pipA* mutant showed a survival rate twice that of PAO1 after 30 h of infection (Fig. 7B).

To explain the roles of PipA and CheB in the regulation of bacterial against A549 cells mediated by BscR, we assessed the virulence of the constructs PAO1(*pipA*, *bscR*), PAO1(*pipA^E280A^*, *bscR*), PAO1(*pipA^N328A^*, *bscR*), PAO1(*pipA^E419A^*, *bscR*) and Δ*cheB*(*bscR*). The successful co-expression of PipA (or its mutants) and BscR in these engineered strains was confirmed by Western blot analysis prior to infection (Fig. S12). Compared to the strain PAO1(*bscR*), we found a significant reduction in cell survival upon incubation with PAO1(*pipA*, *bscR*) (Fig. 7C). Conversely, the survival phenotype was restored following infection with the strains PAO1(*pipA^E280A^*, *bscR*), PAO1(*pipA^N328A^*, *bscR*) or PAO1(*pipA^E419A^*, *bscR*) strains (Fig. 7C). This effect may also be exerted by CheB, as Δ*cheB*(*bscR*) exhibited a phenotype similar to PAO1 strain (Fig. 7C). Reactive oxygen species (ROS) levels were induced post-bacterial infection, contributing to the disruption and elimination of pathogens [40]. The ROS levels subsequently was evaluated in A549 cells infected with indicated strains. The results revealed a two-fold increase in oxidative stress levels in cells exposed to the Δ*bscR* mutant compared to these treated with the PAO1, while a two-fold decrease in oxidative levels was observed in cells infected with the Δ*pipA* mutant compared to the wild-type PAO1 (Fig. 7D). The oxidative stress status in cells infected with PAO1(*pipA^E280A^*, *bscR*), PAO1(*pipA^N328A^*, *bscR*) or PAO1(*pipA^E419A^*, *bscR*) strains were comparable to those in PAO1(*bscR*)-infected cells (Fig. 7E). Compared to the expression of *bscR* in the PAO1 strain, the levels of ROS in the A549 exposed to the *bscR*-expressing *cheB* deletion mutant did not show a decrease (Fig. 7E). Together, these findings clearly demonstrate that PipA-BscR-CheB signaling cascade is crucial for regulating bacterial pathogenicity.

## Discussion

*P. aeruginosa* is a valid study understanding microbial pathogenesis and antibiotic resistance due to its capability to develop microbial communities and mobility, such traits being pivotal for bacterial persistence during infections [1]. During *P. aeruginosa* infections, the c-di-GMP signaling mechanism is essential in orchestrating the shift from swimming behavior to biofilm maturation [10]. Numerous proteins contribute to production and breakdown of c-di-GMP, playing crucial roles in its regulation [10]; however, the specific functional outputs regulated by these genes remain unclear. Our previous research demonstrated that the phosphodiesterase PipA modulates cellular c-di-GMP production, regulating biofilm development and swimming motility, but the downstream effects of the PipA regulatory pathway remain incompletely understood [25]. Here, we identified a BscR as a partner protein of PipA. BscR is localized at the cell poles and modulates bacterial movement and biofilm production in a CheB-dependent manner. PipA can interfere with the modulation of biofilm development, motility, and pathogenicity through BscR. The results demonstrate a PipA-BscR-CheB signaling pathway that finely tunes bacterial adaptive behaviors.

The canonical c-di-GMP pathway integrates environmental signals to coordinate the enzymatic activities of DGC and PDE, leading to effector activation [17]. This investigation reveals that PipA interacts with BscR, elucidating that the modulation of biofilm and swimming behavior mediated by PipA relies on BscR. Furthermore, *pipA* negatively regulates the transcriptional level of *bscR*. These data support that the BscR controls biofilm formation and motility by its interaction with PipA, revealing a new signaling pathway of PipA. This regulatory pathway is required for host colonization, as the motility and chemotactic behavior of *P. aeruginosa* are crucial for bacterial infection of the host [41]. Ours results showed that deletion of *bscR* increases death rate of A549 cells and *G. mellonella* during bacteria infection. The *in vivo* experiments demonstrated that the deletion of *cheB* in the Δ*bscR* mutant background reversed the death phenotype observed in the Δ*bscR* mutant infection, revealing that BscR-mediated virulence regulatory mechanism is important for *P. aeruginosa* pathogenicity. Although several transcriptional regulators in *P. aeruginosa*, using examples like BrlR and FleQ, have been discovered as c-di-GMP receptors and possess c-di-GMP binding motif, those motifs exhibit limited structural similarity [42,43]. We identified no c-di-GMP binding motif in the amino acid sequence of BscR when comparing it to known receptors, however, further investigation is warranted to determine whether BscR serves as receptor of c-di-GMP and to identify its potential target genes. The findings demonstrated that the c-di-GMP degradation activity of PipA is abolished in the Δ*bscR*Δ*pipA* double mutant. Moreover, ours results indicated that BscR does not influence PipA's PDE activity, and transcriptomic analysis reveals that BscR does not regulate the transcription of c-di-GMP-regulated genes. We speculate that BscR may modulate production of c-di-GMP through metabolic enzymes. Indeed, our transcriptomic data showed downregulation of c-di-GMP DGC, for example, *PA0847* and *PA1851*, and upregulation of PDE including *PA2567*. However, our attempts to obtain high-purity BscR protein using the *E. coli* protein expression system were unsuccessful, which has hindered further investigation into whether BscR regulates downstream targets directly or indirectly, as well as the function of c-di-GMP in BscR-mediated regulation.

*P. aeruginosa* resides in hosts under low iron conditions, where iron serves as a crucial nutrient for bacterial growth and an essential cofactor for many redox-active enzymes [33]. To acquire iron from their environment, bacteria must contend with its extremely low solubility in nature; consequently, many bacteria synthesize high-affinity iron chelators known as siderophores, along with specific cell surface receptors designed to recognize iron-siderophore complexes [33]. Under low iron conditions, pyochelin and pyoverdine are two key siderophores produced by *P. aeruginosa* [33,44]. Siderophores chelate ferric ions and facilitate their transport through outer membrane receptors, such as those involving FptA and TonB proteins, into the bacterial cytoplasm, from which the chelated ferric ions can subsequently be utilized or secreted into the extracellular environment [33,44]. Comparative transcriptome analysis revealed alterations in the expression of abundant genes associated with iron uptake in Δ*bscR* mutant relative to the parental PAO1. Phenotypic analyses combined with RT-qPCR confirmed marked decrease in pyoverdine production within the Δ*bscR* mutant. Similarly, Our previous RNA-seq results displayed that transcription of pyoverdine and pyochelin biosynthetic genes were decreased in the *pipA* deletion mutant [25]. The findings imply that the PipA-BscR signaling pathway participates in iron uptake mechanisms. Iron has been identified as an inducer of PDE activity, for example, the conformation of the PDE IsmP changes upon iron binding, subsequently regulating biofilm formation [18]. PipA possesses two PAS domains, which are primarily responsible for sensing environmental cues. A recent investigation predicted heme and FAD binding sites within the first and second PAS domains of PipA, respectively, however, no binding of either heme or FAD has been observed to date [45]. These observations suggest that PipA could potentially serve as a sensor for environmental iron levels.

Our study demonstrated that transcription of *bscR* in the parental strain PAO1 significantly reduced swarming and swimming motility. BscR suppresses flagellar biosynthesis-related gene expression and blocks flagellar assembly. Upon further investigation, we found that BscR exerts transcriptional repression on genes associated with chemotaxis. In *P. aeruginosa*, chemotaxis proteins like CheA and CheY, which are localized at the cell poles, perform critical signal transduction functions [35]. The regulatory role of BscR in biofilm development and bacteria motility is further demonstrated by its polar localization. The polar localization of MapZ, a methyltransferase-associated PilZ protein, is sequestered to the cell poles via components of the chemoreceptor array, CheR1 and PctA [23]. We found that the polar localization of BscR is contingent upon the formation of the chemosensory array, as the absence of CheB led to the dispersion of BscR away from the cell poles. CheB regulates the cell's response to environmental changes by lowering the sensitivity of membrane-bound chemotactic receptors through the removal of methyl groups from them [46]. We demonstrated that the interaction between BscR and CheB was confirmed through protein–protein interaction assays, which implies a functional linkage of BscR to the methylation process in chemosensory arrays.

Based on molecular docking studies, we propose a potential interaction model between PipA and BscR, identifying the residues R272 and E280 within the PAS domain of PipA, along with residues N325, N328, R348, R359, R403 and E419 within the GGDEF domain of PipA, as key components constituting the PipA-BscR interface. Our experimental results showed that PipA degrades c-di-GMP into pGpG, however, GTP does not converted into c-di-GMP, suggesting the absence of DGC activity in the GGDEF domain under *in vitro* (Fig. S13). The mutations at amino acid positions E280, R348 and R359 of PipA do not affect biofilm formation regulated by this protein. Domain-directed mutation analysis revealed that deletion of the PAS and GGDEF domains abolished biofilm dispersion regulated by PipA. These results suggest that BscR regulates biofilm formation and motility independent of PDE function of PipA, while the PDE activity appears to be contingent upon the integrity of these two domains (Fig. S14). Fluorescence microscopy revealed that BscR-GFP co-localizes with CheB-mCherry at the cell poles in Δ*pipA* mutant. Mutations in CheB abolished the regulatory influence of BscR on biofilm development and bacterial movement. Under conditions of PipA excess, the interaction between PipA and BscR disrupted the polar localization of BscR-GFP, resulting in a loss of its regulatory role on biofilm and motility. In PAO1 cells, we observed that BscR-GFP and CheB-mCherry are localized at the cell poles. The regulatory mechanism of BscR involves spatial organization within the cell. Specifically, its polar localization is critical for coordinating biofilm development and motility-related processes. We hypothesize that under normal growth conditions, BscR exhibits a higher affinity for CheB, which prevents its interaction with PipA. However, under specific inducing conditions that stimulate excess PipA production, the functionality of BscR is compromised. Despite extensive efforts to identify physiological conditions that induce PipA overexpression, our systematic screening failed to reveal any significant regulatory triggers.

## Conclusion

In summary, while certain PDEs and DGCs globally regulate c-di-GMP levels, most exhibit localized effects with limited regulatory scope. This study demonstrates that PipA achieves global regulation of bacterial behavior through a unique regulatory cascade involving BscR and CheB. Specifically, we identified a tripartite interaction network where PipA coordinates with BscR and CheB to precisely control bacterial motility and biofilm formation. Our findings reveal an efficient regulatory strategy that facilitates precise spatiotemporal control of bacterial behavior through targeted protein–protein interactions. This regulatory model provides an insight into PDE function in bacterial signaling networks, demonstrating that protein interaction-based regulation can complement and extend beyond traditional second messenger modulation mechanisms.

## Declaration of competing interest

*The authors declare that they have no known competing financial interests or personal relationships that could have appeared to influence the work reported in this paper.*

## References

[1] Qin S., Xiao W., Zhou C., Pu Q., Deng X., Lan L. (2022). *Pseudomonas aeruginosa*: pathogenesis, virulence factors, antibiotic resistance, interaction with host, technology advances and emerging therapeutics. Signal Transduct Target Ther.

[2] Coleman S., Pletzer D., Hancock R.E.W. (2021). Contribution of swarming motility to dissemination in a *Pseudomonas aeruginosa* murine skin abscess infection model. J Infect Dis.

[3] Sauer K., Stoodley P., Goeres D.M., Hall-Stoodley L., Burmolle M., Stewart P.S. (2022). The biofilm life cycle: expanding the conceptual model of biofilm formation. Nat Rev Microbiol.

[4] Qu J.X., Cai Z., Duan X.K., Zhang H., Cheng H., Han S.H. (2022). *Pseudomonas aeruginosa* modulates alginate biosynthesis and type VI secretion system in two critically ill COVID-19 patients. Cell Biosci.

[5] Lee J., Zhang L. (2015). The hierarchy quorum sensing network in *Pseudomonas aeruginosa*. Protein Cell.

[6] Rasamiravaka T., Labtani Q., Duez P., Jaziri M.E. (2015). The formation of biofilms by *Pseudomonas aeruginosa*: a review of the natural and synthetic compounds interfering with control mechanisms. Biomed Res Int.

[7] Zhou T., Huang J., Liu Z., Xu Z., Zhang L.H. (2021). Molecular mechanisms underlying the regulation of biofilm formation and swimming motility by FleS/FleR in *Pseudomonas aeruginosa*. Front Microbiol.

[8] Liu J.H., Zhang Y.Z., Zhou N., He J.L., Xu J., Cai Z. (2024). Bacmethy: a novel and convenient tool for investigating bacterial DNA methylation pattern and their transcriptional regulation effects. iMeta.

[9] Jenal U., Reinders A., Lori C. (2017). Cyclic di-GMP: second messenger extraordinaire. Nat Rev Microbiol.

[10] Valentini M., Filloux A. (2016). Biofilms and cyclic di-GMP (c-di-GMP) signaling: lessons from *Pseudomonas aeruginosa* and other bacteria. J Biol Chem.

[11] Povolotsky T.L., Hengge R. (2012). 'Life-style' control networks in *Escherichia coli*: signaling by the second messenger c-di-GMP. J Biotechnol.

[12] Merritt J.H., Brothers K.M., Kuchma S.L., O'Toole G.A. (2007). SadC reciprocally influences biofilm formation and swarming motility via modulation of exopolysaccharide production and flagellar function. J Bacteriol.

[13] An S., Wu J., Zhang L.H. (2010). Modulation of *Pseudomonas aeruginosa* biofilm dispersal by a cyclic-Di-GMP phosphodiesterase with a putative hypoxia-sensing domain. Appl Environ Microbiol.

[14] Chen Y.C., Yuan M.J., Mohanty A., Yam J.K.H., Liu Y., Chua S.L. (2015). Multiple diguanylate cyclase-coordinated regulation of pyoverdine synthesis in *Pseudomonas aeruginosa*. Environ Microbiol Rep.

[15] Chua S.L., Sivakumar K., Rybtke M., Yuan M.J., Andersen J.B., Nielsen T.E. (2015). C-di-GMP regulates *Pseudomonas aeruginosa* stress response to tellurite during both planktonic and biofilm modes of growth. Sci Rep.

[16] Xin L.Y., Zeng Y.K., Sheng S., Chea R.A., Liu Q., Li H.Y. (2016). Regulation of flagellar motor switching by c-di-GMP phosphodiesterases in *Pseudomonas aeruginosa*. J Biol Chem.

[17] Liu X., Cao B., Yang L., Gu J.D. (2022). Biofilm control by interfering with c-di-GMP metabolism and signaling. Biotechnol Adv.

[18] Zhan X.L., Zhang K., Wang C.C., Fan Q., Tang X.J., Zhang X. (2024). A c-di-GMP signaling module controls responses to iron in *Pseudomonas aeruginosa*. Nat Commun.

[19] Kong W., Luo W., Wang Y., Liu Y., Tian Q., Zhao C. (2022). Dual GGDEF/EAL-domain protein RmcA controls the type III secretion system of *Pseudomonas aeruginosa* by interaction with CbrB. ACS Infect Dis.

[20] Wuichet K., Zhulin I.B. (2010). Origins and diversification of a complex signal transduction system in prokaryotes. Sci Signal.

[21] Seymour J.R., Brumley D.R., Stocker R., Raina J.B. (2024). Swimming towards each other: the role of chemotaxis in bacterial interactions. Trends Microbiol.

[22] Chanchal B.P., Raghav S., Goswami H.N., Jain D. (2021). The antiactivator FleN uses an allosteric mechanism to regulate σ^54^-dependent expression of flagellar genes in *Pseudomonas aeruginosa*. Sci Adv.

[23] Xu L., Xin L., Zeng Y., Yam J.K., Ding Y., Venkataramani P. (2016). A cyclic di-GMP-binding adaptor protein interacts with a chemotaxis methyltransferase to control flagellar motor switching. Sci Signal.

[24] Mo R., Ma W., Zhou W., Gao B. (2022). Polar localization of CheO under hypoxia promotes *Campylobacter jejuni* chemotactic behavior within host. PLoS Pathog.

[25] Cai Y.M., Yu K.W., Liu J.H., Cai Z., Zhou Z.H., Liu Y. (2022). The c-di-GMP phosphodiesterase PipA (PA0285) regulates autoaggregation and Pf4 bacteriophage production in *Pseudomonas aeruginosa* PAO1. Appl Environ Microbiol.

[26] Welsh M.A., Eibergen N.R., Moore J.D., Blackwell H.E. (2015). Small molecule disruption of quorum sensing cross-regulation in *Pseudomonas aeruginosa* causes major and unexpected alterations to virulence phenotypes. J Am Chem Soc.

[27] Filloux A., Ramos J.L., Filloux A., Ramos J.L. (2014). *Pseudomonas* methods and protocols.

[28] Kuchma S.L., Brothers K.M., Merritt J.H., Liberati N.T., Ausubel F.M., O'Toole G.A. (2007). BifA, a cyclic-Di-GMP phosphodiesterase, inversely regulates biofilm formation and swarming motility by *Pseudomonas aeruginosa* PA14. J Bacteriol.

[29] Hak A.A., Zedan H.H., El-Mahallawy H.A., El-Sayyad G.S., Zafer M.M. (2024). *In Vivo* and *in Vitro* activity of colistin-conjugated bimetallic silver-copper oxide nanoparticles against Pandrug-resistant *Pseudomonas aeruginosa*. BMC Microbiol.

[30] Friedman L., Kolter R. (2004). Genes involved in matrix formation in *Pseudomonas aeruginosa* PA14 biofilms. Mol Microbiol.

[31] Tian M., Wu Z., Zhang R., Yuan J. (2022). A new mode of swimming in singly flagellated *Pseudomonas aeruginosa*. PNAS.

[32] Rada B., Leto T.L. (2013). Pyocyanin effects on respiratory epithelium: relevance in *Pseudomonas aeruginosa* airway infections. Trends Microbiol.

[33] Schalk I.J., Perraud Q. (2023). *Pseudomonas aeruginosa* and its multiple strategies to access iron. Environ Microbiol.

[34] Raghav S., Prajapati R., Jain D. (2025). Role of FlhF and its domains in the assembly of a polar flagellum in *P. aeruginosa*. J Bacteriol.

[35] Güvener Z.T., Tifrea D.F., Harwood C.S. (2006). Two different *Pseudomonas aeruginosa* chemosensory signal transduction complexes localize to cell poles and form and remould in stationary phase. Mol Microbiol.

[36] Rybtke M.T., Borlee B.R., Murakami K., Irie Y., Hentzer M., Nielsen T.E. (2012). Fluorescence-based reporter for gauging cyclic di-GMP levels in *Pseudomonas aeruginosa*. Appl Environ Microbiol.

[37] Guan C., Huang Y., Zhou Y., Han Y., Liu S., Liu S. (2024). FlhF affects the subcellular clustering of WspR through HsbR in *Pseudomonas aeruginosa*. Appl Environ Microbiol.

[38] Jumper J., Evans R., Pritzel A., Green T., Figurnov M., Ronneberger O. (2021). Highly accurate protein structure prediction with AlphaFold. Nature.

[39] Wang J., Dong Y., Zhou T., Liu X., Deng Y., Wang C. (2013). *Pseudomonas aeruginosa* cytotoxicity is attenuated at high cell density and associated with the accumulation of phenylacetic acid. PLoS One.

[40] West A.P., Brodsky I.E., Rahner C., Woo D.K., Erdjument-Bromage H., Tempst P. (2011). TLR signalling augments macrophage bactericidal activity through mitochondrial ROS. Nature.

[41] Wadhwa N., Berg H.C. (2022). Bacterial motility: machinery and mechanisms. Nat Rev Microbiol.

[42] Hickman J.W., Harwood C.S. (2008). Identification of FleQ from *Pseudomonas aeruginosa* as a c-di-GMP-responsive transcription factor. Mol Microbiol.

[43] Chambers J.R., Liao J., Schurr M.J., Sauer K. (2014). BrlR from *Pseudomonas aeruginosa* is a c-di-GMP-responsive transcription factor. Mol Microbiol.

[44] Schalk I.J., Guillon L. (2013). Pyoverdine biosynthesis and secretion in *Pseudomonas aeruginosa*: implications for metal homeostasis. Environ Microbiol.

[45] Eilers K., Yam J.K.H., Liu X., Goh Y.F., To K.N., Paracuellos P. (2024). The dual GGDEF/EAL domain enzyme PA0285 is a *Pseudomonas* species housekeeping phosphodiesterase regulating early attachment and biofilm architecture. J Biol Chem.

[46] Sampedro I., Parales R.E., Krell T., Hill J.E. (2015). *Pseudomonas* chemotaxis. FEMS Microbiol Rev.

