# Supplementary material for: Interaction of the polar localization protein BscR with phosphodiesterase PipA regulates virulence strategies in Pseudomonas aeruginosa
Source: J Adv Res. 2025 Dec 28;87:387–403. doi: 10.1016/j.jare.2025.12.048 (PMC13539228; doi:10.1016/j.jare.2025.12.048)
Supplement: Supplementary file 1 [file mmc1.pdf]

Supplemental Information

Interaction of the polar localization protein BscR with phosphodiesterase PipA regulates virulence strategies in *Pseudomonas aeruginosa*

Tian Zhou[a,b,*], Anmin Ren[b], Chenchen Wang[b], Yizhou Zhang[b,c], Jie Deng[b], Yang Liu[c], Tianyuan Jia[b,d], Haihua Liang[b], Liang Yang[b, d, *]

[a] Ecological Breeding Department, Guangdong Laboratory for Lingnan Modern Agriculture, Heyuan 517000, P. R. China

[b] Department of Pharmacology, Joint Laboratory of Guangdong-Hong Kong Universities for Vascular Homeostasis and Diseases, SUSTech Homeostatic Medicine Institute, School of Medicine, Southern University of Science and Technology, Shenzhen 518055, P. R. China

[c] Southern University of Science and Technology Hospital, Shenzhen 518055, P. R. China

[d] National Clinical Research Center for Infectious Disease, Shenzhen Third People's Hospital, Shenzhen 518055, P. R. China

* Correspondence:

Tian Zhou (824425421@qq.com)

Liang Yang (yangl@sustech.edu.cn)

Running title: BscR regulates bacterial virulence factors

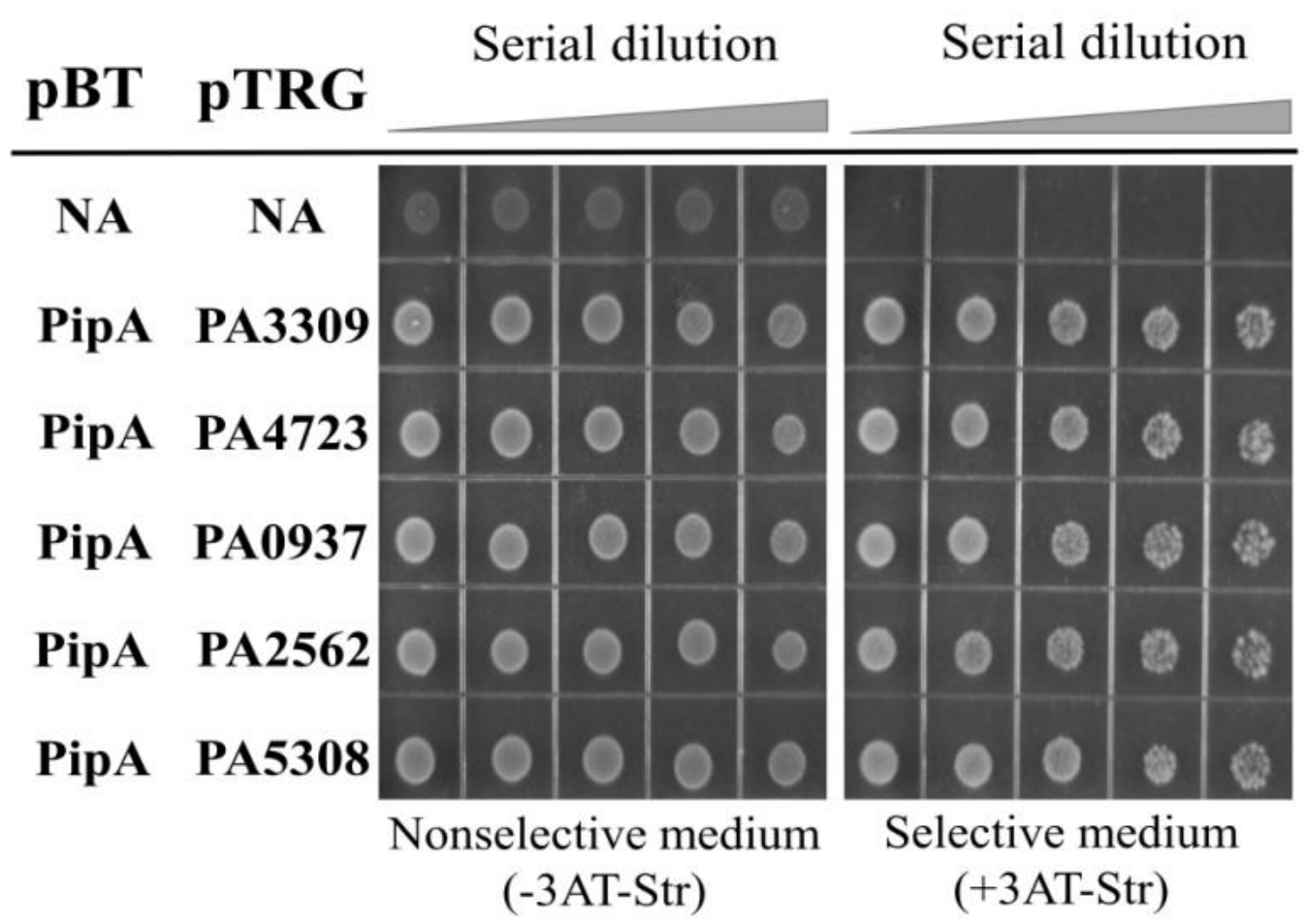

Fig. S1. Bacterial two-hybrid (BTH) assay validating interactions between PipA and candidate proteins identified by CoIP-MS. Negative control (NA): empty pBT and pTRG vectors. All experiments were performed in triplicate.

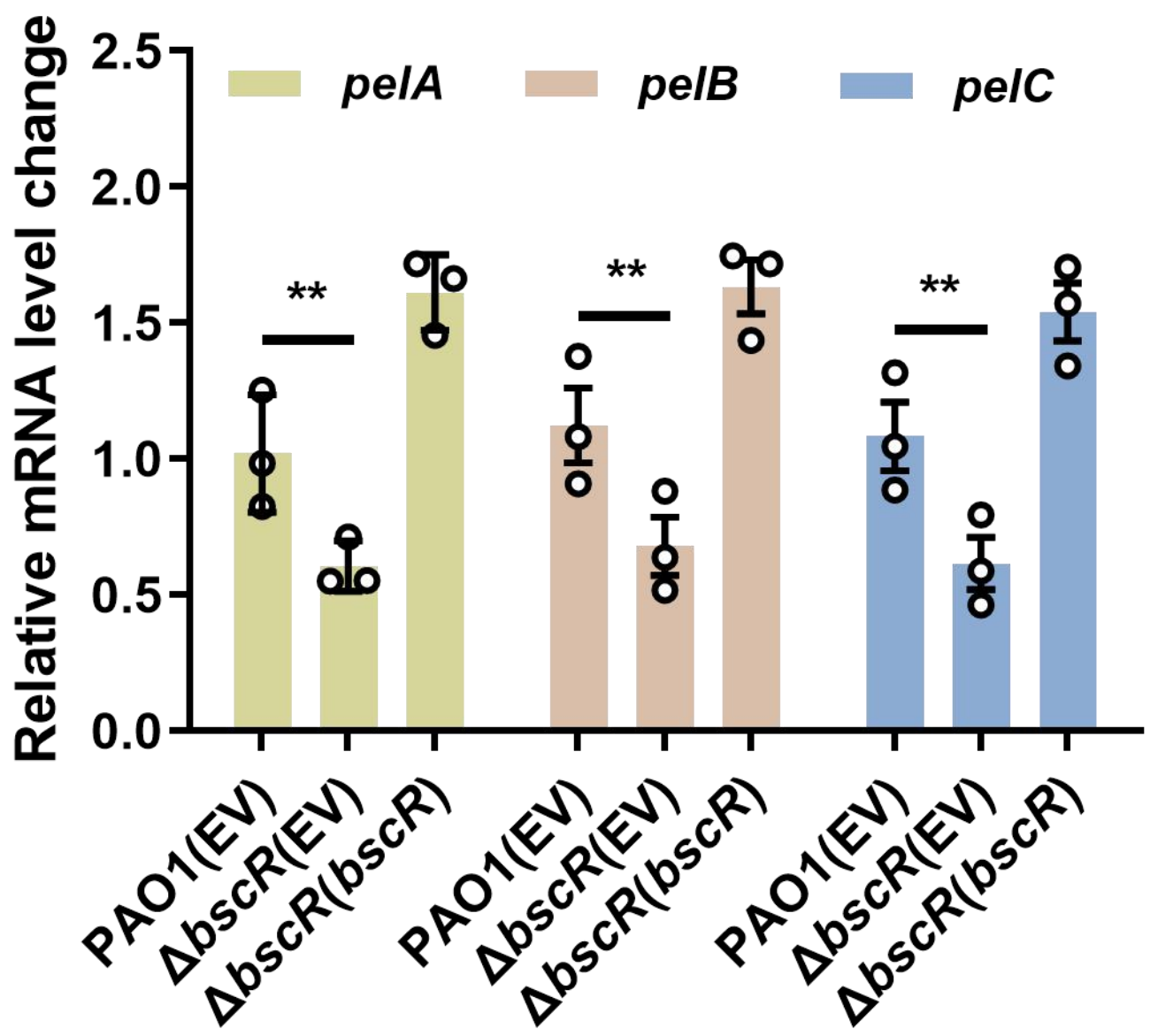

Fig. S2. Expression levels of *pelA*, *pelB*, and *pelC* mRNA were detected via RT-qPCR in PAO1, Δ*bscR* mutant, and Δ*bscR*(*bscR*) strains. Data are presented as mean ± SD (three independent experiments). **, $P < 0.01$ *versus* PAO1(EV) based on unpaired Student's *t* test. EV, empty vector for the control.

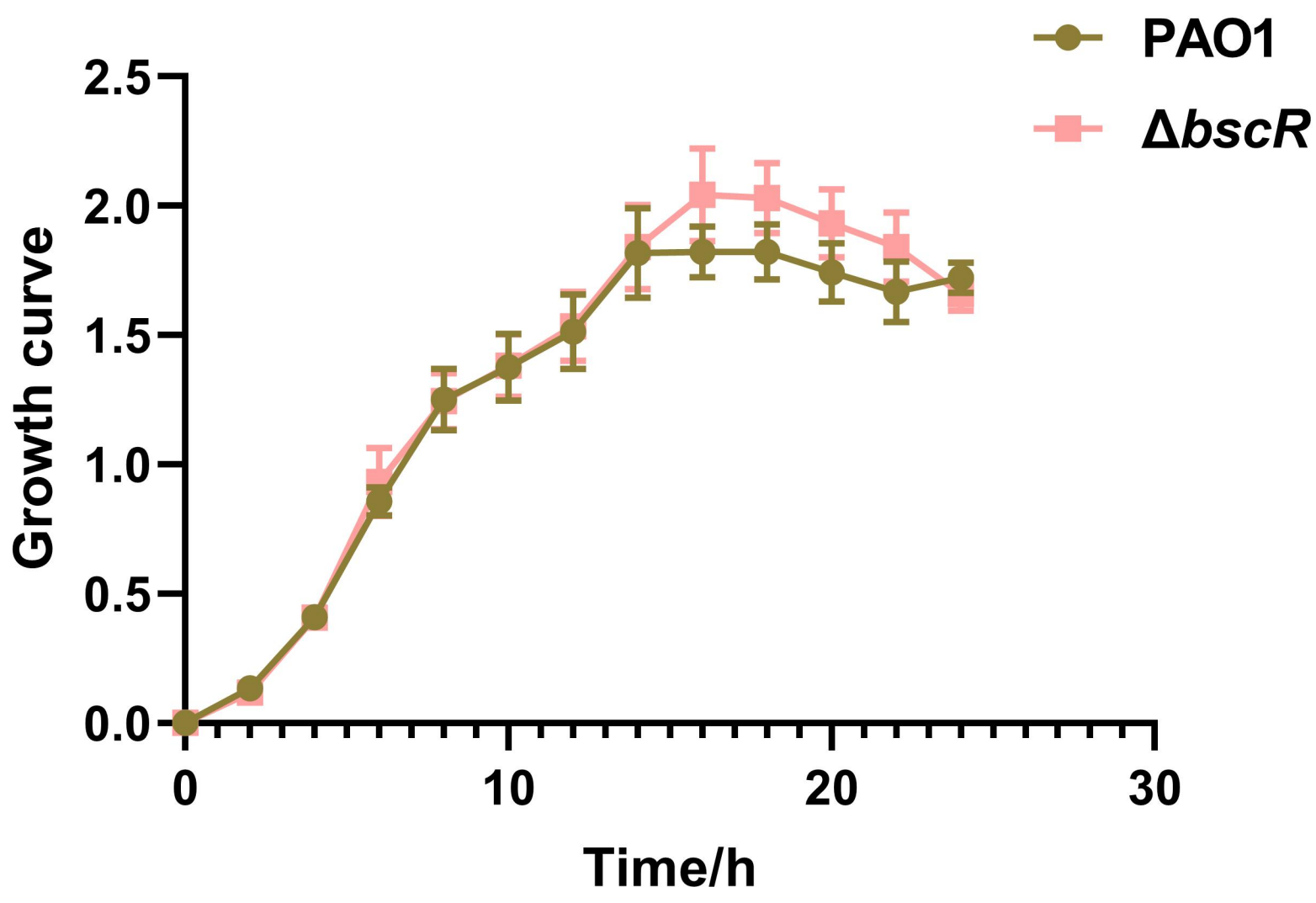

Fig. S3. Bacterial growth was determined by measuring the OD_{600} using a microplate reader (TECAN). Data are presented as mean ± SD (five independent experiments).

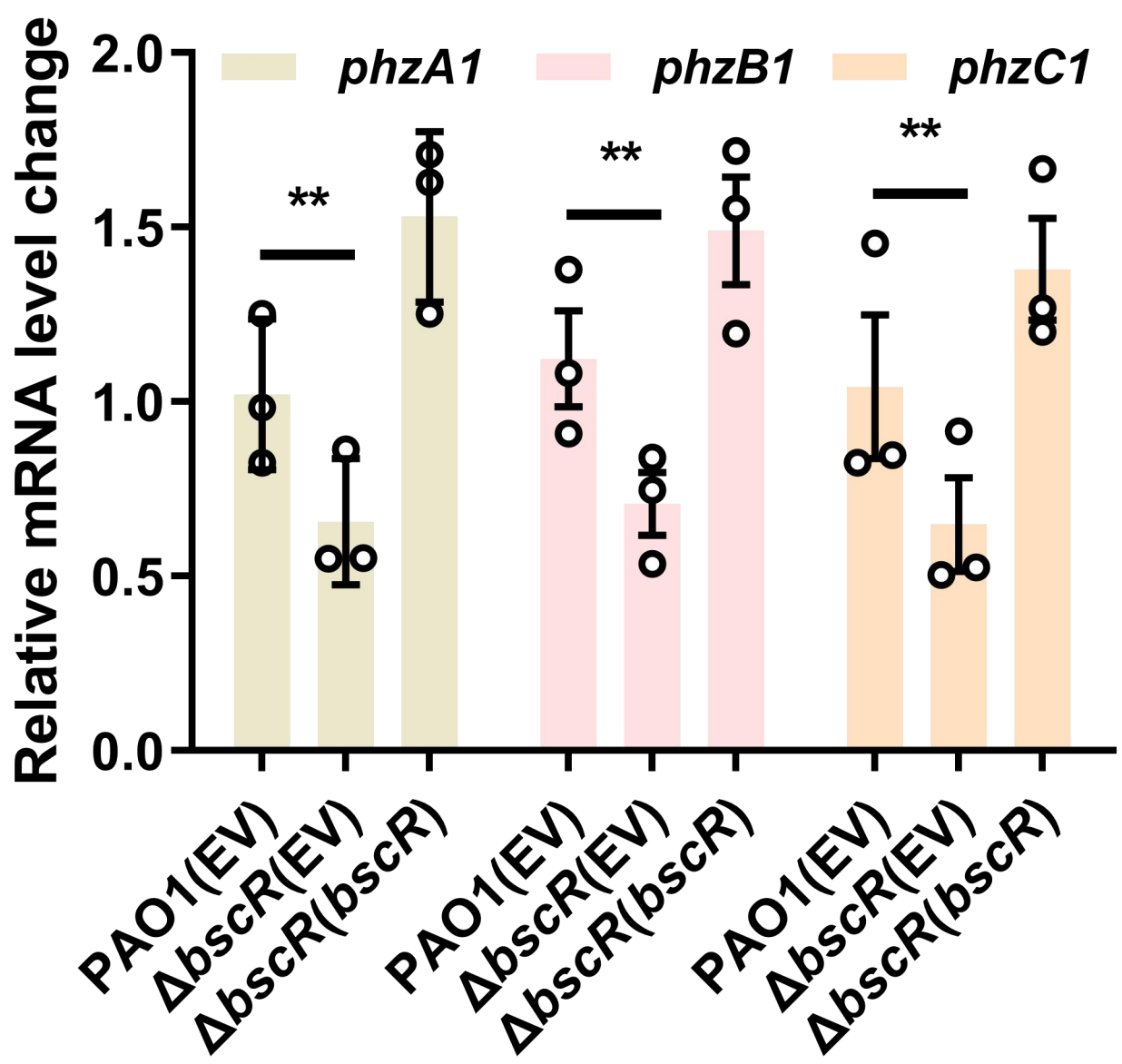

Fig. S4. In PAO1, Δ*bscR* mutant, and Δ*bscR*(*bscR*) strains, transcriptional levels of *phzA1*, *phzB1* and *phzC1* were assessed. Data are presented as mean ± SD (three independent experiments). **, $P < 0.01$ *versus* PAO1(EV) based on unpaired Student's *t* test. EV, empty vector for the control.

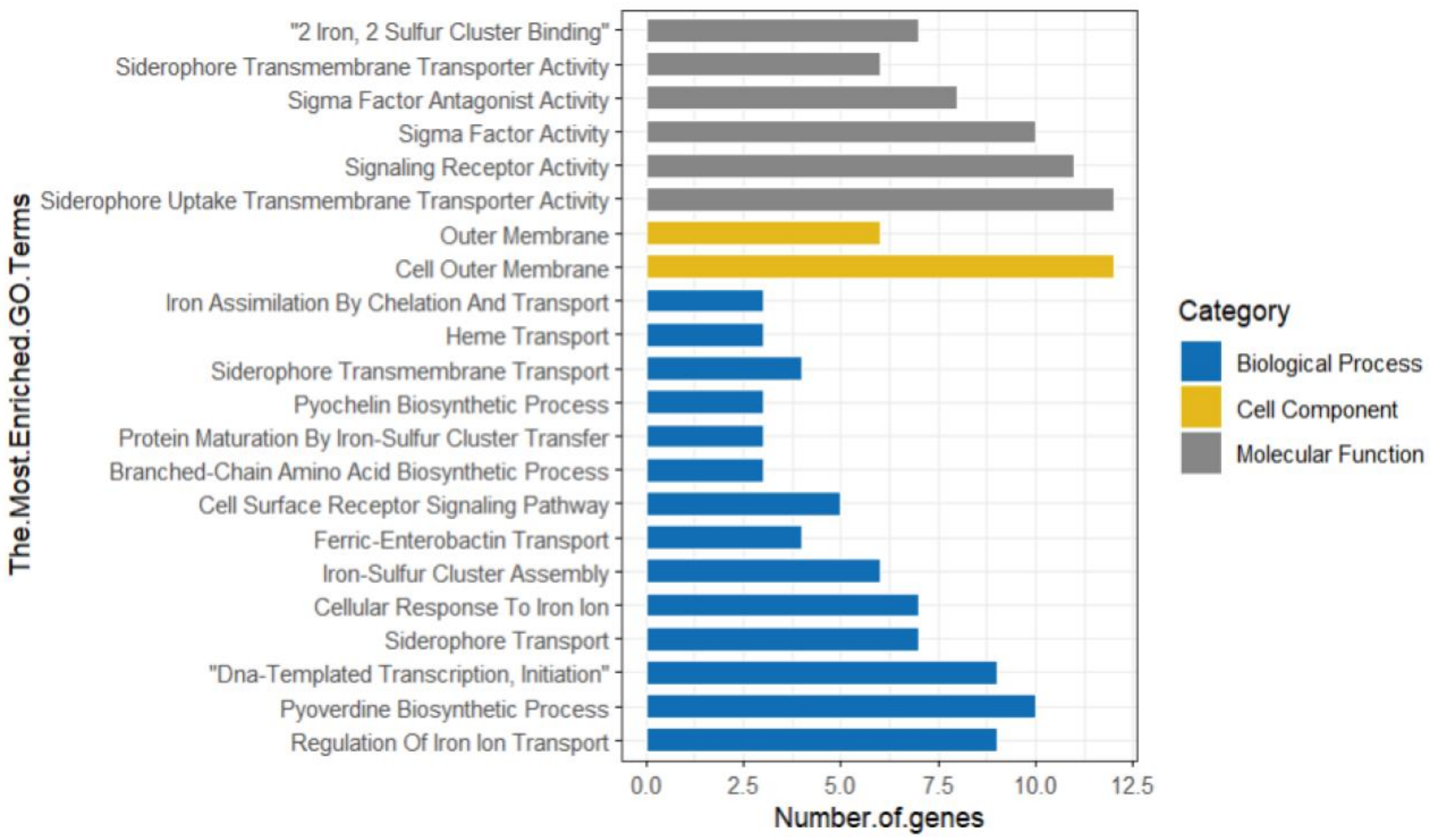

Fig. S5. GO term enrichment analysis comparing DEGs in Δ*bscR* mutant *versus* PAO1 strains.

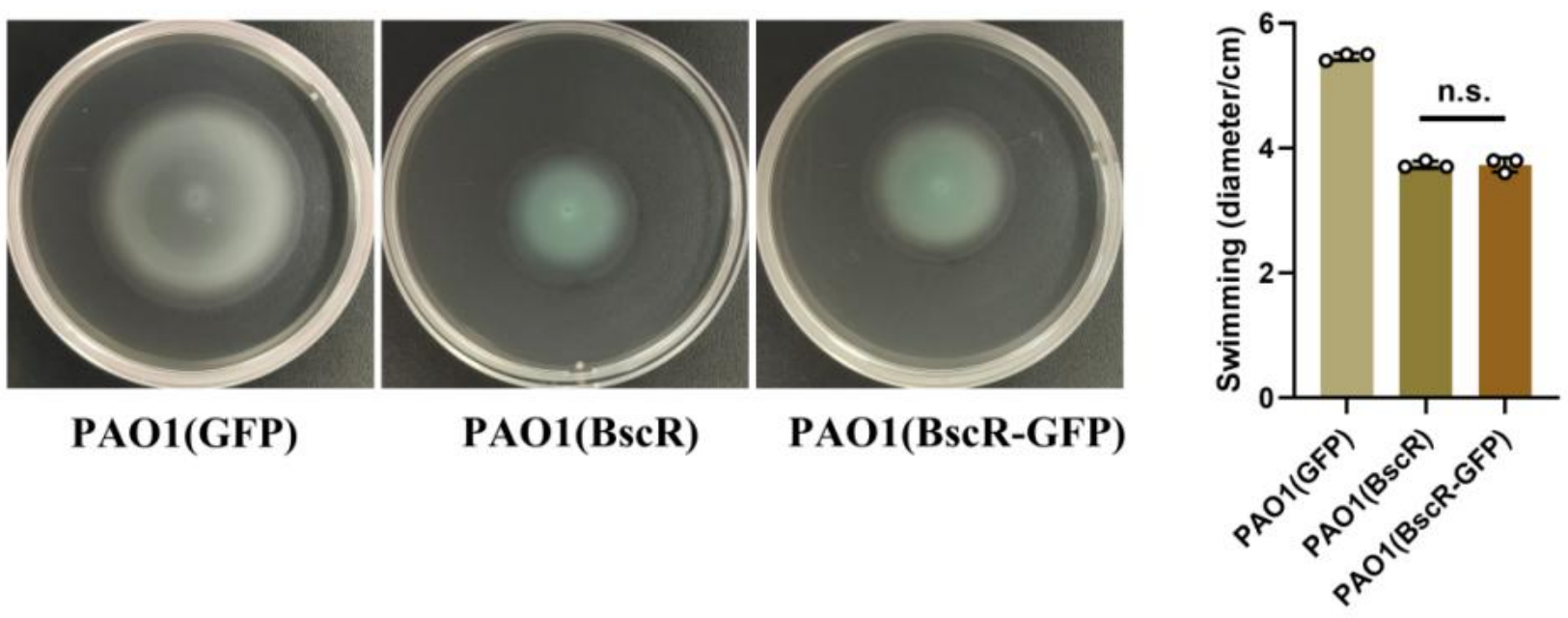

Fig. S6. The C-terminal fusion of the GFP tag does not impair regulation of BscR for swimming motility. The left panel demonstrates the swimming motility exhibited by the indicated bacterial strains. Representative images are shown. The diameter of the swimming zones was measured (right panel). Data are presented as mean ± SD (three independent experiments). n.s., not significant.

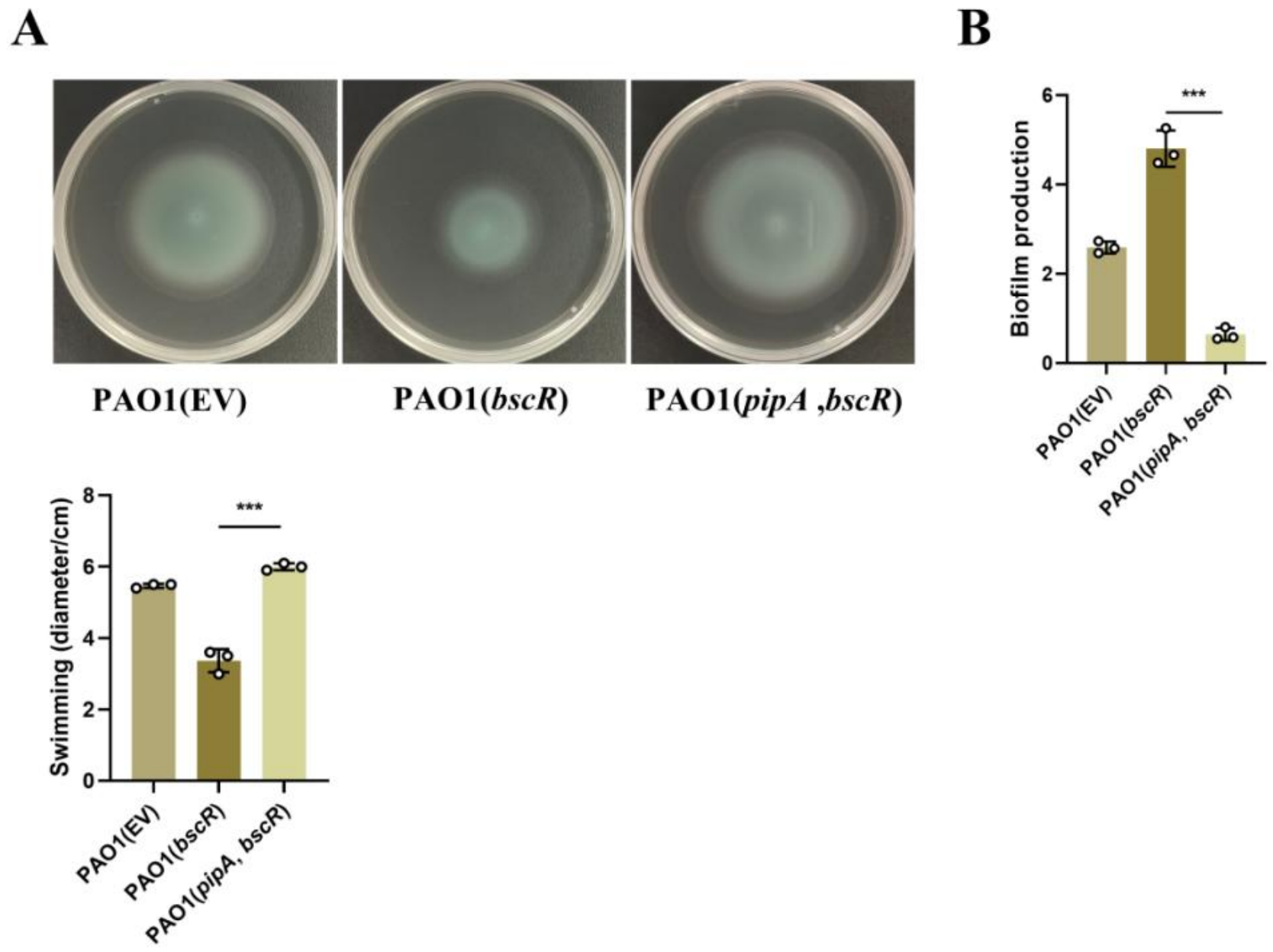

Fig. S7. PipA disrupts the function of BscR in regulating biofilm and swimming. (A) Representative images of swimming motility (up panel) and the calculated swimming diameters of PAO1, Δ*bscR* and PAO1(*pipA*, *bscR*) strains (down panel). (B) Biofilm production of PAO1, Δ*bscR* and PAO1(*pipA*, *bscR*) strains was quantified. Data are presented as mean ± SD (three independent experiments). ***, $P < 0.001$ *versus* PAO1(*bscR*) based on unpaired Student's *t* test. EV, empty vector for the control.

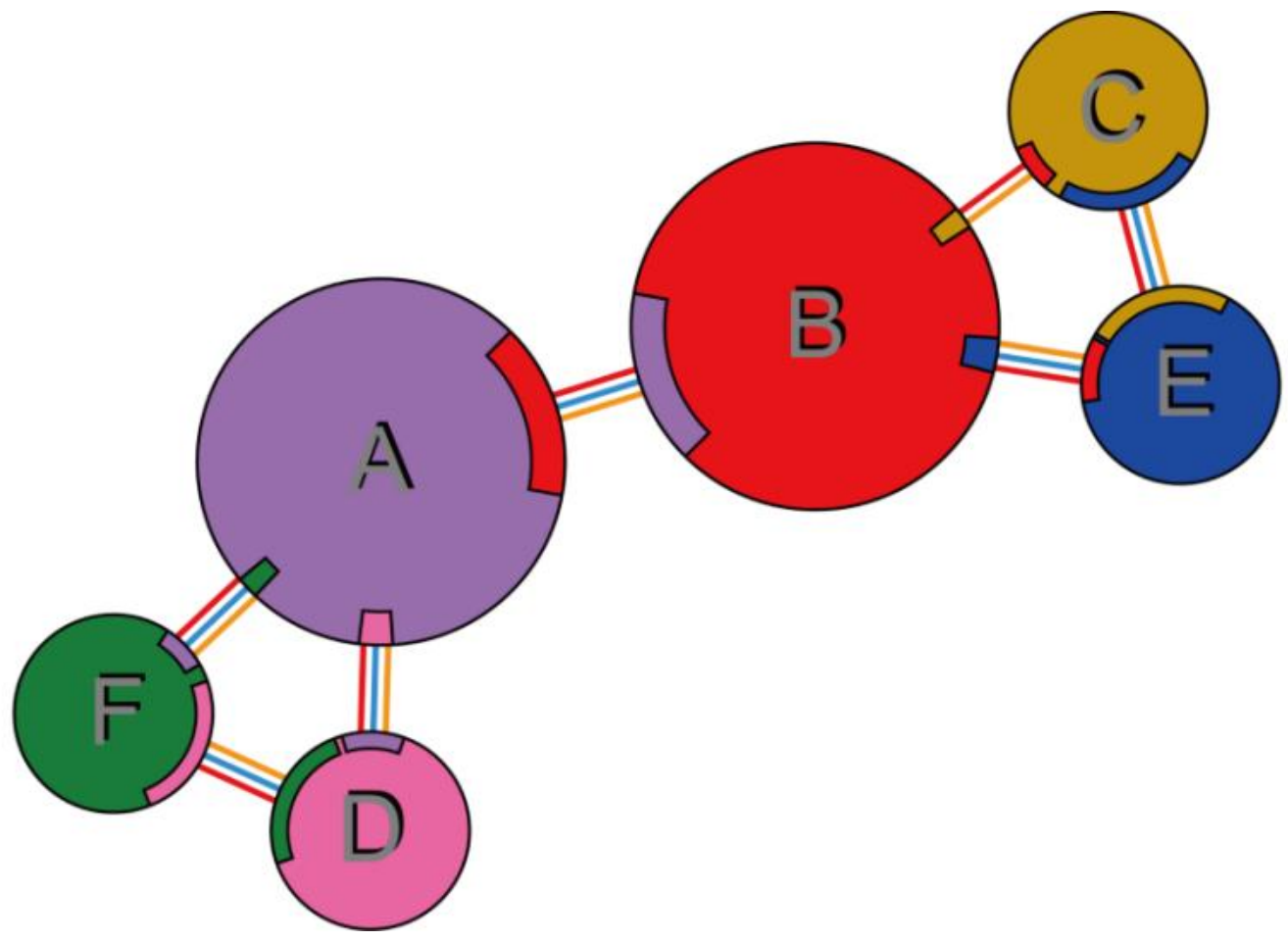

Fig. S8. Schematic of the interactions between PipA and BscR. The interactions between chains are visualized through color-coded lines: salt bridges (red), hydrogen bonds (blue), and non-bonded contacts (orange). A=B=PipA; C=D=E=F=BscR.

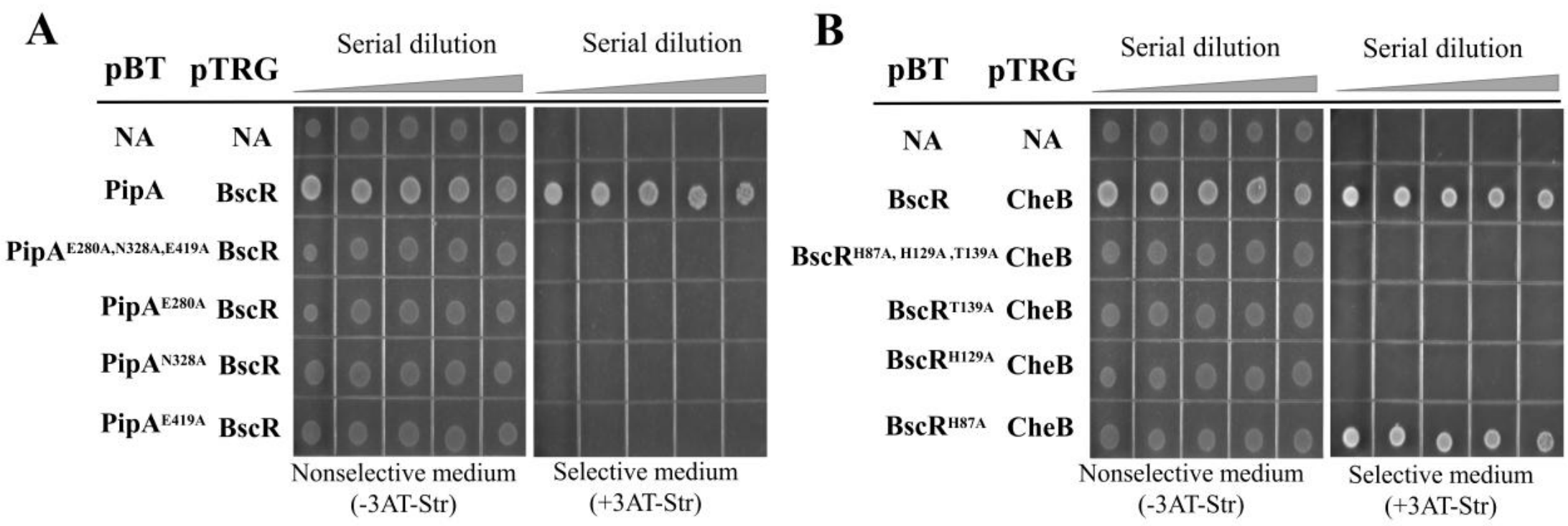

Fig. S9. BTH assays validate the docking model for PipA-BscR and BscR-CheB. (A) BTH assays demonstrate interactions between BscR and PipA or its mutants. (B) BTH assays show interactions between CheB and BscR or mutated BscR. Negative control (NA): empty pBT and pTRG vectors. All experiments were performed in triplicate.

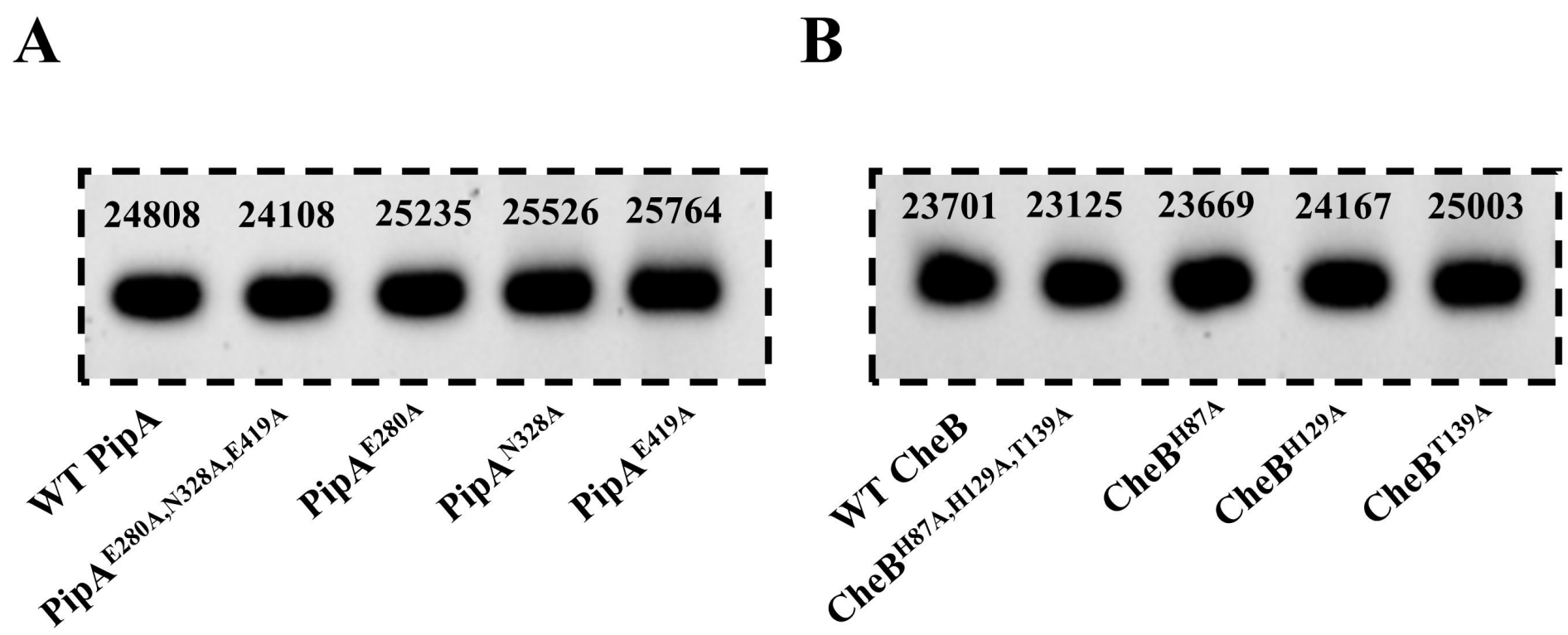

Fig. S10. Detection of protein expression levels of PipA and BscR and their amino acid mutants. (A) Wild-type PipA and its mutant variants, which carry amino acid substitutions at positions 280, 328, and 419, were analyzed for their expression levels. (B) Wild-type CheB and its mutants with substitutions at amino acid positions 87, 129, and 139 were assessed for expression. Protein levels were quantified by grayscale analysis using ImageJ software. Numerical values indicating relative band intensity are shown above each lane. All experiments were performed in triplicate.

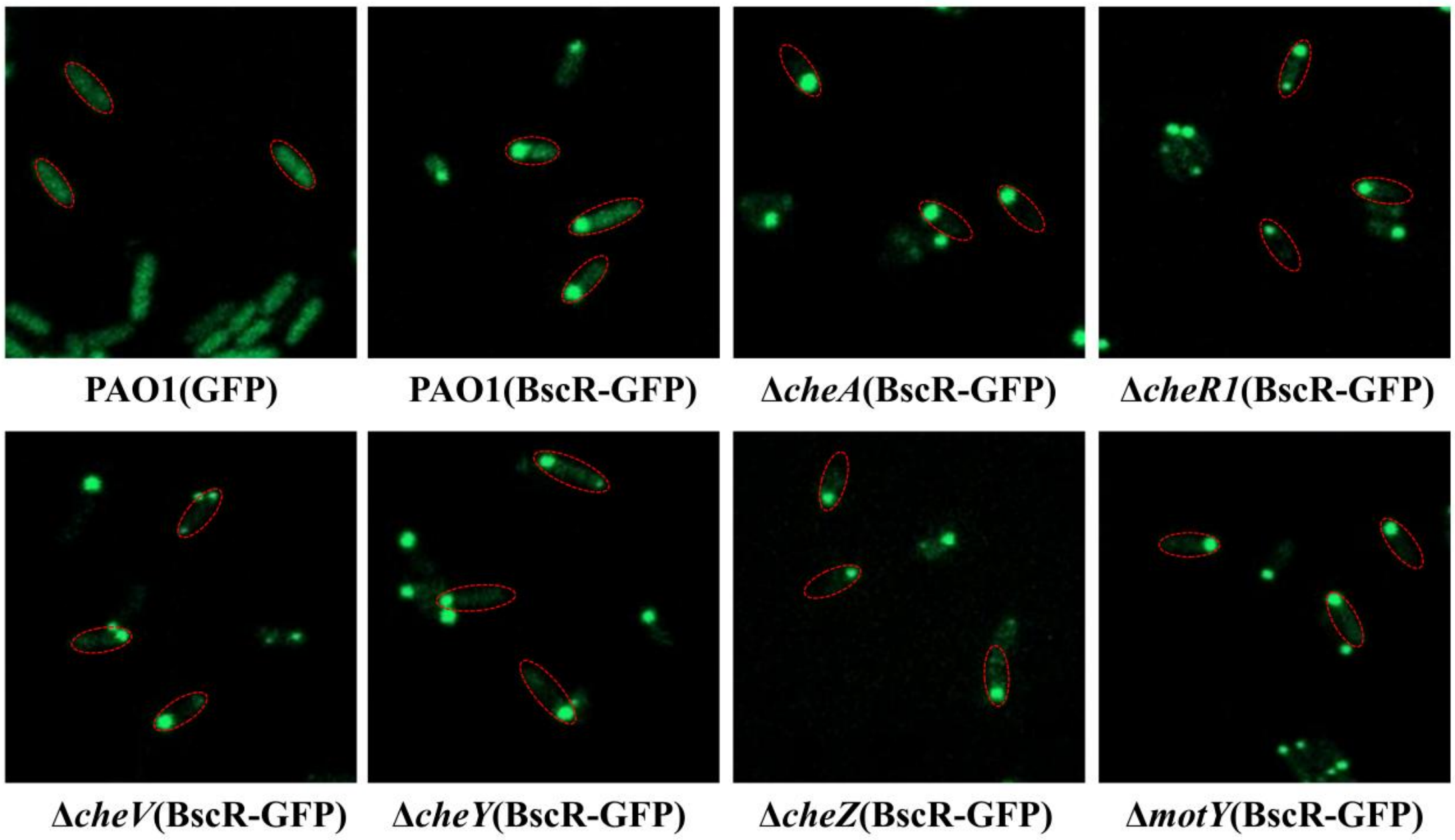

Fig. S11. Fluorescence microscopy images showing the intracellular localization of GFP in wild-type PAO1, as well as BscR-GFP in PAO1 and its mutants Δ*cheA*, Δ*cheR1*, Δ*cheV*, Δ*cheY*, Δ*cheZ*, Δ*motY*. All experiments were performed in triplicate.

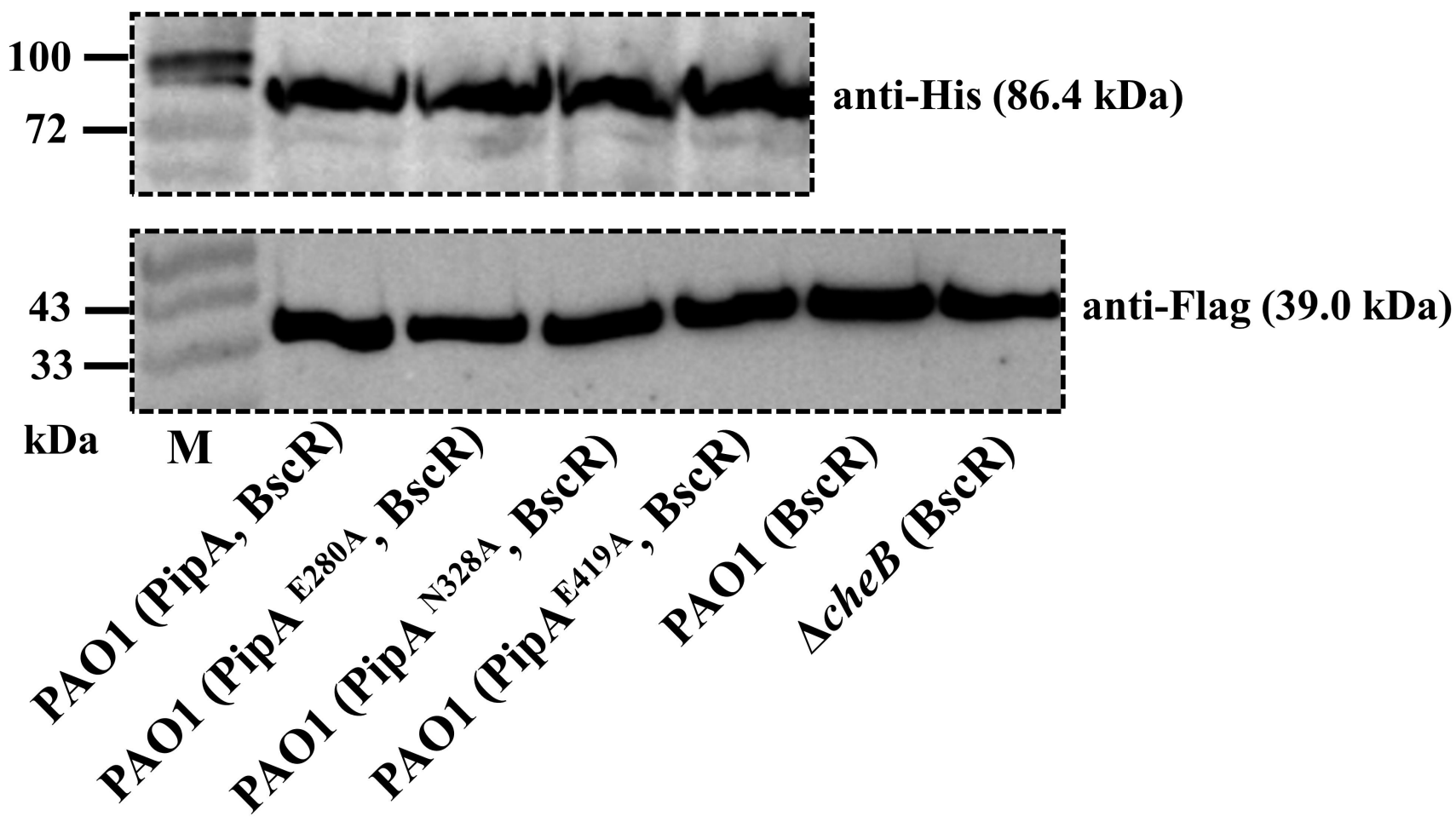

Fig. S12. Western blot validation of co-expression of PipA and BscR in engineered *P. aeruginosa* strains. All experiments were performed in triplicate.

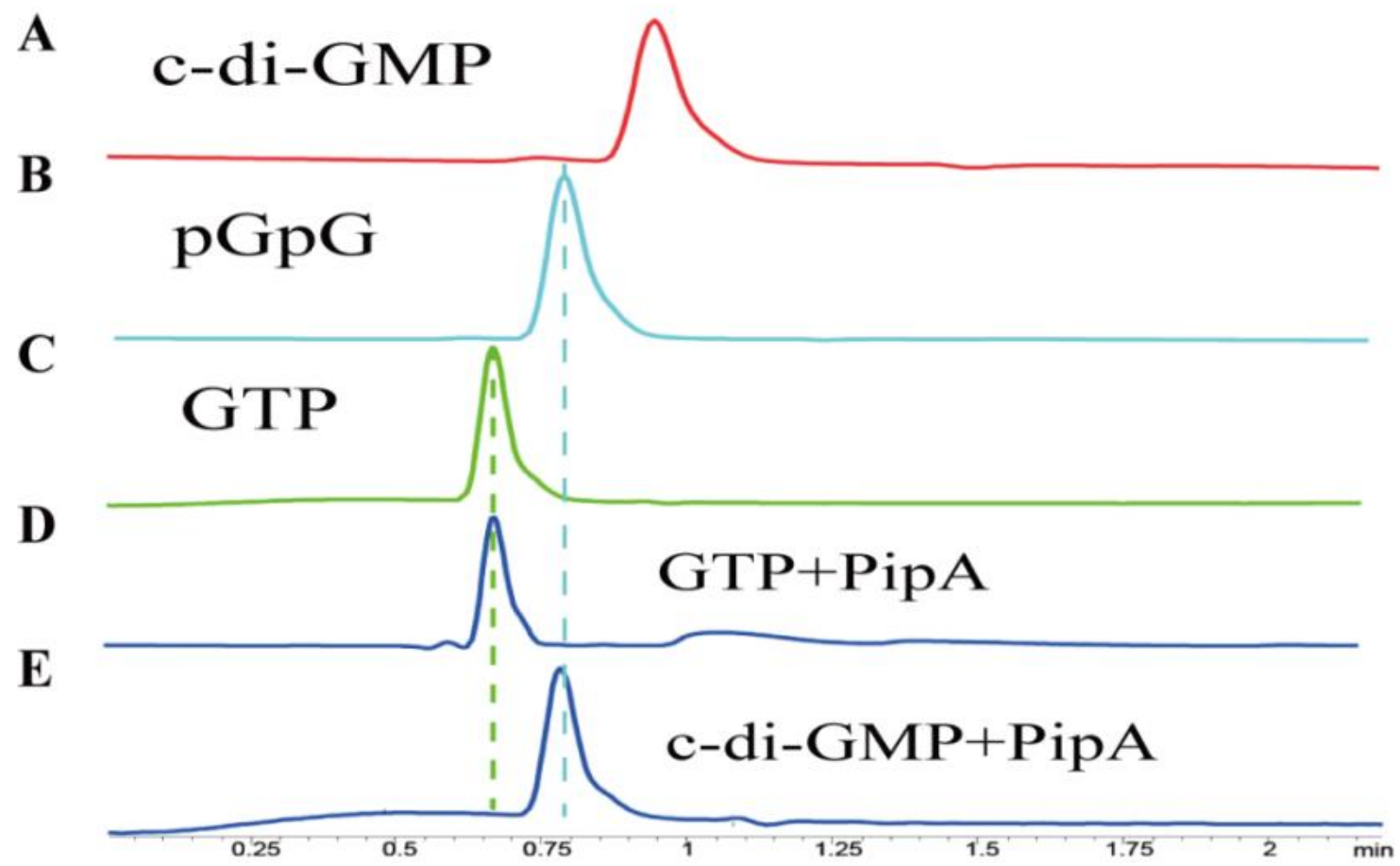

Fig. S13. The activity of PipA was assessed by HPLC. HPLC analysis determined retention times for: c-di-GMP (A), pGpG (B) and GTP (C). (D) Co-incubation of PipA with GTP was employed to determine its DGC activity. (E) The PDE activity of PipA was assessed through co-incubation with c-di-GMP *in vitro*. All experiments were performed in triplicate.

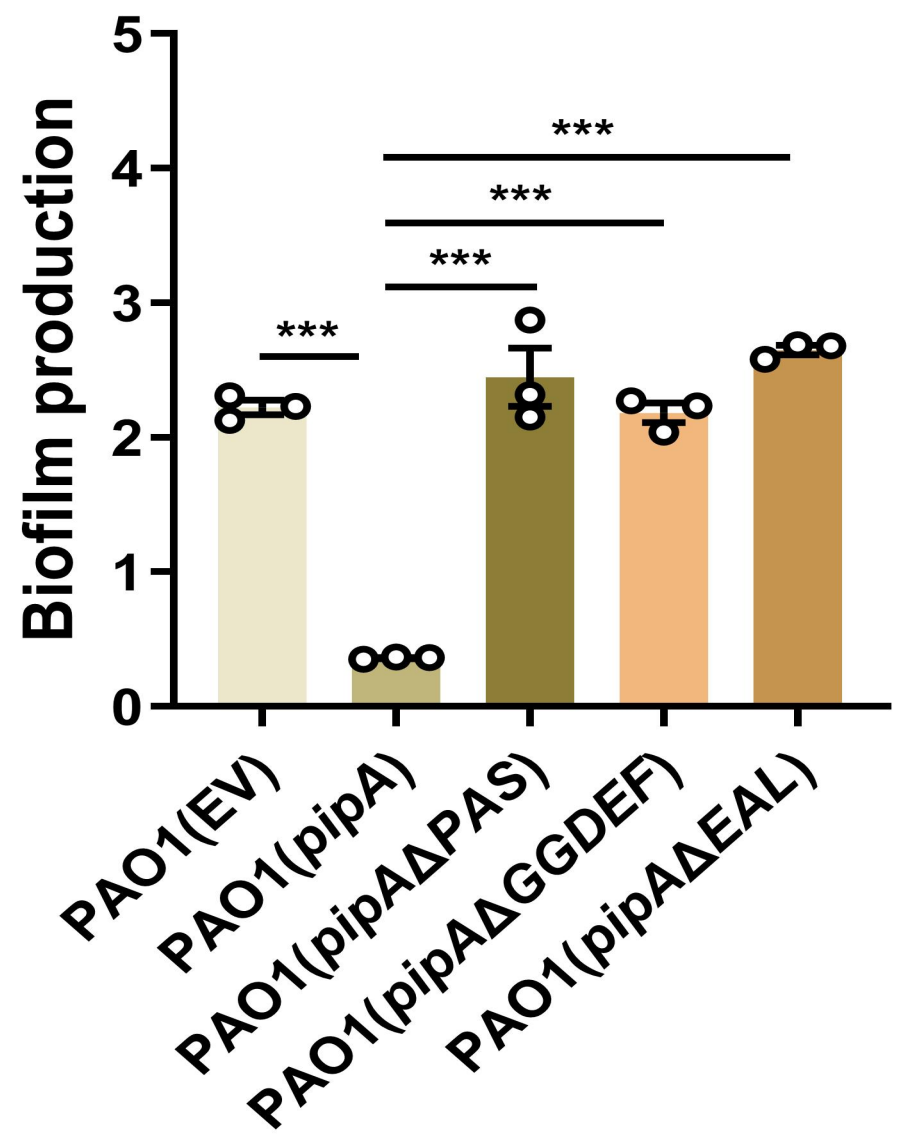

Fig. S14. Biofilm formation in the tested strains was quantified. Data are presented as mean ± SD (three independent experiments). ***, $P < 0.001$ *versus* the indicated group based on unpaired Student's *t* test. EV, empty vector for the control.

Table S1. Binding proteins were identified by Co-IP assay.

Protein ID	Protein Description	Credibility score	Sequence coverage	Domain structure
PA3309	conserved hypothetical protein	198.52	65	—
PA4723	suppressor protein DksA	175.68	41	—
PA0937	conserved hypothetical protein	156.9	26	—
PA2562	hypothetical protein	143.33	42	—
PA3965	probable AsnC family transcriptional regulator	123.1	34	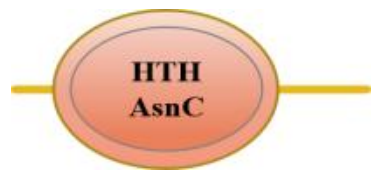
PA3196	hypothetical protein	119.35	19	—
PA5308	leucine-responsive regulatory protein	116.79	30	—
PA1899	phzA2 probable phenazine biosynthesis protein	108.47	27	—

Table S2. A comprehensive list of genes with differential expression (where the absolute value of Log2fold change is equal to or greater than 1.5) was obtained by comparing the Δ*bscR* mutant with the PAO1 strain. After Benjamini-Hochberg correction, Cufflinks was used to identify significantly differentially expressed genes. The fold change values were calculated as mutant FPKM/wild-type FPKM.

Locus	Product	Log2FoldChange
PA0135	hypothetical protein	-1.7904
PA0149	probable sigma-70 factor, ECF subfamily	2.4908
PA0150	anti-sigma factor	2.0260
PA0320	calcium-regulated OB-fold protein CarO	1.6426
PA0470	Ferrichrome receptor FiuA	2.4829
PA0471	FiuR	2.8732
PA0472	fiuI	2.6155
PA0500	biotin synthase	2.1768
PA0520	regulatory protein NirQ	2.7560
PA0521	probable cytochrome c oxidase subunit	2.2600
PA0522	hypothetical protein	2.4480
PA0527	transcriptional regulator Dnr	-3.7452
PA0545	hypothetical protein	-1.7834
PA0605	AgtC	-1.5402

PA0665	conserved hypothetical protein	1.7168
PA0668	tyrosyl-tRNA synthetase 2	-1.7883
PA0672	heme oxygenase	5.0044
PA0713	hypothetical protein	1.8298
PA0847	diguanylate cyclase	-1.5264
PA0929	two-component response regulator	1.7809
PA1077	flagellar basal-body rod protein FlgB	2.0251
PA1081	flagellar basal-body rod protein FlgF	2.1123
PA1082	flagellar basal-body rod protein FlgG	1.9641
PA1083	flagellar L-ring protein precursor FlgH	1.7759
PA1086	flagellar hook-associated protein FlgK	2.2461
PA1092	flagellin type B FliC	1.8679
PA1094	flagellar capping protein FliD	1.6542
PA1100	flagellar hook–basal body complex protein	2.0648
PA1101	flagella M-ring outer membrane protein precursor	1.8796
PA1102	flagellar motor switch protein	1.7864
PA1300	HxuI ECF sigma factor	4.1018
PA1301	HxuR	3.9623
PA1302	hxuA	1.9808
PA1313	probable major facilitator superfamily (MFS) transporter	1.6418
PA1316	probable major facilitator superfamily (MFS) transporter	2.0001
PA1429	probable cation-transporting P-type ATPase	-3.0059
PA1457	chemotaxis protein CheZ	2.1862
PA1458	probable two-component sensor CheA	2.3428
PA1459	probable methyltransferase CheB	2.8472
PA1460	flagellar motor component MotC	2.6271
PA1461	flagellar motor protein MotD	2.2569
PA1556	Cytochrome c oxidase, cbb3-type, CcoO subunit	-1.6839
PA1557	Cytochrome c oxidase, cbb3-type, CcoN subunit	-2.1645
PA1632	KdpF protein	1.5159
PA1847	NfuA	2.3119
PA1899	probable phenazine biosynthesis protein	-2.9812
PA1091	phenazine biosynthesis protein PhzC	-3.8751
PA1092	phenazine biosynthesis protein PhzD	-3.6951
PA1093	phenazine biosynthesis protein PhzE	-3.8163
PA1851	hypothetical protein	-1.6182
PA1905	probable pyridoxamine 5'-phosphate oxidase	-6.3639
PA1908	probable major facilitator superfamily (MFS) transporter	1.6704
PA1909	hypothetical protein	2.0129
PA1910	ferric-mycobactin receptor, FemA	-2.9405
PA1911	sigma factor regulator, FemR	-3.7132

PA1912	ECF sigma factor, FemI	-2.7178
PA1920	class III (anaerobic) ribonucleoside-triphosphate reductase subunit, NrdD	-1.6634
PA2033	hypothetical protein	4.2903
PA2034	hypothetical protein	4.0869
PA2127	hypothetical protein	-1.7719
PA2170	hypothetical protein	-2.6834
PA2193	hcnA	-1.7240
PA2194	hcnB	-1.5659
PA2232	pslB	-1.5382
PA2233	pslC	-1.4290
PA2235	pslE	-1.6523
PA2260	hypothetical protein	2.0283
PA2261	probable 2-ketogluconate kinase	1.7985
PA2320	transcriptional regulator GntR	2.2270
PA2321	GntK	2.8698
PA2322	gntP	2.3172
PA2384	PA2384	-1.7855
PA2385	pvdQ	-2.5947
PA2386	pvdA	-3.2693
PA2392	pvdP	-1.9029
PA2393	putative dipeptidase	-2.7381
PA2394	pvdN	-2.6550
PA2397	pvdE	-2.6825
PA2398	fpvA	-3.4166
PA2405	fpvJ	-1.7913
PA2406	fpvK	-1.7185
PA2411	probable thioesterase	3.2585
PA2412	conserved hypothetical protein	3.6882
PA2413	pvdH	-1.5180
PA2425	pvdG	-3.3480
PA2426	pvdS	-4.3678
PA2427	hypothetical protein	1.5112
PA2466	Ferrioxamine receptor FoxA	-2.2686
PA2467	Anti-sigma factor FoxR	-2.8931
PA2468	foxI	-3.0082
PA2566	conserved hypothetical protein	-1.7970
PA2567	hypothetical protein	1.5926
PA2630	conserved hypothetical protein	-1.6210
PA2662	conserved hypothetical protein	1.7091
PA2663	psl and pyoverdine operon regulator, PpyR	-1.6961
PA2664	flavohemoprotein	1.8344
PA2686	two-component response regulator PfeR	3.4226
PA2687	two-component sensor PfeS	2.9840

PA2688	Ferric enterobactin receptor, outer membrane protein PfeA precursor	2.3434
PA2691	conserved hypothetical protein	1.9067
PA2759	hypothetical protein	1.6573
PA3058	pelG	-1.9862
PA3060	pelF	-2.0148
PA3061	pelD	-2.1973
PA3062	pelC	-1.5628
PA3064	pelA	-1.7362
PA3126	heat-shock protein IbpA	1.6110
PA3235	conserved hypothetical protein	-1.5332
PA3278	hypothetical protein	-2.4278
PA3337	ADP-L-glycero-D-mannoheptose 6-epimerase rfaD	-3.3225
PA3348	probable chemotaxis protein CheR1	2.2637
PA3349	probable chemotaxis protein methyltransferase CheV	2.0649
PA3408	Heme uptake outer membrane receptor HasR precursor	-1.5662
PA3409	HasS	-2.8806
PA3410	HasI	-3.0986
PA3415	probable dihydrolipoamide acetyltransferase	2.0213
PA3416	probable pyruvate dehydrogenase E1 component, beta chain	1.9126
PA3431	hypothetical protein	2.4455
PA3432	hypothetical protein	2.5673
PA3526	probable outer membrane protein precursor MotY	2.4761
PA3530	bacterioferritin-associated ferredoxin Bfd	3.4012
PA3572	hypothetical protein	-1.9427
PA3574a	Uncharacterized protein	2.9426
PA3600	RpmJ2 Synonym: rpl36	4.1239
PA3601	RpmE2	3.9337
PA3613	hypothetical protein	-2.1686
PA3792	2-isopropylmalate synthase	3.5545
PA3809	ferredoxin [2Fe-2S]	1.9488
PA3810	hscA	2.5300
PA3811	hscB	2.7653
PA3812	iscA	3.2781
PA3813	iscU	3.3637
PA3814	iscS	3.4569
PA3815	iscR	3.4857
PA3870	molybdopterin biosynthetic protein A1 moaA1	2.6012
PA3871	probable peptidyl-prolyl cis-trans isomerase, PpiC-type	3.4665

PA3872	narI	2.9456
PA3873	narJ	3.1129
PA3874	narH	2.3929
PA3876	narK2	-2.3657
PA3877	narK1	-3.4686
PA3899	FecI	3.2552
PA3900	FecR	3.4744
PA3914	molybdenum cofactor biosynthetic protein A1 moeA1	-2.4943
PA3915	molybdopterin biosynthetic protein B1 moeB1	-2.3300
PA3920	CopA1	2.3583
PA3965	hypothetical transcriptional factor	-7.5477
PA3971	hypothetical protein	1.6673
PA4063	hypothetical protein	1.6354
PA4092	4-hydroxyphenylacetate 3-monooxygenase small chain	1.6366
PA4155	hypothetical protein	3.1125
PA4156	FvbA	2.9982
PA4158	ferric enterobactin transport protein FepC	-2.8061
PA4159	ferrienterobactin-binding periplasmic protein precursor FepB	-3.3757
PA4160	ferric enterobactin transport protein FepD	-2.6650
PA4161	ferric enterobactin transport protein FepG	-2.7767
PA4167	probable oxidoreductase	2.0344
PA4168	second ferric pyoverdine receptor FpvB	-2.3616
PA4210	probable phenazine biosynthesis protein PhzA	-3.0143
PA4211	probable phenazine biosynthesis protein PhzB	-2.8150
PA4213	phenazine biosynthesis protein PhzD	-2.6931
PA4216	probable pyridoxamine 5'-phosphate oxidase	-1.8972
PA4217	flavin-containing monooxygenase PhzS	-2.0163
PA4218	AmpP	3.1932
PA4219	AmpO	3.2628
PA4220	hypothetical protein	2.6331
PA4221	Fe(III)-pyochelin outer membrane receptor precursor, fptA	-4.0192
PA4225	pchF	-1.8393
PA4226	pchE	-2.9541
PA4227	transcriptional regulator PchR	-3.9978
PA4228	pchD	-3.6744
PA4229	pchC	-3.8987
PA4230	pchB	-4.1278
PA4231	pchA	-3.3737
PA4328	hypothetical protein	-1.6388
PA4348	conserved hypothetical protein	-2.8704

PA4467	hypothetical protein	4.9679
PA4468	sodM superoxide dismutase	5.2706
PA4469	hypothetical protein	6.6931
PA4470	fumC1 fumarate hydratase	6.3825
PA4471	hypothetical protein	6.6155
PA4516	hypothetical protein	1.5825
PA4542	ClpB protein	1.5305
PA4570	hypothetical protein	5.7893
PA4571	probable cytochrome c	-2.0585
PA4615	FprB	1.9147
PA4623	hypothetical protein	1.6649
PA4687	ferric iron-binding periplasmic protein HitA	1.6749
PA4694	ketol-acid reductoisomerase	2.4771
PA4695	ilvH acetolactate synthase isozyme III small subunit	2.1124
PA4696	ilvI acetolactate synthase large subunit	2.2734
PA4706	PhuV	-1.9478
PA4707	phuU PhuU	-1.5969
PA4708	phuT Heme-transport protein, PhuT	-3.4265
PA4709	phuS PhuS	-4.8085
PA4710	Heme/Hemoglobin uptake outer membrane receptor PhuR precursor	-2.3584
PA4823	hypothetical protein	-2.8376
PA4837	cntO TonB-dependent Outer Membrane Protein ZrmA	1.5582
PA4881	hypothetical protein	2.2138
PA4895	probable transmembrane sensor	4.4405
PA4896	probable sigma-70 factor, ECF subfamily	4.0986
PA4953	flagellar motor protein MotB	2.1579
PA4954	flagellar motor protein MotA	2.0158
PA5023	conserved hypothetical protein	2.7073
PA5027	hypothetical protein	-2.0937
PA5170	arginine/ornithine antiporter	-2.9805
PA5217	putative binding protein of iron ABC transporter	1.6875
PA5230	probable permease of ABC transporter	-1.7389
PA5231	probable ATP-binding/permease fusion ABC transporter	-1.6508
PA5232	conserved hypothetical protein	-2.0474
PA5275	conserved hypothetical protein	2.3021
PA5354	glycolate oxidase subunit GlcE	-1.6369
PA5475	hypothetical protein	-1.9841
PA5531	TonB1	-2.1783

Table S3. Bacterial strains and recombinant plasmids employed in this study.

Strains	Relevant genotype or phenotype	Source or reference
***P.aeruginosa* strains**		
PAO1	Wild type strain	Lab collection
Δ*bscR*	The *bscR* deletion mutant that the gene *PA3965* was deleted in strain PAO1	This study
Δ*pipA*	The *pipA* deletion mutant that the gene *PA0285* was deleted in strain PAO1	1
Δ*bscR*(*bscR*)	The *bscR* deletion mutant harboring the expression construct pUCP20-*bscR*	This study
Δ*pipA*(*pipA*)	The *pipA* deletion mutant harboring the expression construct pUCP20-*pipA*	This study
PAO1(*bscR*)	The wild type PAO1 harboring the expression construct pUCP20-*bscR* or pBBR1-MCS5-BscR	This study
PAO1(*pipA*)	The wild type PAO1 harboring the expression construct pUCP20-*pipA*	This study
Δ*bscR*(*pipA*)	The *bscR* deletion mutant harboring the expression construct pUCP20-*pipA*	This study
Δ*pipA*(*bscR*)	The *pipA* deletion mutant harboring the expression construct pUCP20-*bscR*	This study
Δ*pipA*Δ*bscR*	The double mutant that the genes *pipA* and *bscR* were deleted in strain PAO1	This study
Δ*pipA*Δ*bscR*(*bscR*)	The Δ*pipA*Δ*bscR* double mutant harboring the expression construct pUCP20-*bscR*	This study
PAO1(GFP)	The wild type PAO1 harboring the constructed pBBR1-MCS5-GFP	This study
Δ*bscR*(GFP)	The *bscR* deletion mutant harboring the constructed pBBR1-MCS5-GFP	This study
PAO1(BscR-GFP)	The wild type PAO1 harboring the constructed pBBR1-MCS5-BscR-GFP	This study
Δ*bscR*(BscR-GFP)	The *bscR* deletion mutant harboring the constructed pBBR1-MCS5-BscR-GFP	This study
Δ*cheB*	The *cheB* deletion mutant that the gene *PA1459* was deleted in strain PAO1	This study
Δ*cheW*	The *cheW* deletion mutant that the gene *PA1464* was deleted in strain PAO1	This study
Δ*cheZ*	The *cheZ* deletion mutant that the gene *PA1457* was deleted in strain PAO1	This study
Δ*cheA*	The *cheA* deletion mutant that the gene *PA1458* was deleted in strain PAO1	This study

Δ*cheR1*	The *cheR1* deletion mutant that the gene *PA3348* was deleted in strain PAO1	This study
Δ*cheV*	The *cheV* deletion mutant that the gene *PA3349* was deleted in strain PAO1	This study
Δ*cheY*	The *cheZ* deletion mutant that the gene *PA1456* was deleted in strain PAO1	This study
Δ*motY*	The *cheZ* deletion mutant that the gene *PA3526* was deleted in strain PAO1	This study
Δ*cheB*(*bscR*)	The *cheB* deletion mutant harboring the expression construct pBBR1-MCS5-BscR	This study
Δ*cheW*(*bscR*)	The *cheW* deletion mutant harboring the expression construct pBBR1-MCS5-BscR	This study
Δ*cheZ*(*bscR*)	The *cheZ* deletion mutant harboring the expression construct pBBR1-MCS5-BscR	This study
PAO1(*pipA*, *bscR*)	The wild type PAO1 harboring the constructed plasmids pUCP20-*pipA* and pBBR1-MCS5-BscR	This study
Δ*bscR*(*pipA*, *bscR*)	The *bscR* deletion mutant harboring the constructed plasmids pUCP20-*pipA* and pBBR1-MCS5-BscR	This study
Δ*bscR*(*pipA*^{E280A,N328A,E419}, *bscR*)	The *bscR* deletion mutant harboring the constructed plasmids pUCP20-*pipA*$^{E280A, N328A, E419}$ and pBBR1-MCS5-BscR	This study
Δ*bscR*(*pipA*^{E280A}, *bscR*)	The *bscR* deletion mutant harboring the constructed plasmids pUCP20-*pipA*^{E280A} and pBBR1-MCS5-BscR	This study
Δ*bscR*(*pipA*^{N328A}, *bscR*)	The *bscR* deletion mutant harboring the constructed plasmids pUCP20-*pipA*^{N328A} and pBBR1-MCS5-BscR	This study
Δ*bscR*(*pipA*^{E419A}, *bscR*)	The *bscR* deletion mutant harboring the constructed plasmids pUCP20-*pipA*^{E419} and pBBR1-MCS5-BscR	This study
PAO1(mCherry)	The wild type PAO1 harboring the constructed pUCP20-mCherry	This study
PAO1(CheB-mCherry)	The wild type PAO1 harboring the constructed pUCP20-CheB-mCherry	This study
PAO1(BscR-GFP, CheB-mCherry)	The wild type PAO1 harboring the constructed pBBR1-MCS5-BscR-GFP and pUCP20-CheB-mCherry	This study
Δ*pipA*(BscR-GFP, CheB-mCherry)	The *pipA* deletion mutant harboring the constructed pBBR1-MCS5-BscR-GFP and pUCP20-CheB-mCherry	This study

PAO1(*pipA*, BscR-GFP, CheB-mCherry)	The overexpression strain which Mini-CTX-Flag-*pipA* plasmid was integrate into PAO1 genome harboring the constructed pBBR1-MCS5-BscR-GFP and pUCP20-CheB-mCherry	This study
PAO1(PipA$^{E280A, N328A, E419}$, BscR-GFP)	The wild type PAO1 harboring the constructed plasmids pUCP20-*pipA*$^{E280A, N328A, E419}$ and pBBR1-MCS5-BscR-GFP	This study
PAO1(PipA^{E280A}, BscR-GFP)	The wild type PAO1 harboring the constructed plasmids pUCP20-*pipA*^{E280A} and pBBR1-MCS5-BscR-GFP	This study
PAO1(PipA^{N328A}, BscR-GFP)	The wild type PAO1 harboring the constructed plasmids pUCP20-*pipA*^{N328A} and pBBR1-MCS5-BscR-GFP	This study
PAO1(PipA^{E419}, BscR-GFP)	The wild type PAO1 harboring the constructed plasmids pUCP20-*pipA*^{E419} and pBBR1-MCS5-BscR-GFP	This study
PAO1(PipA, BscR-GFP)	The wild type PAO1 harboring the constructed pBBR1-MCS5-BscR-GFP and pUCP20-*pipA*	This study
Δ*cheB*(BscR-GFP)	The *cheB* deletion mutant harboring the constructed pBBR1-MCS5-BscR-GFP	This study
Δ*cheW*(BscR-GFP)	The *cheW* deletion mutant harboring the constructed pBBR1-MCS5-BscR-GFP	This study
Δ*cheZ*(BscR-GFP)	The *cheZ* deletion mutant harboring the constructed pBBR1-MCS5-BscR-GFP	This study
Δ*cheA*(BscR-GFP)	The *cheA* deletion mutant harboring the constructed pBBR1-MCS5-BscR-GFP	This study
Δ*cheR1*(BscR-GFP)	The *cheR1* deletion mutant harboring the constructed pBBR1-MCS5-BscR-GFP	This study
Δ*cheV*(BscR-GFP)	The *cheV* deletion mutant harboring the constructed pBBR1-MCS5-BscR-GFP	This study
Δ*cheY*(BscR-GFP)	The *cheY* deletion mutant harboring the constructed pBBR1-MCS5-BscR-GFP	This study
Δ*motY*(BscR-GFP)	The *motY* deletion mutant harboring the constructed pBBR1-MCS5-BscR-GFP	This study
***E. coli* strains**		
DH5α	F^-φ80 *lac*ZΔM15Δ(*lac*ZYA-*arg*F) U169 *end*A1 *rec*A1 *hsd*R17(rk^-,mk^+) *sup*E44λ- thi -1 *gyr*A96 *rel*A1 *pho*A	TransGen
RK600	ori(ColE1), RK2(mob+ tra+); Cm^r	Lab collection
BL21Star(λDE3)	F^-*omp*T hsdSB (rB^- mB^-) *gal dcm rne*131 (DE3)	Lab collection

**Plasmids**		
pUCP20	*E. coli*-*P. aeruginosa* shuttle expression vector with P_{lac}, Amp^r, Cb^r	Lab collection
Mini-CTX-Flag	Integration plasmid; Tc^r	Lab collection
pBBR1-MCS5	Broad-host-range cloning vector; Gm^r	Lab collection
pK18mobsacB	Broad-host-range gene replacement vector, sacB,Gm^r	Lab collection
pET28a	Expression vector with N-terminal His tag, Kan^r	Lab collection
pBT	p15A origin of replication, lac-UV5 promoter, λ cI open reading frame; Cm^r	Lab collection
pBT-LGF2	Interaction control plasmid encoding the dimerization domain (40 amino acid residues) of the Gal4 transcriptional activator protein; Cm^r	Lab collection
pTRG	ColE1 origin of replication, lpp promoter, lac-UV5 promoter, RNAPα open reading frame; Tc^r	Lab collection
pTRG-Gal11P	Interaction control plasmid encoding a domain (90 amino acid residues) of the mutant form of the Gal11 protein; T^{c_r}	Lab collection
pBBR1-MCS5-*bscR*	pBBR1-MCS5-containing *bscR* under control of P_{lac}	This study
pBBR1-MCS5-*bscR*-Flag	pBBR1-MCS5-containing *bscR* with a C-terminal Flag tag under control of P_{lac}	This study
pUCP20-*bscR*	pUCP20 containing *bscR* under control of P_{lac}	This study
pUCP20-*pipA*	pUCP20 containing *pipA* under control of P_{lac}	This study
pUCP20-*pipA*-His	pUCP20 containing *pipA* with a C-terminal His tag under control of Plac.	This study
pUCP20-*bscR*-Flag	pUCP20 containing *bscR* labelled with Flag tag under control of P_{lac}	This study
pUCP20-*cheB*-His	pUCP20 containing *cheB* labelled with His tag under control of P_{lac}	This study
pUCP20-$pipA^{E280A,\ N328A,\ E419}$	pUCP20 containing $pipA^{E280A,\ N328A,\ E419}$ which glutamate, asparagine and glutamate position at 280, 328 and 419, respectively, were replaced by alanine together	This study
pUCP20-$pipA^{E280A}$	pUCP20 containing $pipA^{E280A}$ which glutamate position at 280 was replaced by alanine	This study
pUCP20-$pipA^{E280A}$-His	pUCP20 containing $pipA^{E280A}$ with a C-terminal His tag, which glutamate position at 280 was replaced by alanine	This study

pUCP20-*pipA*^{N328A}	pUCP20 containing *pipA*^{N328A} with a C-terminal His tag, which asparagine position at 328 was replaced by alanine	This study
pUCP20-*pipA*^{N328A}-His	pUCP20 containing *pipA*^{N328A} with a C-terminal His tag, which glutamate position at 280 was replaced by alanine	This study
pUCP20-*pipA*^{E419}	pUCP20 containing *pipA*^{E419} with a C-terminal His tag, which glutamate position at 419 was replaced by alanine	This study
pUCP20-*pipA*^{E419}-His	pUCP20 containing *pipA*^{E419} which glutamate position at 419 was replaced by alanine	This study
pBBR1-MCS5-GFP	pBBR1-MCS5 containing *gfp* under control of P_{lac}	This study
pBBR1-MCS5-BscR-GFP	pBBR1-MCS5 containing *bscR* and *gfp* under control of P_{lac}	This study
pUCP20-mCherry	pUCP20 containing *mCherry* under control of P_{lac}	This study
pUCP20-CheB-mCherry	pUCP20 containing *cheB* and *mCherry* under control of P_{lac}	This study
Mini-CTX-Flag-*pipA*	Mini-CTX-Flag plasmid containing *pipA*	This study
pK18-*bscR*	*bscR* deletion plasmid	This study
pK18-*cheA*	*cheA* deletion plasmid	This study
pK18-*cheB*	*cheB* deletion plasmid	This study
pK18-*cheW*	*cheW* deletion plasmid	This study
pK18-*cheY*	*cheY* deletion plasmid	This study
pK18-*cheZ*	*cheZ* deletion plasmid	This study
pK18-*cheR1*	*cheR1* deletion plasmid	This study
pK18-*cheV*	*cheV* deletion plasmid	This study
pK18-*motY*	*motY* deletion plasmid	This study
pBT-BscR	pBT containing the *bscR* gene	This study
pBT-PipA	pBT containing the entire *pipA* gene	This study
pBT-BscR$^{H87A, H128A, T139A}$	pBT containing the *bscR*$^{H87A, H128A, T139A}$ gene which histidine, histidine and threonine position at 87, 128 and 139, respectively, were replaced by alanine together	This study
pBT-BscR^{H87A}	pBT containing with the *bscR*^{H87A} gene which histidine position at 87 was replaced by alanine	This study
pBT-BscR^{H128A}	pBT containing the *bscR*^{H128A} gene which histidine position at 128 was replaced by alanine	This study

pBT-BscR^{T139A}	pBT containing the *bscR*^{T139A} gene which threonine position at 139 was replaced by alanine	This study
pBT-PipA$^{E280A, N328A, E419A}$	pBT containing the *pipA*$^{E280A, N328A, E419A}$ gene which glutamate, asparagine and glutamate position at 280, 328 and 419, respectively, were replaced by alanine together	This study
pBT-PipA^{E280A}	pBT containing the *pipA*^{E280A} gene which glutamate position st 280 was replaced by alanine	This study
pBT-PipA^{N328A}	pBT containing the *pipA*^{N328A} gene which asparagine position at 328 was replaced by alanine	This study
pBT-PipA^{E419A}	pBT containing the *pipA*^{E419A} gene which glutamate position at 419 was replaced by alanine	This study
pTRG-BscR	pTRG with the *bscR* gene	This study
pTRG-CheB	pTRG with the *cheB* gene	This study
pTRG-PA3309	pTRG with the *PA3309* gene	This study
pTRG-PA4723	pTRG with the *PA4723* gene	This study
pTRG-PA0937	pTRG with the *PA0937* gene	This study
pTRG-PA2562	pTRG with the *PA2562* gene	This study
pTRG-PA5308	pTRG with the *PA5308* gene	This study
pET28a-*pipA*	The *pipA* was clone into pET28a	This study

Symbol: Gm^r, gentamicin resistance; Cb^r, carbenicillin resistance; Kan^r, kanamycin resistance; Amp^r, ampicillin resistance; Cm^r, chloramphenicol resistance; Tc^r, tetracycline resistance.

References

1. Cai, Y.M., Yu, K.W., Liu, J. H., Cai, Z., Zhou, Z.H., Liu, Y., Wang, T. F., Yang, L., 2022. The c-di-GMP phosphodiesterase PipA (PA0285) regulates autoaggregation and Pf4 bacteriophage production in *Pseudomonas aeruginosa* PAO1. Appl Environ Microbiol. 88(12), e0003922. https://doi:10.1128/aem.00039-22.

Table S4. Oligonucleotide primers designed for targeted amplification.

Primers	Sequence (5'-3')	Application
bscR-Up-F	gagctcggtacccggggatccTCGGCCTGCGCCTGGACG	For amplification of the 5'-region of *bscR*
bscR-Up-R	ctacgaCGGCAAGGTGAATTTGAGTGA	
bscR-Dw-F	aattcaccttgccgTCGTAGCGAGACCTGGGACA	For amplification of the 3'-region of *bscR*
bscR-Dw-R	acgacggccagtgccaagcttTGTTCTAGGCAATGGCAAGGC	
bscR-F	gagctcggtacccggggatccGCGGGCAAAGAAAAAGGG	For construction of pUCP20-*bscR*
bscR-R	acgacggccagtgccaagcttTATACGGCATGCCGCCTG	
bscR-5F	gtcgacggtatcgataagcttGCGGGCAAAGAAAAAGGG	For construction of pBBR1-MCS5-*bscR*
bscR-5R	cgctctagaactagtggatccTCAATCGCGCAGGTGCTC	
bscR-R(Flag)	cgctctagaactagtggatccTTTATCATCATCATCCTTATAATCTCAATCGCGCAGGTGCT	For construction of C-terminally Flag-tagged BscR
pipA-F	gagctcggtacccggggatccGTGCACGGTGGGCGGAATAG	For construction of pUCP20-*pipA*
pipA-R	acgacggccagtgccaagcttTCAGTCTTCCGGCAGCGCCAC	
pipA-R(His)	acgacggccagtgccaagcttGTGGTGGTGGTGGTGGTGTCAGTCTTCCGGCAGCGCCAC	For construction of C-terminally His-tagged PipA
pUCP-$pipA^{3m}$-2	tcttgcgacggttccagatGGCTCCGCTCCAGACGTCCTGC	For amplification of first fragment of pUCP20-$pipA^{E280A, N328A, E419}$ with primer *pipA*-F
pUCP-$pipA^{3m}$-3	aGCCATCTGGAACCGTCGCAAGAG	For amplification of second fragment of pUCP20-$pipA^{E280A, N328A, E419}$
pUCP-$pipA^{3m}$-4	aggaaGGCCAGTTCGTTCTCCGAGCGC	
pUCP-$pipA^{3m}$-5	tcggagaacgaactgGCCTTCCTCGCCCACCACGAC	For amplification of third fragment of pUCP20-$pipA^{E280A, N328A, E4199}$
pUCP-$pipA^{3m}$-6	gttGGCGACGAGGATGGCGAATTCG	
pUCP-$pipA^{3m}$-7	aattcgccatcctcgtcGCCAACGACGACCCGGAAGCC	For amplification of fourth fragment of pUCP20-$pipA^{E280A, N328A, E419}$ with primer *pipA*-R
pUCP-*pipA*-E280A-2	tcttgcgacggttccagatGGCTCCGCTCCAGACGTCCTGC	For amplification of first fragment of

		pUCP20-*pipA*^{E280A} with primer *pipA*-F
pUCP-*pipA*-E280A-3	aGCCATCTGGAACCGTCGCAAGAG	For amplification of second fragment of pUCP20-*pipA*^{E280A} with primer *pipA*-R
pUCP-*pipA*-N328-2	aggaaGGCCAGTTCGTTCTCCGAGCGC	For amplification of first fragment of pUCP20-*pipA*^{N328A} with primer *pipA*-F
pUCP-*pipA*-N328-3	tcggagaacgaactgGCCTTCCTCGCCCACCACGAC	For amplification of second fragment of pUCP20-*pipA*^{N328A} with primer *pipA*-R
pUCP-*pipA*-E419-2	gttGGCGACGAGGATGGCGAATTCG	For amplification of first fragment of pUCP20-*pipA*^{E419} with primer *pipA*-F
pUCP-*pipA*-E419-3	aattcgccatcctcgtcGCCAACGACGACCCGGAAGCC	For amplification of second fragment of pUCP20-*pipA*^{E419} with primer *pipA*-R
mini-pipA-F	ctatagggcgaattgggtaccGTGCACGGTGGGCGGAAT	For construction of Mini-CTX-Flag-*pipA*
mini-pipA-R	gtcgaattcaagcttctcgagTCAGTCTTCCGGCAGCGC	
pipA-Pro-F	atgggtcgcggatccgaattcATGAGCCCCCGCCTGAGT	For construction of pET28a-*pipA*
pipA-Pro-R	gtggtggtggtggtgctcgagTCAGTCTTCCGGCAGCGC	
bscR-Flag-F	agctcggtacccggggatccTATGGCAATCAACCTGAAAGA	For construction of pUCP20-*bscR*-Flag
bscR-Flag-R	cgacggccagtgccaagcttTCACTTGTCATCGTCGTCCTTGTAGTCATCGCGCAGGTGCTCCAG	
cheB-His-F	gagctcggtacccggggatccTATGGCAGTCAAAGTCCTGGTGGTG	For construction of pUCP20-*cheB*-His
cheB-His-R	acgacggccagtgccaagcttTCAATGGTGATGGTGATGATGCTCGAGACTAGTCACGCCTCGACCAGGTGCC	
cheB-Up-F	gagctcggtacccggggatccAAGATCGTCATCAAGGTGCCG	For amplification of the 5'-region of *cheB*
cheB-Up-R	AAGATCGTCATCAAGGTGCC	
cheB-Dw-F	tgaggagtagctGCGTGCGTGCAGGAATGA	For amplification of the 3'-region of *cheB*
cheB-Dw-R	acgacggccagtgccaagcttGACTTTCCTGGGTGAACAGGTC	

cheW-Up-F	gagctcggtacccggggatccCGGAGCCCTTCGAGTGCC	For amplification of the 5’-region of *cheW*
cheW-Up-R	tagaacgcaTCCCCTAACCTCTCGAAAACC	
cheW-Dw-F	aggttaggggaTGCGTTCTACCGACACGAGG	For amplification of the 3’-region of *cheW*
cheW-Dw-R	acgacggccagtgccaagcttCACTGGCAGAAGTGGCGAGC	
cheZ-Up-F	gagctcggtacccggggatccCCTGAACCGGCCTCGACC	For amplification of the 5’-region of *cheZ*
cheZ-Up-R	GACACCCTCTTGGCCGGC	
cheZ-Dw-F	aagccggccaagagggtgtcCCCGCCCGGCTCAAGACT	For amplification of the 3’-region of *cheZ*
cheZ-Dw-R	acgacggccagtgccaagcttGTTGACCGTATCCAGCGCC	
cheY-Up-F	gagctcggtacccggggatccAGAACCGGGCGCGACGCT	For amplification of the 5’-region of *cheY*
cheY-Up-R	cggctGTTCTTGTCCAAGTAAACCTCCG	
cheY-Dw-F	tacttggacaagaacAGCCGGCCAAGAGGGTGT	For amplification of the 3’-region of *cheY*
cheY-Dw-R	acgacggccagtgccaagcttCGGCGTTCTCGCCGCTGC	
cheV-Up-F	gagctcggtacccggggatccCTCCAGCACGTGCGCGCG	For amplification of the 5’-region of *cheV*
cheV-Up-R	ttgtcgagggccgatcgcTGGCGCTCCCCTCAGACG	
cheV-Dw-F	caGCGATCGGCCCTCGACAA	For amplification of the 3’-region of *cheV*
cheV-Dw-R	acgacggccagtgccaagcttCGACGATCTGCACGCCCG	
cheA-Up-F	gagctcggtacccggggatccTCCTCGCAGCTCAATGACATC	For amplification of the 5’-region of *cheA*
cheA-Up-R	aaacccatacgcggcgTCGGCTGCTCCCAGAGACG	
cheA-Dw-F	ccgaCGCCGCGTATGGGTTTCC	For amplification of the 3’-region of *cheA*
cheA-Dw-R	acgacggccagtgccaagcttGCTGGACGAGGTGGCCGA	
cheR1-Up-F	gagctcggtacccggggatccCGAGACCCAGAGCCGCGC	For amplification of the 5’-region of *cheR1*
cheR1-Up-R	cttccttgcgGCATAAGCCTCTTCGCCCTG	
cheR1-Dw-F	aggcttatgcCGCAAGGAAGCGGACCCG	For amplification of the 3’-region of *cheR1*
cheR1-Dw-R	acgacggccagtgccaagcttGAGCATGCCGAGGGCCGA	
motY-Up-F	gagctcggtacccggggatccGCTTCGAAGATATTGTCGATGCG	For amplification of the 5’-region of *motY*
motY-Up-R	ttctatcgtttgcgcgacGACGGCGGGCTCCGGGAA	
motY-Dw-F	tcGTCGCGCAAACGATAGAATCTG	For amplification of the 3’-region of *motY*
motY-Dw-R	acgacggccagtgccaagcttCGGACCTGGTCGTTGCCC	

pBT-*bscR*-F	gaagagacgtttggcgcggccgcAATGGCAATCAACCTGAAAGAAATC	For construction of pBT-*bscR*
pBT-*bscR*-R	ctcgaggatccccgggaattcTCAATCGCGCAGGTGCTC	
pBT-*bscR*^{3m}-2	ggtGGCTCGGTCCATGCCTATGAGGA	For amplification of first fragment of pBT-BscR$^{H87A, H128A, T139A}$ with primer pBT-*bscR*-F
pBT-*bscR*^{3m}-3	tcataggcatggaccgaGCCACCCCGGAACGCTTCGAA	For amplification of second fragment of pBT-BscR$^{H87A, H128A, T139A}$
pBT-*bscR*^{3m}-4	ctcgatgcgGGCCAGGGTACCCAGCAGGAGTTTCTGGTAATGGGCCATGTCCGGCACCACGAC	
pBT-*bscR*^{3m}-5	tgccggacatgGCCCATTACCAGAAACTCCTGCTGGGTACCCTGGCCCGCATCGAGGGGGTCTCC	For amplification of third fragment of pBT-BscR$^{H87A, H128A, T139A}$ with primer pBT-*bscR*-R
pBT-*bscR*-H87A-2	ggtGGCTCGGTCCATGCCTATGAGGA	For amplification of first fragment of pBT-BscR^{H87A} with primer pBT-*bscR*-F
pBT-*bscR*-H87A-3	tcataggcatggaccgaGCCACCCCGGAACGCTTCGAA	For amplification of second fragment of pBT-BscR^{H87A} with primer pBT-*bscR*-R
pBT-*bscR*-H128A-2	ttctggtaatgGGCCATGTCCGGCACCACGAC	For amplification of first fragment of pBT-BscR^{H128A} with primer pBT-*bscR*-F
pBT-*bscR*-H128A-3	ccggacatgGCCCATTACCAGAAACTCCTGCTGGG	For amplification of second fragment of pBT-BscR^{H128A} with primer pBT-*bscR*-R
pBT-*bscR*-T139A-2	GGCCAGGGTACCCAGCAGGAGTTT	For amplification of first fragment of pBT-BscR^{T139A} with primer pBT-*bscR*-F
pBT-*bscR*-T139A-3	aactcctgctgggtaccctgGCCCGCATCGAGGGGGTCTCC	For amplification of second fragment of pBT-BscR^{T139A} with primer pBT-*bscR*-R
pBT-*pipA*-F	gaagagacgtttggcgcggccgcAATGAGCCCCCGCCTGAGT	For construction of pBT-*pipA*
pBT-*pipA*-R	ctcgaggatccccgggaattcTCAGTCTTCCGGCAGCGC	

pBT-*pipA*-^{3m}-2	tcttgcgacggttccagatGGCTCCGCTCCAGACGTCCTGC	For amplification of first fragment of pBT-PipA$^{E280A, N328A, E419A}$ with primer pBT-*pipA*-F
pBT-*pipA*-^{3m}-3	aGCCATCTGGAACCGTCGCAAGAG	For amplification of second fragment of pBT-PipA$^{E280A, N328A, E419A}$
pBT-*pipA*-^{3m}-4	aggaaGGCCAGTTCGTTCTCCGAGCGC	
pBT-*pipA*-^{3m}-5	tcggagaacgaactgGCCTTCCTCGCCCACCACGAC	For amplification of third fragment of pBT-PipA$^{E280A, N328A, E419A}$
pBT-*pipA*-^{3m}-6	gttGGCGACGAGGATGGCGAATTCG	
pBT-*pipA*-^{3m}-7	aattcgccatcctcgtcGCCAACGACGACCCGGAAGCC	For amplification of fourth fragment of pBT-PipA$^{E280A, N328A, E419A}$ with primer pBT-*pipA*-R
pBT-*pipA*-E280A-2	tcttgcgacggttccagatGGCTCCGCTCCAGACGTCCTGC	For amplification of first fragment of pBT-PipA^{E280A} with primer pBT-*pipA*-F
pBT-*pipA*-E280A-3	aGCCATCTGGAACCGTCGCAAGAG	For amplification of second fragment of pBT-PipA^{E280A} with primer pBT-*pipA*-R
pBT-*pipA*-N328-2	aggaaGGCCAGTTCGTTCTCCGAGCGC	For amplification of first fragment of pBT-PipA^{N328A} with primer pBT-*pipA*-F
pBT-*pipA*-N328-3	tcggagaacgaactgGCCTTCCTCGCCCACCACGAC	For amplification of second fragment of pBT-PipA^{N328A} with primer pBT-*pipA*-R
pBT-*pipA*-E419-2	gttGGCGACGAGGATGGCGAATTCG	For amplification of first fragment of pBT-PipA^{E419A} with primer pBT-*pipA*-F
pBT-*pipA*-E419-3	aattcgccatcctcgtcGCCAACGACGACCCGGAAGCC	For amplification of second fragment of pBT-PipA^{E419A} with primer pBT-*pipA*-R
pTRG-*bscR*-F	gaggcggccggatccgcggccgcGATGGCAATCAACCTGAAAGAAATC	For construction of pTRG-*bscR*
pTRG-*bscR*-R	gcgccagctcagactgaattcTCAATCGCGCAGGTGCTC	

pTRG-*cheB*-F	gaggcggccggatccgcggccgcGATGGCAGTCAAAGTCCTGGTGG	For construction of pTRG-*cheB*
pTRG-*cheB*-R	gcgccagctcagactgaattcTTACTGGCACGCCTCGACC	
pTRG-*PA3309*-F	gaggcggccggatccgcggccgcATGTACGATCGCATTCTGGTAGC	For construction of pTRG-*PA3309*
pTRG-*PA3309*-R	gcgccagctcagactgaattcTTATTTGTCGCGGACCAGCA	
pTRG-*PA4723*-F	gaggcggccggatccgcggccgcATGTCCACCAAAGCAAAACAACA	For construction of pTRG-*PA4723*
pTRG-*PA4723*-R	gcgccagctcagactgaattcTCAGGAGCCGAGTTGCTTCTC	
pTRG-*PA0937*-F	gaggcggccggatccgcggccgcATGAGCATGTCATTGCGTGACC	For construction of pTRG-*PA0937*
pTRG-*PA0937*-R	gcgccagctcagactgaattcTCACCACATGAGATCGTCGGG	
pTRG-*PA2562*-F	gaggcggccggatccgcggccgcATGTTCAACACTCGTTCTGTGCTC	For construction of pTRG-*PA2562*
pTRG-*PA2562*-R	gcgccagctcagactgaattcTCAGTCGAGGACGTTGTCCAG	
pTRG-*PA5308*-F	gaggcggccggatccgcggccgcATGCGTACCCAGCATCAGAGC	For construction of pTRG-*PA5308*
pTRG-*PA5308*-R	gcgccagctcagactgaattcTCAATCCGGAACCGGTAGG	
pBBR1-GFP-F	gtcgacggtatcgataagcttATGGTGAGCAAGGGCGAGG	For construction of pBBR1-MCS5-GFP
pBBR1-GFP-R	cgctctagaactagtggatccTTACTTGTACAGCTCGTCCATGCC	
pBBR1-BscR-GFP-1	gtcgacggtatcgataagcttGATGGCAATCAACCTGAAAGAAA	For amplification of the *bscR* of pBBR1-MCS5-BscR-GFP
pBBR1-BscR-GFP-2	ttgctcaccatGGTGGCGACATCGCGCAGGTGCTCCAG	
pBBR1-BscR-GFP-3	ctgcgcgatGTCGCCACCATGGTGAGCAAGGGCGAGG	For amplification of the *gfp* of pBBR1-MCS5-BscR-GFP
pBBR1-BscR-GFP-4	cgctctagaactagtggatccTTACTTGTACAGCTCGTCCATGCC	
pUCP-mCherry-F	gagctcggtacccggggatccTATGGTGAGCAAGGGCGAGG	For construction of pUCP2-mCherry
pUCP-mCherry-R	acgacggccagtgccaagcttTTACTTGTACAGCTCGTCCATGCC	
pUCP-mCherry-CheB-1	gagctcggtacccggggatccTATGGCAGTCAAAGTCCTGGTGG	For amplification of the *cheB* of pUCP20-CheB-mCherry
pUCP-mCherry-CheB-2	ttgctcaccatGGTGGCGACCTGGCACGCCTCGACCAG	

pUCP-mCherry-CheB-3	gcgtgccagGTCGCCACCATGGTGAGCAAGGGCGAGG	For amplification of the *mCherry* of pUCP20-CheB-mCherry with primer pUCP-mCherry-R
For RT-qPCR		
q-*flgB*-F	CGACAGAGCACTCGGTATCC	
q-*flgB*-R	TTGTAGTTCGGGGTATCGGC	
q-*flgF*-F	GTGCTCATGCCAACAACCTG	
q-*flgF*-R	ATGGCTGAAATCGGTACCGG	
q-*fliA*-F	GAGCAACTGATCCAGCGTTACG	
q-*fliA*-R	TATTTCTTCGCGGCCTCGAG	
q-*fliC*-F	CCTGCAGCAGTCCACCAATA	
q-*fliC*-R	CGGAGATACGGGTCAGTTCG	
q-*fliD*-F	CCTTGCAGAAGACCCTCAGCA	
q-*fliD*-R	TGTCCGTGATGCTCTTCTGACG	
q-*fliE*-F	CGTCTGATGCTGGAAATGCG	
q-*fliE*-R	GTCTCGTTCACCTTGTCCACG	
q-*fliG*-F	TGATCCGTTACGAACACCCG	
q-*fliG*-R	ACCTTGTGGTCGAAATGGCTG	
q-*fliM*-F	AGTCCTATGACCTGACCAGCCAG	
q-*fliM*-R	GTTGAACATGCTGATGCGGG	
q-*fliK*-F	ACGCCTGATGTAAAGCCCAAG	
q-*fliK*-R	GCGGTCTCCTTGGCATACATG	
q-*pvdA*-F	CAACCCCACCAGTCCGTATT	
q-*pvdA*-R	AGTCGTTGAACTCCATCCGG	
q-*pvdE*-F	CAGGTGGTGACCATCCTTCC	
q-*pvdE*-R	GCTGAGCAGAAGATGGGGTT	
q-*pvdG*-F	TTGCTACCCCTGGAATTGCC	
q-*pvdG*-R	CGTAGGGAGAAGCGCTATCG	
q-*pvdS*-F	GGTCCAGGATGCGTTCTTCA	
q-*pvdS*-R	GGTAGTGATCGATGGCCAGG	
q-*pchA*-F	AGGAACACTGGCAAGCCTTC	
q-*pchA*-R	GTTGGCAGAGTACCCGGTAG	
q-*pchB*-F	GAGGAAAACGGACTCGACGC	
q-*pchB*-R	GTCTGGCGCCAGTACTTGAT	
q-*pchE*-F	AGCCGTTCGAGCTGTCTTC	
q-*pchE*-R	GTGCGGAATTCCAGAAAGGC	
q-*pchR*-F	ATCCGCGGATCATGAGCTG	
q-*pchR*-R	GAAAGGAACTGTGCGCTGAG	
q-*pipA*-F	GGCTCAAGGAAGACCACCTG	
q-*pipA*-R	TCGAAGGAAGGGTTGACGTG	
q-*bscR*-F	CATAGGCATGGACCGACACA	
q-*bscR*-R	TAATGGTCCATGTCCGGCAC	
q-*cheA*-F	CGTGGGTTCCATACGGTCAAG	

q-*cheA*-R	TGCGCAGGATATCGAAGACG
q-*cheB*-F	CCGGATCAACGTCAAGGAGG
q-*cheB*-R	CCGTCGACCATCATCTGCTT
q-*cheR1*-F	GAGAAGACCTGCGGCATTGTG
q-*cheR1*-R	CTCTCCCAGGCTCTTGATGCC
q-*cheZ*-F	TTCCAGATCGACCCGCATTC
q-*cheZ*-R	GGCCTTCTCGGTCATTTCCA
q-*cheV*-F	CGACGTGGAAAAAGTGCTCG
q-*cheV*-R	CAACGGATTACCTGCTTGCG
q-*motA*-F	CTCGGCATCGTCATCACCAT
q-*motA*-R	CGAGAATCCCGAGGAAGGTG
q-*motC*-F	CTGTTCACCCAGGAAAGTCGC
q-*motC*-R	CATTACCGCGCCGATGATG
q-*motY*-F	AATTTCGGAGTCGGCGAGTT
q-*motY*-R	CCAAGGTTGATGTCGCCCT
q-*pelA*-F	TCGATACCCTGGACAGCTTCC
q-*pelA*-R	GAAAAACAGCTTGAGGCCCG
q-*pelB*-F	CGCATCATGTTCTTCGCCTG
q-*pelB*-R	CTGCCGGTGAACCAGAAGATC
q-*pelC*-F	ATGCAATCCATCCGCTGC
q-*pelC*-R	CCTGGGAATAATTGAGCAGC
q-*phzA1*-F	GGATCCGAACCACTTCTGGG
q-*phzA1*-R	ACGTTTTATCCGGCCGTTCT
q-*phzB1*-F	TGGCAAAGACAAACTTGCGG
q-*phzB1*-R	ATGATTGGGGTCGTCGGTTC
q-*phzC1*-F	CAACATCATGCGCCACCTG
q-*phzC1*-R	GTCGAACCGAGATAGACCCG
q-*cdrA*-F	CCAGTTCAACCCCAACGAGA
q-*cdrA*-R	GTCGAAGCCCTTCCAGTTGA
q-*rplU*-F	AGGCGAATTCCTCAAGGTCG
q-*rplU*-R	AGCGGTAACCTTCGCACCTT

Original images of swimming and swarming motility assays from three independent biological replicates.

Fig. 1D

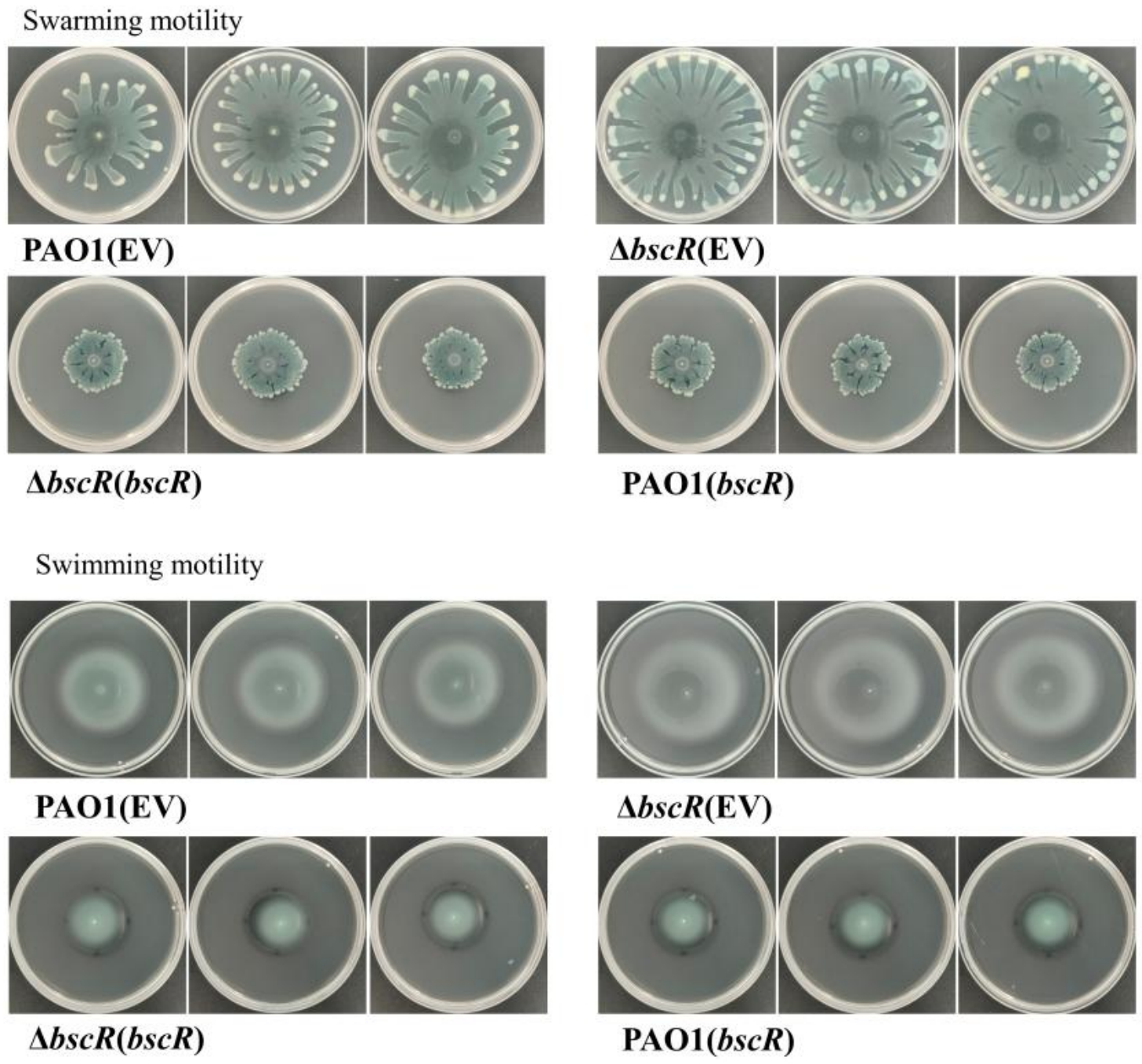

Fig. 3A

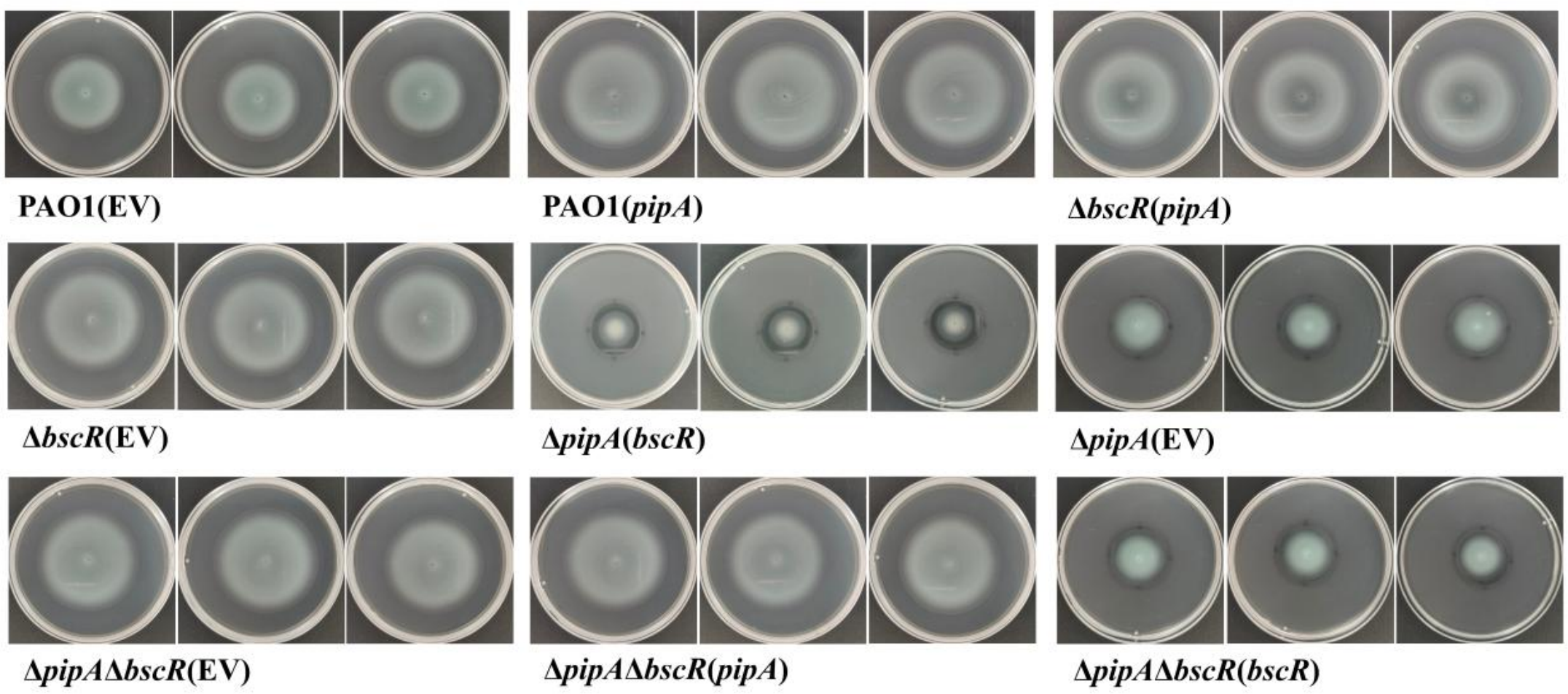

Fig. 4G

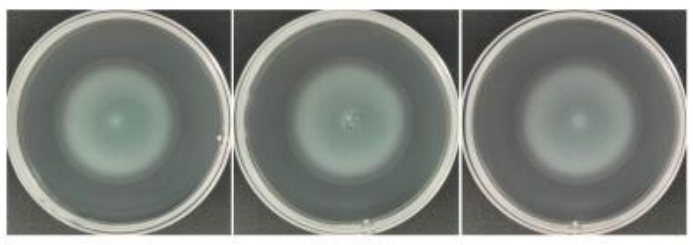

PAO1(EV)

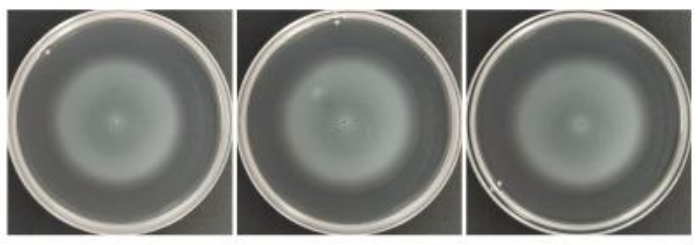

Δ*bscR*(EV)

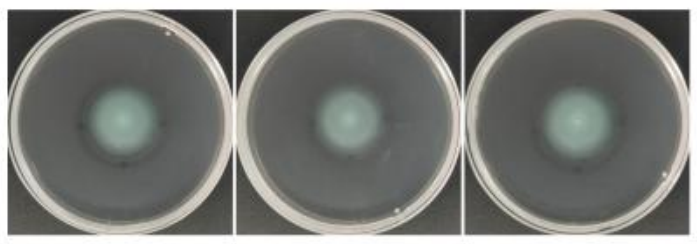

Δ*bscR*(*bscR*)

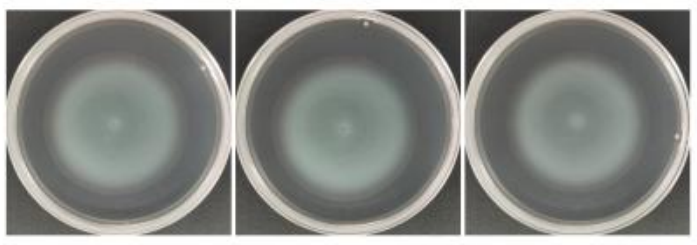

Δ*bscR*(*pipA*, *bscR*)

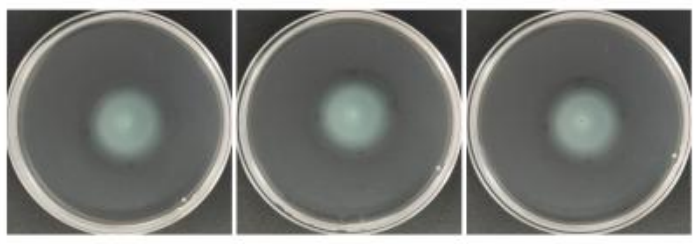

Δ*bscR*(*pipA*^{E280A,N328A,E419A}, *bscR*)

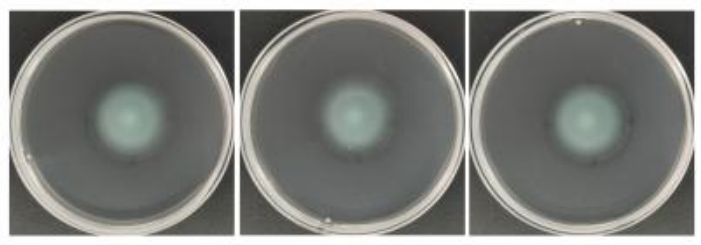

Δ*bscR*(*pipA*^{E280A}, *bscR*)

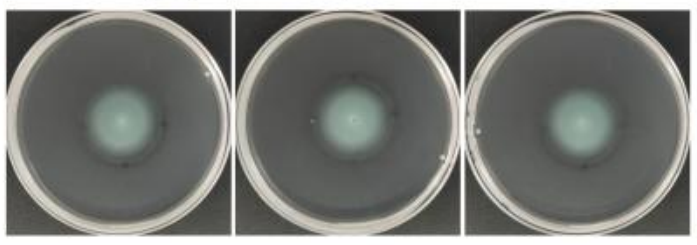

Δ*bscR*(*pipA*^{N328A}, *bscR*)

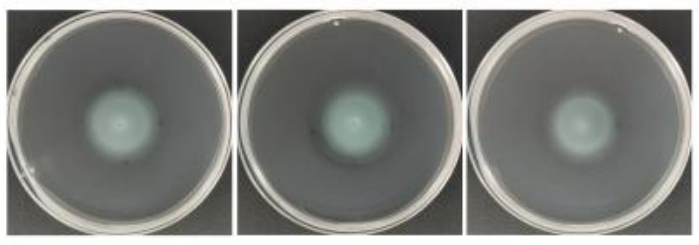

Δ*bscR*(*pipA*^{E419A}, *bscR*)

Fig. 5C

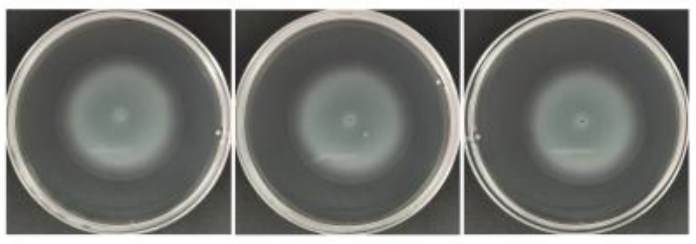

PAO1(EV)

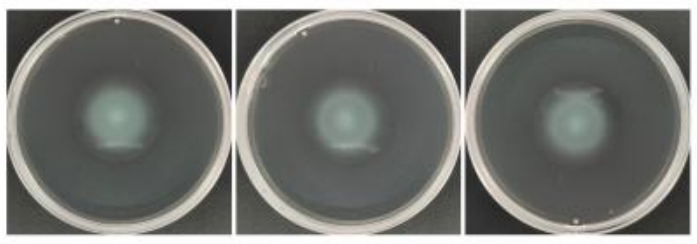

PAO1(*bscR*)

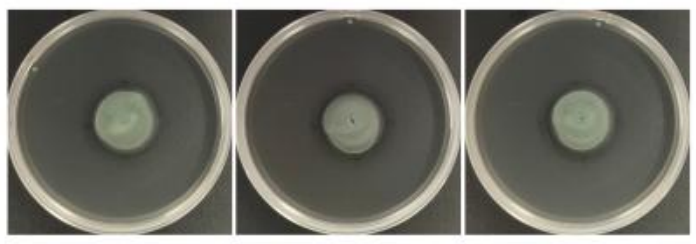

Δ*cheB*(EV)

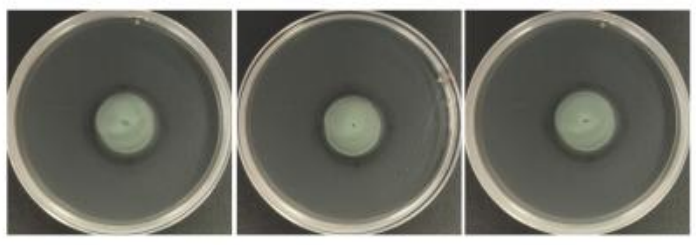

Δ*cheB*(*bscR*)

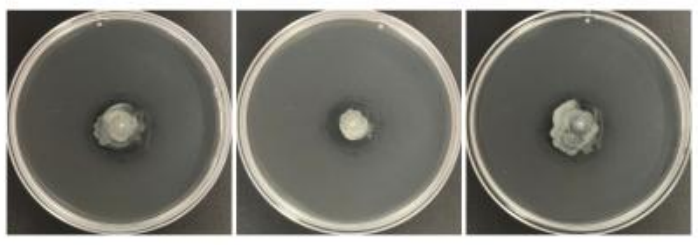

Δ*cheW*(EV)

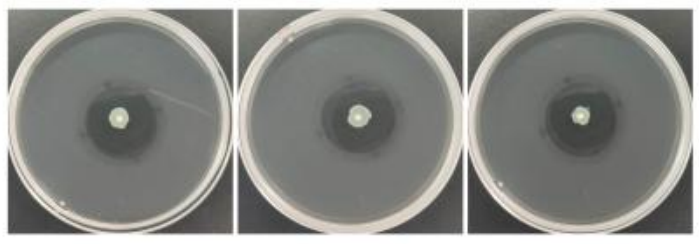

Δ*cheW*(*bscR*)

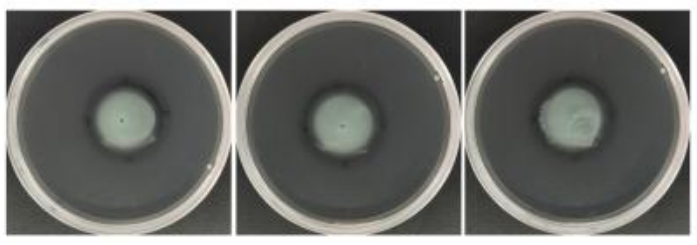

Δ*cheZ*(EV)

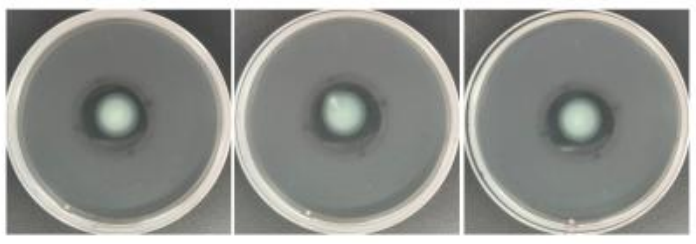

Δ*cheZ*(*bscR*)

Fig. S6

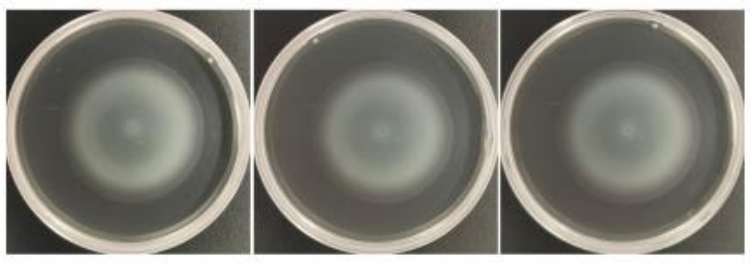

PAO1(GFP)

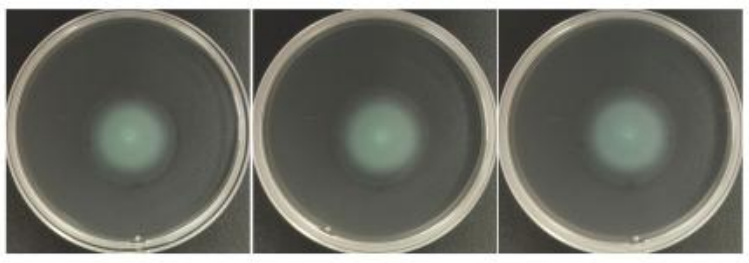

PAO1(BscR)

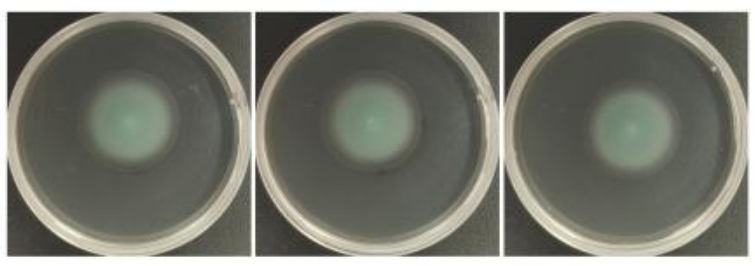

PAO1(BscR-GFP)

Fig. S7A

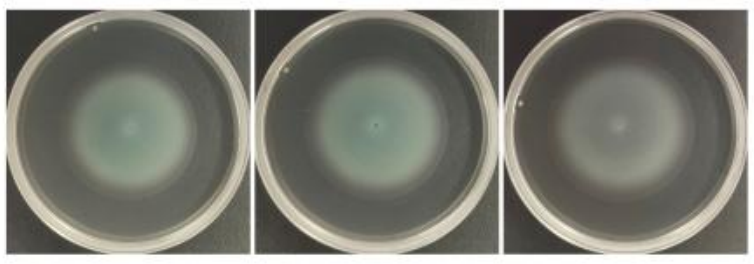

PAO1(EV)

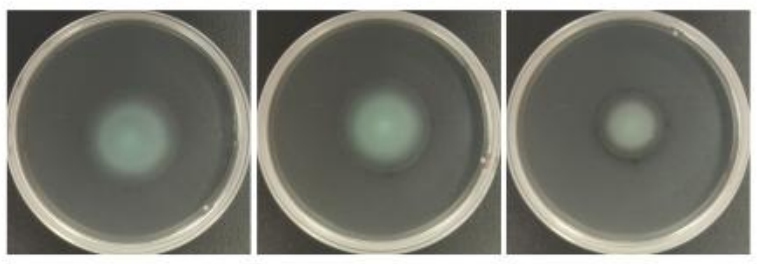

PAO1(*bscR*)

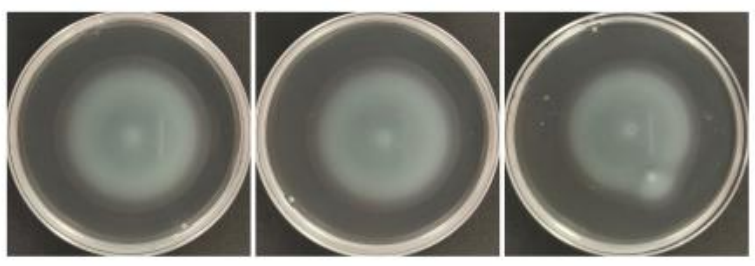

PAO1(*pipA*, *bscR*)
